# Cattle feces are a reservoir of diverse *Acinetobacter* species with potential to spread antibiotic resistance genes

**DOI:** 10.1186/s42523-026-00568-3

**Published:** 2026-04-22

**Authors:** Anitha Ravi, Violetta Shestivska, Priscila Thiago Dobbler, Hana Sechovcová, Martina Maixnerová, Jaroslav Semerád, Alena Nehasilová, Mariana Vadroňová, Iñaki Odriozola, Hana Šubrtová Salmonová, Tomáš Větrovský, Šárka Musilová, Tomáš Cajthaml, Eva Pěchoučková, Alexandr Nemec, Martina Kyselková

**Affiliations:** 1 Laboratory of Environmental Microbiology, Institute of Microbiology of the Czech Academy of Sciences, Vídeňská 1083, Prague 4, 142 20 Czech Republic; 2 Faculty of Science, Charles University in Prague, Albertov 6, Prague 2, 128 00 Czech Republic; 3 Laboratory of Bacterial Genetics, Centre for Epidemiology and Microbiology, National Institute of Public Health, Šrobárova 48, Prague 10, 100 00 Czech Republic; 4 Department of Microbiology, Nutrition and Dietetics, Faculty of Agrobiology, Food and Natural Resources, Czech University of Life Sciences, Kamýcká 129, Prague 6, 165 00 Czech Republic; 5 Laboratory of Anaerobic Microbiology, Institute of Animal Physiology and Genetics, Academy of Sciences of the Czech Republic, Vídeňská, 1083, Prague 4, 142 20 Czech Republic; 6 Laboratory of Environmental Biotechnology, Institute of Microbiology of the Czech Academy of Sciences, Vídeňská 1083, Prague 4, 142 20 Czech Republic; 7 Institute for Environmental Studies, Faculty of Science, Charles University in Prague, Benátská 2, Prague 2, 128 01 Czech Republic; 8 Department of Medical Microbiology, Second Faculty of Medicine, Charles University and Motol University Hospital, V Úvalu 84, Prague 5, 150 06 Czech Republic

**Keywords:** Antibiotic susceptibility, Carbapenemase, Cattle farm, Diversity, Identification, MALDI-TOF MS, Metabarcoding, Metagenomics, *rpoB*

## Abstract

**Background:**

Antibiotic resistance poses a major threat to human health, with antibiotic use in livestock contributing to the selection and spread of resistance genes. The genus *Acinetobacter* includes human- and animal-associated species capable of acquiring resistance, yet their diversity and resistance potential in livestock remain far less explored than in humans. In this study, we investigated *Acinetobacter* in cattle feces from 28 Czech farms with contrasting antibiotic use, aiming to assess species composition, resistance profiles, and the potential for resistance dissemination. We applied an integrative approach combining strain isolation and characterization, enrichment cultures, metabarcoding, and shotgun metagenomics.

**Results:**

Cattle feces harbored diverse *Acinetobacter* species with *A. indicus* and *A. pseudolwoffii* being the core species based on both isolated strains and metabarcoding, while *A. baumannii* was less common. *Acinetobacter* species occurrence determined by metabarcoding was driven by multiple factors, including production type, herd size, and per-head antibiotic use, while their abundance was mostly influenced by sample type (higher in feces from the farm floor than in rectal samples) and production type (higher in dairy than in beef cattle). Remarkably, 37% of the 284 isolated strains could not be assigned to validly named species and represent at least 19 putative novel species. Decreased susceptibility due to acquired resistance was observed in 57 strains; notably, *A. indicus* and *A. pseudolwoffii* from antibiotic-using farms were less susceptible to streptomycin than those from antibiotic-free farms. Shotgun metagenomics revealed a greater richness of acquired resistance genes in antibiotic-using farms, including the clinically relevant carbapenemase gene *bla*_OXA-58_. This gene was located on putative plasmid contigs alongside streptomycin resistance determinants *strA*-*strB*, suggesting horizontal dissemination under streptomycin selection pressure. Strain analysis confirmed the co-localization of *bla*_OXA-58_ and *strA*-*strB* on a large plasmid in *A. pseudolwoffii*.

**Conclusions:**

Despite relatively strict regulations, Czech cattle farms constitute a reservoir of antibiotic-resistant *Acinetobacter* carrying mobile resistance genes of clinical concern. Commonly applied antibiotics likely co-select for such genes, posing an ongoing public health risk. Our findings reveal an unexpectedly high diversity of *Acinetobacter* spp. in cattle, highlighting the research bias toward human-associated species and underscoring the need for integrated One Health monitoring approaches.

**Supplementary information:**

The online version contains supplementary material available at 10.1186/s42523-026-00568-3.

## Background

The long-term use of antibiotics in livestock production has been a major driver of antibiotic-resistant bacteria and antibiotic resistance genes (ARGs), which can be disseminated into the environment through practices such as the application of manure to agricultural soils [1-3]. Among livestock sectors, cattle farming is particularly significant, accounting for more than 50% of global antimicrobial use between 2019 and 2021 [4]. As part of the European Union’s broader One Health strategy, the use of antibiotics for growth promotion in livestock was banned in 2006, with further restrictions on prophylactic and metaphylactic applications introduced in 2022. However, antibiotics are still widely used for disease treatment on cattle farms, and their impact on the cattle gut resistome – and the potential risks this poses to public health – remains poorly understood.

Among antibiotic-resistant bacteria associated with cattle, *Acinetobacter baumannii* is of particular concern. This opportunistic pathogen is a leading cause of nosocomial infections, and certain strains exhibit resistance to virtually all available antibiotics, including last-resort drugs such as carbapenems and colistin [5]. The emergence of multidrug-resistant (MDR) strains, i.e., strains showing acquired non-susceptibility to agents in multiple antimicrobial classes, reflects the remarkable genomic plasticity of *A. baumannii* and its ability to accumulate resistance determinants, often via mobile genetic elements including insertion sequences, transposons, integrons and plasmids [6]. Several cultivation-based studies have documented the occurrence of *A. baumannii* in cattle, with isolates obtained from nasal, oral, rectal, and fecal samples (e.g. [7–10]). Two large-scale studies from Germany and South Korea reported *A. baumannii* in 15.6% and 2.6% of cattle, respectively, suggesting an overall low prevalence in cattle populations [7, 10]. Most cattle-associated strains belonged to novel multilocus sequence types [8, 9], but some were genetically linked to sequence types known from human clinical isolates [7, 10]. In addition, although the majority of cattle-derived strains were wild-type susceptible to antibiotics [7, 10], carbapenem-resistant *A. baumannii* has also been isolated from cattle, harboring clinically relevant ARGs such as *bla*_OXA-23_, *bla*_OXA-24_, and *bla*_OXA-58_ [11, 12].

Carbapenem-resistant *Acinetobacter* species other than *A. baumannii* have also been reported on cattle farms, including *Acinetobacter indicus* [13] or *Acinetobacter variabilis* [14], as well as potential novel species [15]. Moreover, several recent studies from China have documented MDR *Acinetobacter* spp. from cattle farms, carrying clinically relevant ARGs on plasmids, chromosomes, or both (e.g. [13, 15, 16]). For instance, co-localization of *tet*(X3) (tigecycline resistance) with *bla*_NDM-1_ or *bla*_OXA-58_ (carbapenem resistance) along with three additional ARGs on plasmids was shown in MDR *A. indicus* from a dairy farm [13]. In contrast, such MDR *Acinetobacter* spp. have not been reported in European cattle, but it remains unclear whether this difference reflects more restricted antibiotic use or simply a lack of comparable studies in Europe.

Taken together, these studies suggest that non-*baumannii Acinetobacter* species may play an important role in the dissemination of ARGs in farm environments. Moreover, some of these species, such as *A. variabilis*, are also recognized opportunistic human pathogens [17]. Nevertheless, current knowledge of *Acinetobacter* spp. in cattle and their potential for ARG dissemination remains fragmented and largely disconnected. For instance, *Acinetobacter*-specific LowGC-type plasmids were previously shown to be important for the spread of ARGs, including the tigecycline resistance gene *tet*(X), from livestock manure to soils [18–20], but it remains unclear whether such plasmids are also present in the *tet*(X3)-positive isolates from Chinese farms and which host species they are associated with.

Moreover, the co-selection of ARGs with heavy metal resistance genes (HMRGs) in the farm environment should be taken into account [21]. HMRGs are often found alongside ARGs on *Acinetobacter* plasmids and genomic islands, both in clinical *A. baumannii* and in farm-associated non-*baumannii Acinetobacter* spp. [13, 22]. In addition, heavy metals such as Cu, Zn, Pb, As, and Cr are commonly present in cattle feces and manure, originating either from dietary supplements or feed contamination [2, 23]. Consequently, the presence of these metals may exert additional selective pressure [21], promoting the development of antibiotic resistance in *Acinetobacter* spp. within the cattle gastrointestinal tract.

Overall, comprehensive data on the abundance, species composition, and antibiotic resistance of *Acinetobacter* in cattle feces and manure are still lacking. Limited insights are available from studies of Pulami et al. [24, 25] on German biogas plants processing cattle manure. For instance, the abundance of *Acinetobacter* spp. in input manure material was estimated at 10^6^−10^8^ 16S rRNA gene copies per gram of fresh weight [24], yet data on their abundance in fresh cattle feces remain unavailable. Factors influencing *Acinetobacter* species occurrence in cattle have so far been examined only for *A. baumannii* [7]. In the studied German cattle cohort, *A. baumannii* was more common in dairy than in beef cattle and calves, and its prevalence correlated with the use of third-generation cephalosporins in the preceding six months. A seasonal peak in isolation rates during summer was also observed [7], suggesting an effect of temperature and humidity on cattle colonization with *A. baumannii*. Yet, comparable data for other *Acinetobacter* species are still lacking. Therefore, we aimed to comprehensively investigate how antibiotic use and other farm- and cow-specific factors affect the abundance and diversity of *Acinetobacter* spp. in cattle feces, and to assess the contribution of antibiotic use to the acquisition of antibiotic resistance.

We hypothesized that (i) the composition of *Acinetobacter* species is influenced by cattle type and prior antibiotic administration, (ii) on-farm antibiotic use selects for resistant and MDR *Acinetobacter* strains carrying horizontally acquired ARGs, and (iii) heavy metals present in cattle feces further co-select for ARGs. To test these hypotheses, we applied an integrative approach combining selective enrichment culture and characterization of *Acinetobacter* strains with culture-independent methods and metagenomics. We used fecal samples collected from farm floors and individual cows across 28 Czech cattle farms with varying levels of antibiotic use. Isolated strains were taxonomically characterized and screened for antibiotic susceptibility as well as horizontally acquired ARGs. Enrichment cultures were analyzed for *Acinetobacter* diversity using *rpoB* metabarcoding and for resistome composition using shotgun metagenomics. The abundance of *Acinetobacter* spp. was quantified by qPCR from total fecal DNA. All analyses were complemented with farm metadata and sample chemical characteristics, including antibiotic residues and heavy metal content. Together, this study provides a comprehensive characterization of *Acinetobacter* diversity and antibiotic resistance in cattle feces.

## Methods

### Farms, sampling, and sample processing

**Fig. 1:**
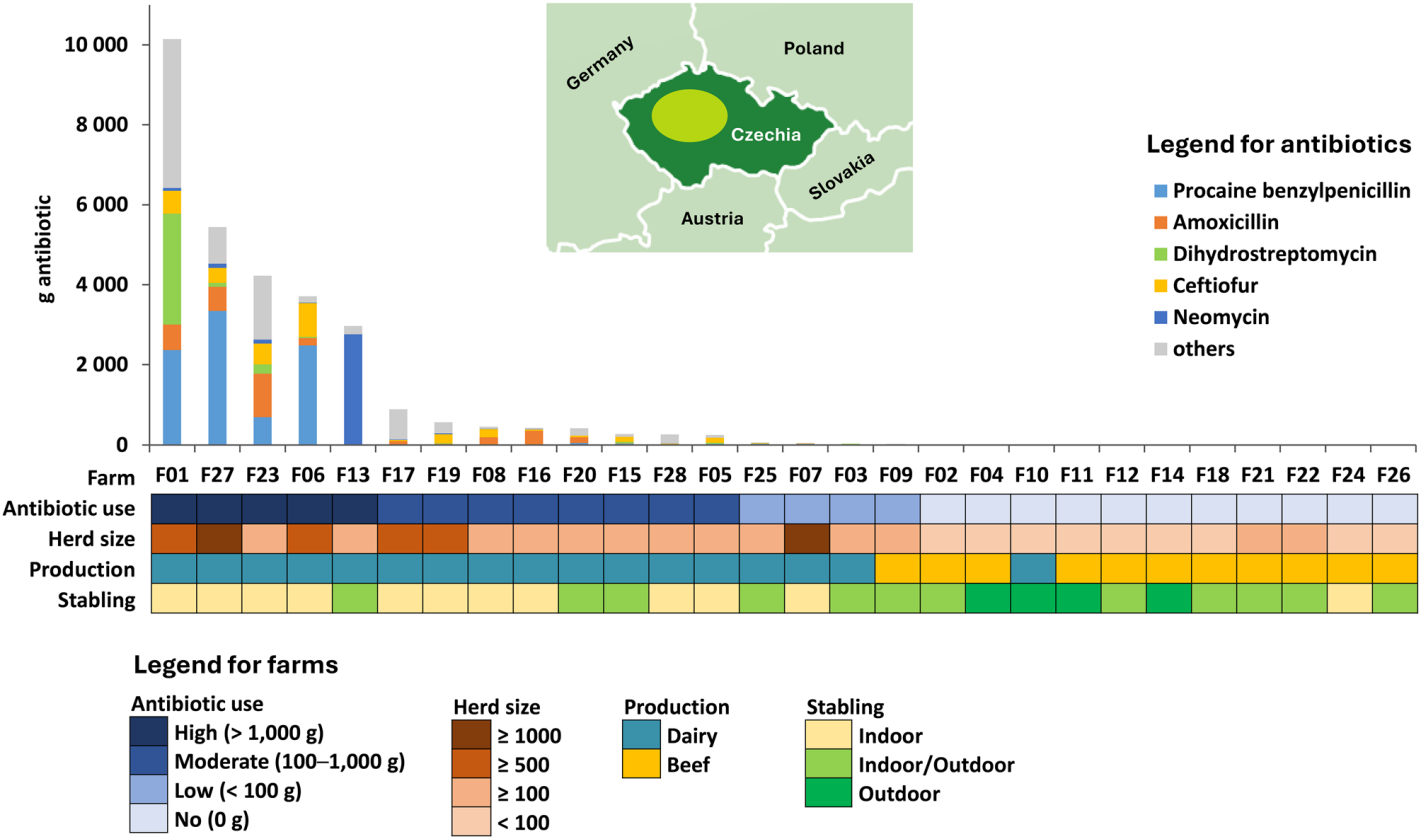
Overview of cattle farms. Twenty-eight anonymous cattle farms (referred to as F01–F28) were sampled in Czechia, mostly in the region of Central Bohemia (the approximate sampling area is shown as a light green oval on the map). Total on-farm antibiotic use during six months preceding the sampling is displayed in the bar plot, with main antibiotic types represented by colors. Based on the total antibiotic use, the farms were grouped into No (0 g), Low (<100 g), Moderate (100–1,000 g), and High (>1,000 g) antibiotic use categories (first row on the heatmap). The next rows of the heatmap show the categorization of farms according to the herd size, main production type and stabling type. More details can be found in tables S1–S3

**Fig. 2:**
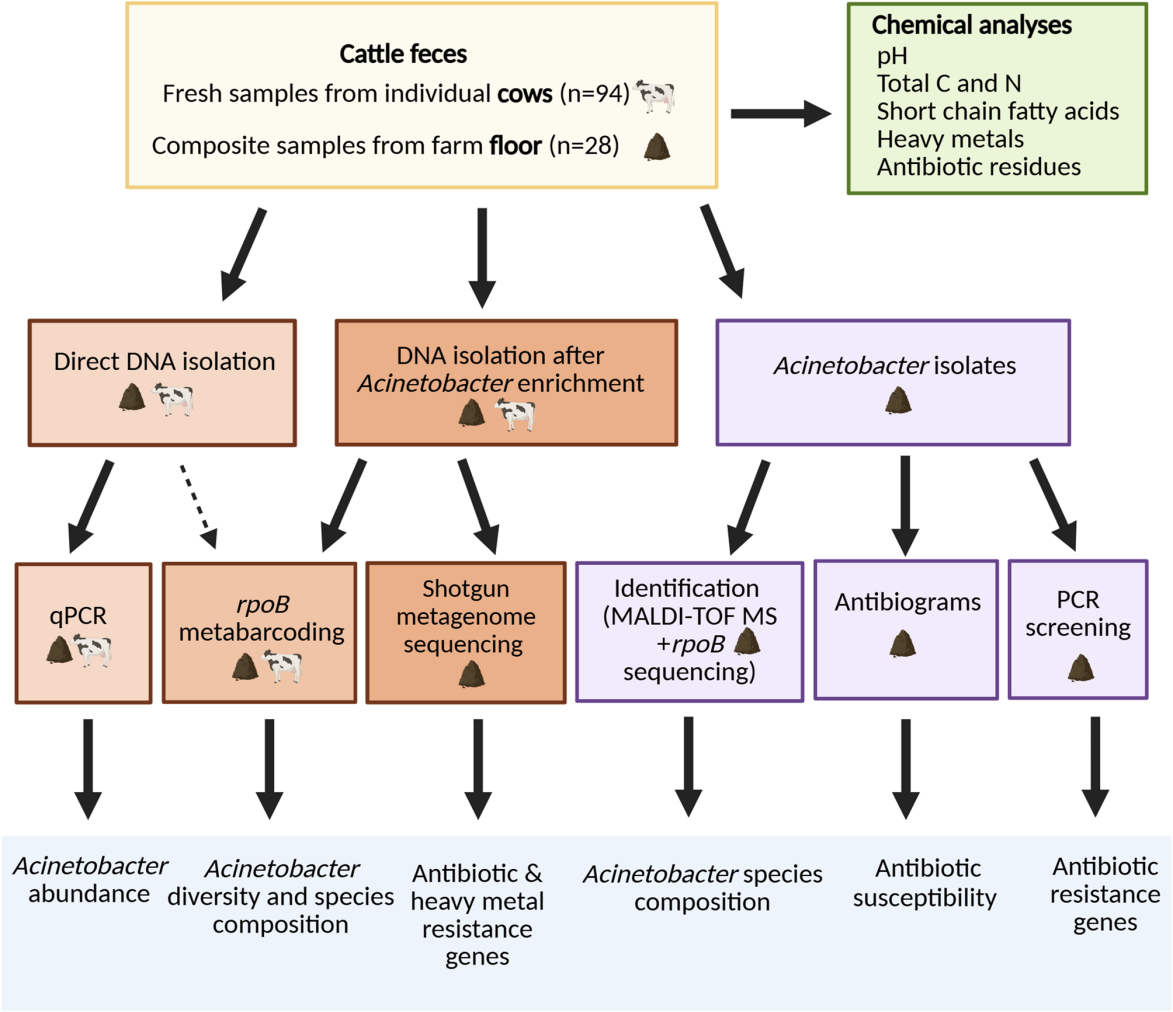
Overview of sample analyses. Visual summary of analyses performed on fecal samples from individual cows (‘cow’ samples) and composite samples from the farm floor (‘floor’ samples) from 28 cattle farms. See Methods for further details. Abbreviations: MALDI-TOF MS, matrix-assisted laser desorption/ionization time-of-flight mass spectrometry; (q)PCR, (quantitative) polymerase chain reaction. Created with BioRender https://BioRender.com/8att3th

Cattle feces were sampled at 28 anonymous Czech cattle farms representing varying levels of antibiotic use between April and October 2022 (Fig. 1 and Table S1). At each farm, a composite sample consisting of 5–10 dung subsamples was collected from the farm floor or pasture (‘floor’ samples, *n* = 28) using a sterile garden trowel while avoiding direct contact with the ground. Additionally, at 14 of these farms, fresh fecal samples were collected from 5 to 11 individual cows per farm (‘cow’ samples, *n* = 93), either directly from the rectum or immediately after defecation, using sterile examination gloves (see Table S2 and Table S3 for sample details). Samples were kept refrigerated at ≈ 4 °C during transport and were processed the same day. Each sample was thoroughly mixed within its collection bag before being divided for subsequent analyses (Fig. 2). Subsamples intended for *Acinetobacter* culturing were prepared first, using sterile spatulas and falcon tubes. These subsamples were either stored directly at 4 °C for use within 1–2 days; mixed with 2 mL of 0.9% NaCl and frozen at −20 °C for use within 6 months; or combined with 2 mL of 0.9% NaCl and 4 mL of glycerol and frozen at −20 °C for long-term storage (>6 months). Subsamples for DNA isolation were stored in sterile Eppendorf tubes at −20 °C. Subsamples for chemical analyses were stored in plastic bags at −80 °C, except for aliquots for dry matter and pH measurements, which were measured upon arrival.

### Chemical composition analysis

Sample dry matter content was determined according to standard protocols [26], and sample pH was measured by directly immersing an electrode and thermometer (Jenway 3510 Standard Digital pH Meter, Cole-Parmer, Vernon Hills, IL, USA) into a sample aliquot [27]. To assess heavy metal content, approximately 0.5 g of the homogenized, lyophilized sample was first mineralized by microwave-assisted acid digestion in a MARS 5 system (CEM Corp., Matthews, NC, USA) using concentrated HNO_3_. The content of metallic elements (Ag, As, Cd, Co, Cr, Cu, Ni, Pb, Sb, Se, Sr, Zn) was determined using inductively coupled plasma optical emission spectrometry (ICP-OES, Agilent Technologies, Santa Clara, CA, USA). The quality of the measurements was monitored by including blank samples, control standards, and replicate measurements. Total C and N content was determined in solid state using a FLASH 2000 CHNS/O elemental analyzer (Thermo Fisher Scientific, Waltham, MA, USA). The content of short-chain fatty acids (SCFA) was determined by Gas Chromatography-Mass Spectrometry (GC–MS) following the optimized protocol described by [28].

The content of antibiotic residues was determined by Liquid Chromatography-Mass Spectrometry (LC–MS) using a custom protocol as follows. Sample extraction was performed using an Accelerated Solvent Extractor (ASE 200; Dionex, Sunnyvale, CA, USA). The extraction cell was filled with approximately 1.5 g (dry weight) of the sample and extracted with methanol. The extract was then concentrated to approximately 10 mL, centrifuged, and 1 mL of the supernatant was transferred to an LC–MS vial for analysis. Targeted analyses were conducted using an Agilent Infinity 1260 liquid chromatograph coupled to an Agilent 6470 triple quadrupole mass spectrometer (LC–TQ; Agilent Technologies, Santa Clara, CA, USA). Chromatographic separation was achieved on a Kinetex Polar C18 (2.6 μm, 3 mm × 100 mm) column equipped with a SecurityGuard Polar C18 (2.6 μm, 3 mm × 2 mm) precolumn (Phenomenex, Torrance, CA, USA), both heated to 40 °C. The mobile phase for gradient elution consisted of phase A: 0.1% formic acid (LC‒MS grade; Honeywell, Charlotte, NC, USA) in Milli-Q water (Smart2Pure™ Water Purification System, Thermo Scientific, Waltham, MA, USA) and phase B: 0.1% formic acid in methanol (LC‒MS grade; Honeywell, Charlotte, NC, USA). The gradient elution program was as follows (time [min]/% phase B): 0/0; 1/0; 4/50; 6/50; 9/95; 10/95; 11/0; 12/0. The flow rate was set to 0.4 mL/min, with a total run time of 15 minutes and an injection volume of 2 µL. The ion source parameters were set as follows: source temperature 180 °C, gas flow 10 L/min, nebulizer 20 psi, sheath gas temperature 300 °C, sheath gas flow 10 L/min, capillary and nozzle voltages 2,500 V and 600 V, respectively. Standard addition was applied to mitigate matrix effects. The mass spectrometer parameters were optimized using MassHunter Workstation Optimizer and Source Optimizer (both Version 10.0, SR1; Agilent Technologies, Santa Clara, CA, USA). A complete list of target analytes and settings is provided in Table S4.

### Strain isolation

*Acinetobacter* strains were isolated from ‘floor’ samples using the selective culture method described by [29]. Samples stored at 4 °C for 1–2 days or mixed with saline and subsequently stored at −20 °C for up to 6 months were used for this purpose. Aliquots containing 2 g of feces were cultured aerobically with vigorous shaking in 25 mL ACE medium (10.0 g KH_2_PO_4_, 5.0 g Na_2_HPO_4_, 2.0 g (NH_4_)_2_SO_4_, 0.2 g MgSO_4_⋅7 H_2_O, 0.001 g CaCl_2_⋅2 H_2_O, and 0.001 g FeSO_4_⋅7 H_2_O per liter, adjusted to pH 7.0, supplemented with 0.5% (w/v) sodium acetate [17]) at 30 and 44 °C for 3 h in parallel. The two temperatures were used to reflect the growth preferences of both environmental and mammal-adapted *Acinetobacter* spp. After 1 h of passive sedimentation, 5 mL of supernatant was transferred to 25 mL of ACE medium and cultured at 30 and 44 °C for up to 2 days. The resulting liquid cultures were then plated onto both ACE agar and CHROMagar™ Acinetobacter (CHROMagar, Paris, France). After 24 h of incubation at 30 and 44 °C, selected colonies were subcultured on sheep blood agar plates (Oxoid, Basingstoke, Hampshire, UK).

### Strain taxonomic analysis

Identification and classification at the species level, as well as dereplication (i.e., the exclusion of multiple isolates of the same strain obtained from a single sample) were performed using combinations of matrix-assisted laser desorption/ionization time-of-flight mass spectrometry (MALDI-TOF MS), sequence analysis of the RNA polymerase β-subunit (*rpoB*) gene, DNA macrorestriction analysis, and additional methods applied to clarify the species status of certain strains (see Table 1).

**Table 1:** Counts and taxonomic assignments of *Acinetobacter* strains. The “Taxonomic assignment” column summarizes the consensus identification of 284 Acinetobacter strains isolated from ‘floor’ samples across 28 cattle farms (see Methods and Table S5 for further details). The “Total strains” column shows the number of strains assigned to the respective taxon, with numbers in parentheses indi­cating the number of strains recovered at 44 °C. The col­umns “F01–F28” indicate the number of strains isolated from individual farms. Note: All *A. baumannii* strains were PCR-positive for the *bla*_oxa-51-like_ gene, confirm­ing their species identity. All *A. vivianii* strains acidified D-glucose and lacked gelatinase activity, differentiating them from the closely related species *A. courvalinii*, *A. dispersus*, *A. gyllenbergii*, *A. modestus*, and *A. proteolyti­cus* [30]

Taxonomic assignment	Total strains (44 °C)	F01	F02	F03	F04	F05	F06	F07	F08	F09	F10	F11	F12	F13	F14	F15	F16	F17	F18	F19	F20	F21	F22	F23	F24	F25	F26	F27	F28
*A. amyesii*	5		1									1										1							2
*A. baumannii*	13 (10)					2			3							2	2	3											1
*A. beijerinckii*	1														1														
*A. faecalis*	13					2					1					2			2		3				1		1	1	
*A. gandensis*	18			3		3		1	2		1		1	1	1	1		1			1	1			1				
*A. guillouiae*	1																								1				
*A. haemolyticus*	1																												1
*A. indicus*	46	3		2			3	4	2	2		1	1	3		1	3	2		2	1	5		3	2	2		2	2
*A. pecorum*	5				3										2														
*A. pseudolwoffii*	32					5	1		3		1		3	1		1		1			2	1	2	1	3	2		4	1
*A. sichuanensis*	1																								1				
*A. thermotolerans*	24 (22)	1				2		4		1				2			3	2		3		3		1				2	
*A. towneri*	1	1																											
*A. variabilis*	12	2		1					1	3						2		1				1						1	
*A. vivianii*	5														2	1	1									1			
*A. wanghuae*	1											1																	
Taxon 7209	10	2		1										1										3				1	2
Taxon 7384	7														3							1	1			1	1		
Taxon 7443	5											3			1								1						
Taxon 7458	3									1												2							
Taxon 7506	12		3												1				3				2				3		
Taxon 7509	9		2								1	1							2				1				2		
Taxon 7517	3											1							2										
Taxon 7576	2																		1								1		
Taxon 7579	4 (2)										2								2										
Taxon 7593	5		2	1							2																		
Taxon 7655	12		1	1							2				2			1	3								2		
Taxon 7683	2								1									1											
Taxon 7947	7 (7)									2												4		1					
*A. sp.* (ungrouped)	7 (2)			2															1							2	1		1
*A. sp.* (*A. lwoffii* phylogroup)	11	1		1	2		1					1			1							1	1			1			1
*A. sp.* (*A. terrae* phylogroup)	1				1																								
*A. sp. (A. schindleri-*like)	2										1			1															
Inconclusive	3				1																	1	1						
Total strains	284 (43)	10	9	12	7	14	5	9	12	9	11	9	5	9	14	10	9	12	16	5	7	21	9	9	9	9	11	11	11

MALDI-TOF MS was performed on a Microflex LT instrument (Bruker Daltonics, Bremen, Germany) with Bruker Biotyper RTC and Compass v4.1.80 software following the standard Bruker protocol [31]. Overnight bacterial cultures were analyzed with α-cyano-4-hydroxycinnamic acid as the matrix. Mass spectra were acquired from at least 40 laser shots at 10 positions in automated mode. Identification was performed using the Bruker reference database (version 2021), supplemented with in-house entries of the type/reference strains of validly named species absent from the Bruker library [32] and the reference strains of novel taxa delineated in the present study. Assignments at the species level were classified according to the Biotyper identification parameters—score values and consistency categories (A or B). “Reliable” was assigned to scores of ≥2.3 (category A) or ≥2.3 (category B) when the second-ranked species differed by ≥0.3, “probable” to scores of 2.0–2.3 (category A) or ≥2.3 (category B) when the second-ranked species differed by ≥0.2, and “possible” to scores of ≥2.0 (category B) when the second-ranked species differed by <0.2. Cluster analysis was performed using UPGMA (unweighted pair group method with arithmetic mean) with correlation-based distance metrics in Compass v4.1.80.

Sequence analysis was performed on a 355-bp fragment of the *rpoB* gene (nucleotide positions 2915–3269 of *Acinetobacter baumannii* CIP 70.34^T^ (GenBank DQ207471.1) [33]. Analyses were carried out in BioNumerics v7.6 (Applied Maths, Sint-Martens-Latem, Belgium) as described previously [34]. The workflow included: (i) compiling a database of *rpoB* sequences from type strains of all validly named *Acinetobacter* species and sequences from this study, (ii) constructing a Neighbor-Joining phylogram from a multiple sequence alignment, and (iii) evaluating percentage identity values. Species-level assignments were defined as ≥97% identity to the closest type strain or reference strain of a tentative novel taxon and categorized as reliable (second-closest species differs by ≥2%), probable (≥1.5%), or possible (<1.5%).

Final identification combined MALDI-TOF MS and *rpoB* results to offset method-specific limitations: MALDI-TOF MS lacks resolution for closely related species, whereas partial *rpoB* sequences may be affected by interspecies recombination. Consensus identification levels (IL) were defined as: IL1, both *rpoB* and MALDI-TOF MS reliable; IL2, *rpoB* reliable and MALDI-TOF MS probable; IL3, *rpoB* probable and MALDI-TOF MS reliable; IL4, both *rpoB* and MALDI-TOF MS probable. All other combinations were interpreted as identification at the genus level only, unless additional taxonomic methods were applied (Table S5).

Additional characterization of *A. baumannii* involved detection of the species-specific *bla*_OXA-51-like_ gene to confirm species identity [35], and multiplex PCR to identify international MDR epidemic clones 1 and 2 [36] prevailing in Czech hospitals. In addition, in-house phenotypic assays were applied to distinguish phylogenetically related species within the *Acinetobacter* hemolytic clade [30].

Dereplication of isolates from each sample was performed in two steps. First, two spectra were obtained per isolate, and all spectra from a sample were clustered with the reference spectra of validly named species and tentative taxa. Clustering at a distance ≤ 50—below which replicate isolates from the same strain consistently clustered—was considered to indicate potential replicate isolates, from which one or two representatives were selected. Second, representatives of the same species were then subjected to macrorestriction analysis [37], and only isolates with distinct macrorestriction patterns were retained in the final set.

### Antibiotic susceptibility testing and screening for antibiotic-resistance genes (ARGs)

Strain susceptibility to antimicrobial agents was determined by the disk diffusion test on Mueller-Hinton agar (Oxoid, Basingstoke, Hampshire, UK) at 30 °C according to standard protocols [38], except for the *Acinetobacter lwoffii* phylogroup (including *A. pseudolwoffii* and Taxon 7443), Taxon 7209, Taxon 7509, Taxon 7947, and Taxon 7579 strains, which displayed poor growth on Mueller-Hinton agar and were tested on Levinthal’s agar medium (HiMedia, Mumbai, India). The antimicrobial agents (Oxoid, Basingstoke, Hampshire, UK; μg/disk) tested were amoxycillin+clavulanate (20+10), ampicillin (10), ampicillin+sulbactam (10+10), cefalotin (30), ceftazidime (30), ciprofloxacin (5), chloramphenicol (30), gentamicin (10), kanamycin (30), meropenem (10), nalidixic acid (30), neomycin (10), penicillin G (10 U), piperacillin (100), streptomycin (10), sulfamethoxazole (25), tetracycline (30), and trimethoprim (5). Minimal inhibitory concentrations (MIC) for colistin were determined using the broth microdilution method [38]. The evaluation of antibiograms aimed to distinguish between wild-type phenotypes and decreased susceptibility due to acquired resistance mechanisms. To achieve this, we visually examined the distribution of inhibition zone diameters for each antibiotic and species/taxa with at least 10 strains to identify deviations from a normal distribution, which could suggest the presence of acquired resistance mechanisms [39]. Ten species/taxa were examined in this way, whereas a group of 11 strains from the *A. lwoffii* phylogroup, which could not be identified at the species level, was excluded due to their potential species-level heterogeneity. In addition, *A. baumannii* strains were tested by the disk diffusion test against clinically relevant antibiotics primarily effective against this species: amikacin (30), doxycycline (30), imipenem (10), netilmicin (30), ofloxacin (5), piperacillin+tazobactam (100+10), trimethoprim+sulfamethoxazole, (1.25+23.75), and tobramycin (10). *A. baumannii* strains were classified as susceptible, intermediate, or resistant according to the recommendations of the Clinical and Laboratory Standards Institute [38]. *A. pseudolwoffii* strains ANC 7479, ANC 7490, and ANC 7493 were additionally analyzed for imipenem MIC using E-test (bioMérieux, Marcy-l’Étoile, France).

*Acinetobacter* strains displaying non-wild-type decreased susceptibility to streptomycin, tetracycline, and sulfamethoxazole were further screened for the presence of the corresponding acquired ARGs. PCR screening of crude cell lysates targeted the following ARGs: *strA*, *strB*, and *aadA27* (streptomycin resistance), *tet*(Y) (tetracycline resistance), as well as *sul1* and *sul2* (sulfamethoxazole resistance). All strains were additionally screened for the presence of the carbapenemase gene *bla*_OXA-58_ and the replication initiation gene *V216rep* associated with LowGC-type plasmids [18]. PCR primers and conditions are described in Table S6. The specificity of PCR products was verified by Sanger sequencing, except for *strA* and *strB*, when they were positive in both separate amplification and co-amplification (*strA–strB* fragment).

### DNA isolation from cattle feces

Total fecal DNA was extracted from 150 mg of each fecal sample (*n* = 121) using the DNeasy PowerSoil Pro Kit (Qiagen, Hilden, Germany), with the following modification in the bead-beating step to enhance DNA yield. The bead-beating tubes containing the sample suspension were subjected to two cycles of shaking in a FastPrep-24™ 5 G instrument (MP Biomedicals, Santa Ana, CA, USA) located in a cold room (8 °C), with each cycle lasting 30 s at a speed of 6 m/s and separated by a 30-second pause. This was followed by an incubation step at 65 °C for 5 minutes, after which the tubes were shaken and incubated again under the same conditions.

### *Acinetobacter* quantification

To assess *Acinetobacter* abundance in cattle feces, qPCR was performed on the total fecal DNA using *Acinetobacter* genus-specific primers Ac436f/Ac676r (targeting the 16S rRNA gene [40]). The qPCR reactions were prepared in duplicate, in a total volume of 20 µL, containing 10 µL of SsoFast EvaGreen Supermix (Bio-Rad, Hercules, CA, USA), 2 µL of template DNA (diluted to 2–5 ng/µL), and 0.2 µM of each primer [24]. The thermocycling conditions were as follows: 95 °C for 10 min, followed by 40 cycles of 95 °C for 15 s and 60 °C for 20 s (using the qTOWER^3^ touch, Analytik Jena, Jena, Germany). Finally, a melting curve was generated by analyzing the qPCR products in a temperature gradient from 60 to 95 °C. In parallel, total bacteria were quantified using universal 16S rRNA primers 1108f/1132 r [41]. The reaction conditions were the same as above except for a final primer concentration of 0.3 µM. The thermocycling conditions were set as follows: 95 °C for 10 min, 40 cycles of 95 °C for 15 s, 52.5 °C for 35 s, and 72 °C for 10 s. A standard for quantification of both *Acinetobacter* and total bacteria was prepared by amplification of the 16S rRNA gene from *A. baumannii* NIPH 501^T^ using the primers 27F/1492 R [42]. The standard curve was generated by plotting Ct values against a 10-fold standard dilution series ranging from 10^9^ to 10^2^ gene copies in four replicates. The specificity of qPCR products was confirmed through melting curve analysis and product size verification after DNA electrophoresis.

To assess the limits of detection (LOD) and quantification (LOQ) for *Acinetobacter* qPCR, two-fold dilutions of standards with gene copies ranging from ≈10^3^ to < 1 were prepared in six replicates. The LOD and LOQ were calculated using a curve-fitting model implemented in an R script provided by Klymus et al. [43]. The 95% LOD was determined to be 7.7 gene copies per reaction, while the LOQ, defined by a 35% coefficient of variation threshold, was 68 gene copies per reaction. All values < LOD were replaced by the LOD, while values < LOQ (but >LOD) were replaced by the LOQ. Finally, the absolute *Acinetobacter* abundance was expressed as the number of *Acinetobacter* 16S rRNA gene copies per g of dry weight feces, while the relative abundance was calculated as the ratio of *Acinetobacter*/total bacteria 16S rRNA gene copies.

### DNA isolation from enrichment cultures

Due to the insufficient abundance of acinetobacters in most fecal samples for downstream DNA-based analyses, we established enrichment cultures from all 121 fecal samples. The enrichments were done in liquid ACE medium ([17, 29], see above), using 2 g of each sample (stored in 2 mL of 0.9% saline plus 4 mL of glycerol at −20°C). Briefly, samples were washed twice with 0.9% saline and incubated in 25 mL of ACE medium at 30 °C with shaking at 160 rpm for 3 h, followed by passive sedimentation for 30 min. Subsequently, 5 mL of the supernatant was transferred to a 100-mL Erlenmeyer flask containing 25 mL of fresh ACE medium and incubated at 30 °C with shaking at 160 rpm for 2 days. DNA was extracted from the grown cultures using the DNeasy UltraClean Microbial Kit (Qiagen, Hilden, Germany).

### Diversity and species composition assessment using *rpoB* metabarcoding

To study the diversity of *Acinetobacter* spp. in cattle feces, metabarcoding of a 355-bp variable region of the *rpoB* gene was performed using either total fecal or enrichment DNA as templates (see above). The variable region was amplified with *Acinetobacter*-specific primers Ac696F and Ac1093R [33], containing custom-designed barcodes (Table S7). PCR was performed using a T100 PCR thermocycler (Bio-Rad, Hercules, CA, USA) in a total reaction volume of 25 µL, containing 1× Q5 Reaction Buffer, 200 µM deoxynucleoside triphosphates, 0.2 µM of each primer, 0.02 U/µL Q5 High-Fidelity DNA Polymerase (New England Biolabs, Ipswich, MA, USA), 0.6 µg/µL bovine serum albumin, 1× Q5 High GC Enhancer, and 10–20 ng of template DNA. The PCR thermocycling conditions were as follows: an initial denaturation at 98 °C for 2 min, followed by 35 cycles of 98 °C for 30 s, 52 °C for 30 s, and 72 °C for 30 s, with a final extension at 72 °C for 2 min. The amplified PCR products were purified using the MinElute PCR Purification kit (Qiagen, Hilden, Germany) and their concentration was measured using a Qubit 2.0 fluorometer (Thermo Scientific, Waltham, MA, USA). Subsequently, sequencing libraries were constructed using the TruSeq DNA PCR-free kit (Illumina, San Diego, CA, USA) and sequencing was performed in-house using Illumina MiSeq (2 × 250 bp paired-end reads; Illumina, San Diego, CA, USA). Using total fecal DNA as a template, successful amplification and sequencing of the *rpoB* gene were achieved for only 21 out of 121 samples, while 118 samples were successfully amplified and sequenced based on enrichment DNA.

The sequencing data from Illumina MiSeq were processed with SEED v2.1.2 [44]. The paired-end reads were first joined using fastq-join [45]. Sequences with an average quality score below 30 or a length exceeding 394 bp were discarded. Following primer removal, chimeric sequences were identified and eliminated using VSEARCH v2.15.0 [46]. The remaining high-quality, non-chimeric sequences were clustered at 98% similarity with VSEARCH, and the most abundant sequence within each cluster was selected as a cluster-representative sequence.

Assignment of *rpoB* clusters to *Acinetobacter* species involved two steps. First, each representative sequence was taxonomically assigned at the genus level using BLASTn v2.5.0 [47] against a comprehensive *rpoB* database (FROGS rpoB_122017.fasta containing 44,673 bacterial entries [48]). Only sequences (clusters) whose best hit corresponded to the genus *Acinetobacter* with a minimum of 95% coverage were retained. As a result, 4% of the clusters were removed from the dataset due to non-*Acinetobacter* affiliations (mainly *Psychrobacter*). In the second step, species-level identification of the *Acinetobacter*-specific *rpoB* clusters was performed using BLASTn against our custom reference database containing 178 *Acinetobacter rpoB* sequences (Table S8 and Additional file 1), using ≥ 95% coverage and ≥97% identity thresholds.

### Resistome and mobilome analysis via shotgun metagenome sequencing of enrichment cultures

To study *Acinetobacter* antibiotic and heavy metal resistance genes and their genetic context, shotgun metagenome sequencing of *Acinetobacter* enrichment DNA from 28 ‘floor’ samples was performed using the combination of Illumina and Oxford Nanopore platforms. Illumina library preparation (Illumina® DNA Prep, Illumina, San Diego, CA, USA) and sequencing (NovaSeq 6000 System − 2 × 150 bp; Illumina, San Diego, CA, USA) were done at SEQme Ltd. (Dobříš, Czech Republic). Illumina reads were quality-controlled using fastp [49] and initially assembled with Megahit v1.2.9 [50]. The share of *Acinetobacter* reads in each sample was then estimated as the proportion of reads mapped to *Acinetobacter*-specific versus total *rpoB* gene sequences. This was determined by BLASTn of predicted genes (FragGeneScan v1.31 [51]); against the FROGS *rpoB* database (Table S9). Oxford Nanopore DNA libraries were prepared with the Native Barcoding kit 24 V14 (Oxford Nanopore Technologies, Oxford, UK) and sequenced on two R10.4.1 flow cells using the Oxford Nanopore PromethION 2 Solo platform (Oxford Nanopore Technologies, Oxford, UK). The pooling of Oxford Nanopore sequencing libraries was done based on the anticipated share of *Acinetobacter* sequences in each sample (Table S9), with the aim of obtaining comparable numbers of *Acinetobacter* long-read sequences across samples. Basecalling using the SUPv4.3 (super accurate) algorithm and demultiplexing of the Nanopore reads was done by Dorado v0.5.3 (Oxford Nanopore Technologies, Oxford, UK). Extra adapter removal, quality control (reads filtered at average read quality Q > 15 and length > 1000 b) and removal of lambda DNA was done with duplex-tools (https://github.com/nanoporetech/duplex-tools) and chopper [52].

The final sequence assembly was performed on a per-sample basis using Flye v2.9.3 [53] for chromosome assembly and Plassembler v1.6.2 [54] for plasmid assembly. The Flye and Plassembler modes yielding optimal results are indicated in Table S10. Binning was intentionally not performed because high numbers of closely related *Acinetobacter* strains/species in the enrichment cultures would likely result in chimeric bins.

Taxonomic classification of contigs was done with GTDB-Tk v2.1.0 [55] based on the taxonomy R207_v2 from the Genome Taxonomy Database (GTDB). Contigs that remained unidentified with GTDB-Tk (contigs with no or low numbers of marker genes) were further classified to the lowest common ancestor with CAT [56], using the NCBI nr database (accessed 2024-Apr) and parameters *r* = 1 and *f* = 0.6. Finally, we aimed to achieve accurate species-level classification for contigs exceeding 250,000 bases that were assigned to the *Acinetobacter* genus based on GTDB-Tk or CAT results. To accomplish this, we calculated their average nucleotide identity to *Acinetobacter* reference genomes using the ANIb method implemented in PYANI v0.2.10 (https://pypi.org/project/pyani [57]).

Two complementary approaches were employed to identify plasmid contigs. First, Plassembler searches each putative plasmid contig against the PLSDB plasmid database ([58]; 34,513 entries) and retains matches with a Mash distance < 0.1. Second, contigs carrying replication initiation (*rep*) genes characteristic of *Acinetobacter* plasmids were identified by querying predicted coding sequences against the Acinetobacter Plasmid Typing database ([59]; 1,846 entries) using BLASTn v2.5.0 [47]. Hits were retained if the alignment length exceeded 800 bp (given that the smallest *rep* genes are ≈850 bp) and coverage was > 95%, and were assigned to known *Acinetobacter* plasmid Rep types based on a 95% identity threshold [59].

The presence of ARGs in assembled metagenomic data was predicted using Abricate v1.0.1 (https://github.com/tseemann/abricate) with the NCBI AMRFinderPlus data-base ([60]; accessed 2023-Nov-04; 5,386 entries). Contigs carrying ARGs that could not be taxonomically classified as described above were further examined using NCBI BLASTn [47] against the core_nt database (https://blast.ncbi.nlm.nih.gov/Blast.cgi; accessed 2025-Jun-06). Contigs showing ≥ 99% identity and ≥99% coverage with *Acinetobacter* sequences were retained for downstream analyses along with those assigned to *Acinetobacter* in the earlier step. Genetic context of the identified ARGs was determined using nucleotide/protein BLAST [47], RAST [61], and ISfinder [62], and visualized in SnapGene v8.1.1 [63].

To identify HMRGs, coding sequences were predicted by FragGeneScan v1.31 [51] and queried against the MetalResistance database (https://orbit.dtu.dk/en/datasets/metalresistance-collection-of-metal-resistance-genes; 578 entries [64]); using tBLASTx [47]. Hits with the alignment length > 50 amino acids (approximate size of the smallest HMRG), coverage > 70%, and amino acid identity > 60% were retained.

### *A. pseudolwoffii* ANC 7493 genome sequencing and analysis

To confirm the presence of *bla*_OXA-58_ in *A. pseudolwoffii*, strain ANC 7493 was chosen for genome sequencing. The genomic DNA was extracted from an overnight culture grown on Nutrient Agar (Thermo Fisher Scientific, Waltham, MA, USA), using the DNeasy UltraClean Microbial Kit (Qiagen, Hilden, Germany). A DNA library was then prepared with the Oxford Nanopore Native Barcoding Kit 24 V14 (Oxford Nanopore Technologies, Oxford, UK) and sequenced on an R10.4.1 flow cell using the Oxford Nanopore PromethION 2 Solo platform (Oxford Nanopore Technologies, Oxford, UK). Basecalling and raw sequence processing were done as described above, but reads were quality-filtered at Q > 20. The genomic sequence was assembled with the Hybracter pipeline [65] and annotated with RAST [61]. The presence of ARGs was confirmed with Abricate as described above. The plasmid pANC7493.1 was further annotated using Prokka [66], ISfinder [62], and nucleotide and protein NCBI BLAST [47], and the annotated sequence was visualized with Proksee [67]. Alignments between pANC7493.1 and contig F17_297 were done in MEGA [68] using ClustalW [69] and visualized with gggenomes (https://github.com/thackl/gggenomes [70]).

### Statistical analysis

All statistical analyses were conducted using R version 4.5.1 [71] in RStudio version 2025.5.1.513 [72] at *p* < 0.05. Plotting was done using the ggplot2 package [73].

#### Abundance analysis

The differences in *Acinetobacter* abundance between ‘floor’ samples and the per-farm mean of ‘cow’ samples were tested using the Wilcoxon signed-rank test. Further, *Acinetobacter* abundance was compared between dairy and beef farms using the Wilcoxon rank-sum test and among farms with different stabling types (outdoor, indoor, indoor/outdoor) using the Kruskal-Wallis rank-sum test. Correlations between *Acinetobacter* abundance and factors such as per-farm antibiotic use, herd size, age of individual cows, sample dry-matter content (%), sample pH, sampling temperature, heavy-metal content, total C and N levels, presence of antibiotic residues, and total SCFA were tested using Spearman’s rank correlation, and the *p*-values were adjusted using the Benjamini–Hochberg (BH) method.

#### Species diversity analysis

*Acinetobacter* diversity based on *rpoB* metabarcoding data (Additional file 2) was analyzed using the Phyloseq [74], metagMisc [75] and Vegan [76] packages. To reduce potential biases in the relative abundance of *Acinetobacter rpoB* clusters arising from differences in strain growth rates in the enrichment cultures (see above), all relative abundance datasets were converted to presence–absence data before performing alpha- and beta-diversity analyses. Observed richness was selected as a measure of alpha diversity because it is independent of relative abundance. To calculate Observed richness, we used the phyloseq_mult_raref_div function to randomly rarefy the *Acinetobacter* sequence counts per sample 100 times to a sequencing depth of 6,702 while excluding two ‘cow’ samples that were below this depth. The average observed richness was then statistically compared between ‘floor’ and the per-farm mean of ‘cow’ samples, between dairy and beef farms, among farms with different stabling types and across farms with varied antibiotic use. In the case of ‘floor’ samples, the statistical tests performed were the same as those applied to compare *Acinetobacter* abundance across groups (see above). In the case of ‘cow’ samples, linear mixed-effects modeling was used with the compared groups as fixed effects and the farm as a random effect, and the significance of the effects was tested using *F*-tests with ANOVA.

Prior to beta-diversity analyses, multiple rarefactions were performed using the phyloseq_mult_raref_avg function under the same conditions as above. *Acinetobacter* communities (based on *rpoB* clusters) were compared across samples using non-metric multidimensional scaling (NMDS) with a Sørensen dissimilarity matrix. Initially, we aimed to evaluate the dissimilarity in *Acinetobacter* communities between individual ‘cow’ samples and corresponding ‘floor’ samples from the same farm. To do this, we conducted NMDS using data from the 14 farms where both sample types were available. Based on the NMDS ordination, z-scores were calculated for the ‘floor’ samples to quantify their dissimilarity relative to the individual ‘cow’ samples from the same farm. The z-scores (z-score =(d_F_ - d_c_) / σ_c_) were computed using the mean (d_c_) and standard deviation (σ_c_) of the Euclidean distances of individual ‘cow’ samples to their farm-specific centroid, and the Euclidean distance of the ‘floor’ sample (d_F_) to the corresponding farm-specific centroid. Further, separate NMDS ordinations were performed for ‘floor’ and ‘cow’ samples, onto which environmental variables (farm and cow-specific variables) were fitted using the envfit function.

To partition the variance in *Acinetobacter* species distributions among the farm- and cow-specific variables as well as to test the effect of those variables on *Acinetobacter* species, hierarchical modeling of species communities (HMSC [77]); was used. HMSC is a multivariate hierarchical generalized linear model that uses Bayesian inference. For HMSC analysis, *Acinetobacter rpoB* clusters were first grouped at the species level (Additional file 3–4) and only those species present in at least five ‘floor’ or ‘cow’ samples were included in the models. The response matrix Y consisted of presence-absence data for *Acinetobacter* species and a binomial model with a probit link was fitted to each species. The HMSC models were built separately for ‘floor’ and ‘cow’ samples and the selection or transformation of explanatory variables for each subset was done to optimize the species versus explanatory variable numbers, eliminate inter-correlated variables and minimize categories with a low number of observations. Notably, ‘indoor/outdoor’ category for stabling was grouped with ‘outdoor’, since cows from ‘indoor/outdoor’ stabling stay outdoors most of the year. In addition, the highly intercorrelated heavy metal content data were reduced into two principal components (PCs, Fig. S1, Table S11), and antibiotic content data, containing many zero values, were considered as simple presence or absence of antibiotic residues in the samples. The X matrix of explanatory variables of the HMSC model for ‘floor’ samples thus included the following variables that varied between farms: production (dairy, beef), stabling (outdoor, indoor), herd size, log-transformed per-head antibiotic use, sampling temperature, and total SCFA. The X matrix of the HMSC model for ‘cow’ samples included the above-mentioned variables (except total SCFA) as well as the following variables that varied between cows within farms: age of individual cows, sample pH, C/N ratio, total SCFA, presence of antibiotic residues, and PC1 and PC2 for heavy metal content. To account for differences in sequencing depth, the log-transformed number of reads was also included as an explanatory variable for both models. Finally, the ‘floor’ model used a farm-level random effect whereas the ‘cow’ model used farm-level and cow-level random effects. The models were fitted assuming default priors and sampled the posterior distribution by running four Markov Chain Monte Carlo (MCMC) chains, each of which was run for 3,750 iterations with 1,250 discarded as burn-in. We thinned by 10 to obtain a total of 250 posterior samples per chain and 1,000 total posterior samples. To test for MCMC convergence we measured the potential scale reduction factor for the beta (capturing species responses to explanatory variables) parameters and assumed satisfactory convergence when they were close to one. The R scripts used for HMSC analysis are available at the Zenodo repository (https://zenodo.org/), doi: 10.5281/zenodo.17426203.

#### Antibiotic susceptibility and antibiotic and heavy metal resistance gene analysis

Statistical analysis of antibiotic susceptibility data was performed for species with a sufficient number of strains – specifically, *A. pseudolwoffii* (*n* = 32) and *A. indicus* (*n* = 45; one strain with *rpoB* sequence identity < 97% to the type strain was excluded). Analyses were restricted to antibiotics for which these species showed non-wild-type decreased susceptibility (i.e., sulfamethoxazole, cefalotin, and streptomycin in both species, and penicillin in *A. pseudolwoffii*). Inhibition zone diameters were compared between strains isolated from antibiotic-using and antibiotic-free farms using the Wilcoxon rank-sum test, or across on-farm antibiotic use categories using the Kruskal–Wallis test, and the *p*-values were adjusted for multiple testing using the Benjamini-Hochberg (BH) method. The richness of acquired ARGs or HMRGs in the *Acinetobacter* enrichment metagenomes was compared between antibiotic-using and antibiotic-free farms with the Wilcoxon rank-sum test. Comparisons were performed for both the raw counts of unique ARG or HMRG types and for counts normalized by assembly length (log-transformed).

## Results

### Farm and sample description

This study included 28 Czech cattle farms sampled from spring to autumn 2022, aiming to encompass diverse farm settings typical of Czechia. The farms thus varied in their production type (17 dairy and 11 beef farms), herd size (ranging from 5 to 1,250 cows), and stabling system (indoor, outdoor, or a combination with indoor overwintering) (Fig. 1 and Table S1). According to farmers’ reports, 11 farms had not administered any antibiotics to their cattle in the six months prior to sampling. Of the remaining 17 farms, four used less than 100 g of antibiotics in total, eight used several hundred grams, and five used quantities in the thousands of grams (Fig. 1). The types of antibiotics used varied considerably between farms (Table S1). The most commonly reported antibiotics were procaine benzylpenicillin, amoxicillin (both β-lactams), and dihydrostreptomycin (aminoglycoside) (Fig. 1 and Fig. S2). Antibiotic use was primarily associated with dairy cattle, largely due to frequent treatment of mastitis. Dairy herds are also more likely to be housed indoors than beef cattle. This imbalance in the dataset reflects the practical realities of cattle farming in Czechia. Sampling included composite fecal samples collected from farm floors or pastures (one per farm, referred to as ‘floor’ samples, Table S2), as well as samples from individual cows with known age, breed, and recent antibiotic history, collected on 14 farms (referred to as ‘cow’ samples, *n* = 93, Table S3). Thus, 121 samples were obtained in total.

### Antibiotic residues and heavy metals are present in cattle feces

Antibiotic residues were sporadically detected in cattle feces (Table S2 and Table S3), even on farms reporting high antibiotic usage. The detected antibiotics included β-lactams (procaine benzylpenicillin and penicillin G), fluoroquinolones (marbofloxacin and enrofloxacin), aminoglycosides (dihydrostreptomycin and novobiocin), tetracyclines (oxytetracycline), lincosamides (lincomycin), and rifamycins (rifaximin). These antibiotics generally reflected those reportedly used on the farms. However, the β-lactams amoxicillin and ceftiofur were not detected in any sample, despite their frequent application. Penicillin G was occasionally detected in low quantities on farms or in cows with no recent record of antibiotic treatment.

Heavy metals were present in all samples (Table S2 and Table S3). The detected heavy metals were arsenic (As), cadmium (Cd), cobalt (Co), chromium (Cr), copper (Cu), nickel (Ni), lead (Pb), antimony (Sb), selenium (Se), strontium (Sr), and zinc (Zn). Among them, Cu, Zn, and Sr were detected at the highest levels (dozens to hundreds of µg/g), and Cu and Zn levels were positively correlated with on-farm antibiotic use (Rho = 0.6) (Fig. S3).

### *Acinetobacter* strains from cattle feces are taxonomically diverse and include putative novel species

*Acinetobacter* isolates were recovered from all 28 ‘floor’ fecal samples included in the study. Approximately 800 isolates were analyzed in total, and dereplication resulted in 284 distinct strains. Table 1 summarizes their consensus identification/classification and distribution across samples. Detailed results are provided in Table S5, and a genus-wide *rpoB* phylogram is shown in Fig. S4.

A total of 179 strains (63%) were assigned to 16 validly named species. The most frequently recovered species were *A. indicus* (46 strains from 20 ‘floor’ samples), *Acinetobacter pseudolwoffii* (32 from 16), *Acinetobacter thermotolerans* (24 from 11), *Acinetobacter gandensis* (18 from 13), *A*. *baumannii* (13 from 6; none of the strains belonged to international clone 1 or 2), *Acinetobacter faecalis* (13 from 8), and *A. variabilis* (12 from 8). *Acinetobacter amyesii*, *Acinetobacter pecorum*, and *Acinetobacter vivianii* were each represented by five strains, while six additional species were recovered only once (Table 1).

Eighty-one strains (28.5%) were classified into 13 novel taxa, i.e., taxonomically unique groups each comprising at least two strains from different samples with distinct macro-restriction profiles. These taxa most likely represent novel species, as supported by their unique, nearly homogeneous MALDI-TOF MS and *rpoB* profiles, which reliably separate them from known species and from each other (Fig. S4, Table S5). Each taxon was designated by the strain number of the reference isolate used for MALDI-TOF MS and *rpoB* analysis (Table S5). For example, Taxon 7509 was designated by the reference strain ANC 7509. The most frequent taxa were Taxon 7506 (12 strains from 5 samples), Taxon 7655 (12 from 7), Taxon 7209 (10 from 6), Taxon 7509 (9 from 6), Taxon 7384 (7 from 5), and Taxon 7947 (7 from 3) (Table 1).

The remaining 24 strains (8.5%) could not be assigned or classified at the species level. Based on their unique profiles, seven strains most likely represent six additional novel species (including two highly similar strains from a single sample). Eleven strains belonged to *A. lwoffii* phylogroup, i.e., a phylogenetic lineage encompassing *A. lwoffii*, *A. pseudolwoffii*, *A. pecorum*, and Taxon 7443; however, the available data did not permit conclusive identification or classification. Similar situations were observed for one strain within the phylogroup typified by *Acinetobacter terrae* [78] and for two strains related to *Acinetobacter schindleri*. Finally, conflicting MALDI-TOF MS and *rpoB* sequencing results for the last three strains precluded any taxonomic conclusion (Table 1).

Selective isolation at 30 °C yielded 241 strains representing all identified species, taxa, and other taxonomic types, except for Taxon 7947 and two taxonomically unique strains. In contrast, selective isolation at 44 °C recovered only 43 strains, belonging to *A. baumannii*, *A. thermotolerans*, two novel taxa, and two taxonomically unique strains.

### *Acinetobacter* strains display decreased susceptibility to multiple antibiotics

**Fig. 3:**
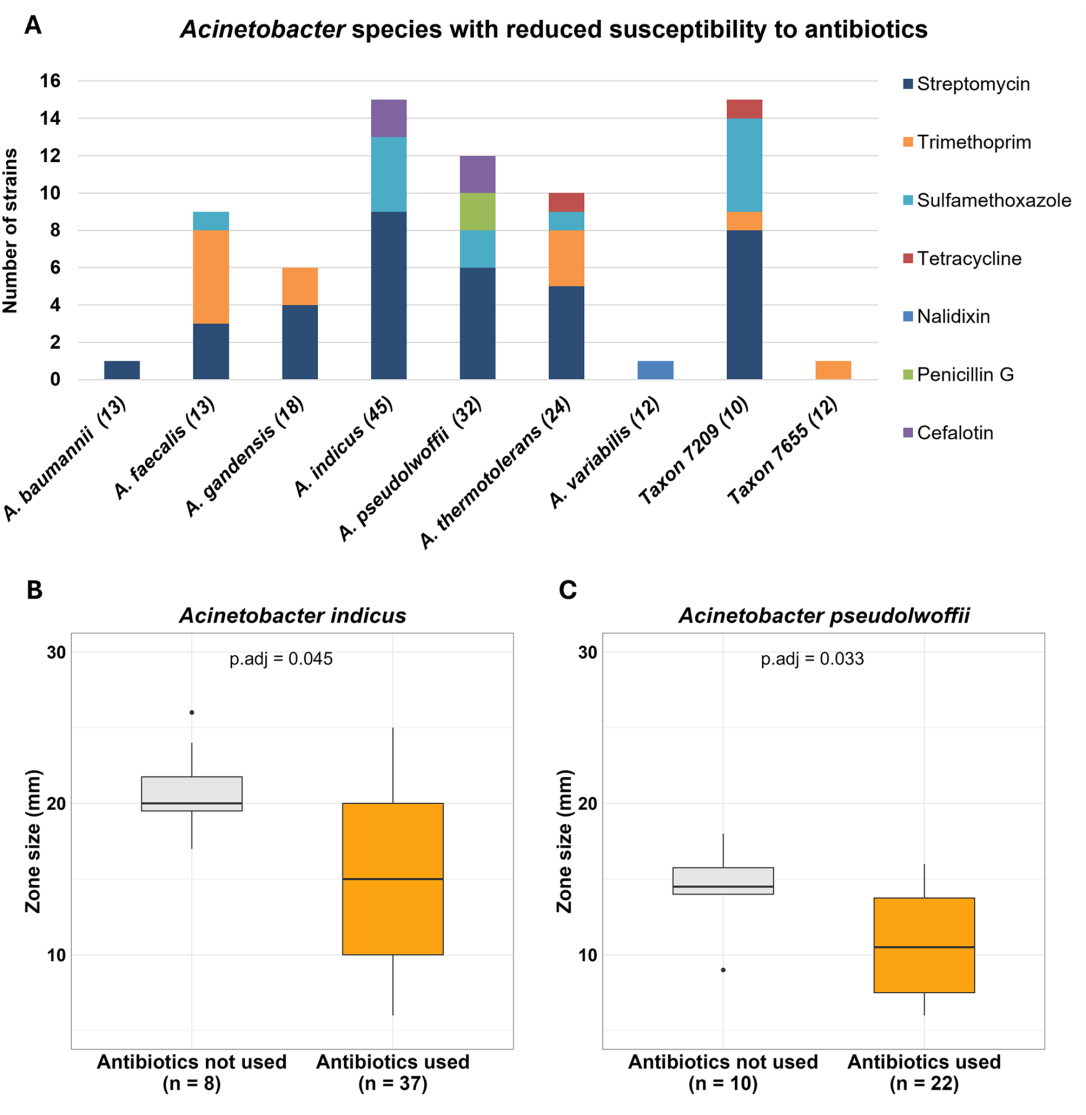
Antibiotic susceptibility in *Acinetobacter* strains. (**A**) Numbers of strains with reduced susceptibility to antibiotics per *Acinetobacter* species. Only species comprising ≥ 10 strains were analyzed and the total number of strains per species is indicated in parentheses after the species name (note that individual strains may show reduced susceptibility to multiple antibiotics). (**B**) and (**C**) Analysis of streptomycin susceptibility in strains of *A. indicus* (**B**) and *A. pseudolwoffii* (**C**). Streptomycin inhibition zone size was compared between isolates from antibiotic-using (“antibiotics used”) and antibiotic-free (“antibiotics not used”) farms using Wilcoxon rank-sum test (p.adj is Benjamini–Hochberg-corrected *p*-value). Boxes represent the interquartile range (Q1– Q3), with median drawn as a horizontal line. Whiskers indicate the smallest and largest values within 1.5 times the interquartile range. Outliers are drawn as points

In the disk diffusion test using a panel of 18 antibiotics, some *Acinetobacter* strains displayed no inhibition zones when tested with penicillin (*n* = 27), ampicillin (*n* = 15), cefalotin (*n* = 31), streptomycin (*n* = 28), tetracycline (*n* = 2), sulfamethoxazole (*n* = 19), trimethoprim (*n* = 45), or chloramphenicol (*n* = 9), indicating non-susceptibility to these antibiotics (Table S5). However, interpreting these results requires consideration of the species context to differentiate between intrinsic and acquired resistance. For instance, *A. baumannii* is intrinsically resistant to penicillin, ampicillin, cefalotin, and chloramphenicol. Therefore, we further examined the inhibition zone size distribution within species/taxa with sufficient strain numbers, where small zones deviating from normal distribution indicate the presence of acquired resistance mechanisms (Fig. S5–14). This analysis indicated the presence of acquired resistance to cefalotin (*n* = 4 strains), nalidixic acid (*n* = 1), penicillin G (*n* = 2), streptomycin (*n* = 36), sulfamethoxazole (*n* = 13), tetracycline (*n* = 2), and trimethoprim (*n* = 12) across nine *Acinetobacter* species/taxa (Fig. 3A). As several strains showed acquired resistance to more than one antibiotic, the total number of strains with acquired resistance to at least one antibiotic was 57. Notably, three MDR *Acinetobacter* strains (i.e. displaying acquired non-susceptibility to at least one agent in three or more antimicrobial categories [79]) were identified within *A. faecalis* (strain ANC 7486), *A. thermotolerans* (ANC 7955), and Taxon 7209 (ANC 7562). All three strains were isolated from farms that had used several hundred grams of antibiotics within the previous six months.

In contrast, all *A. baumannii* strains were wild-type susceptible to all tested antibiotics except one strain, which showed reduced susceptibility to streptomycin. Furthermore, *A. baumannii* strains were susceptible to an extended panel of clinically relevant antibiotics (Fig. S14). The colistin MIC values of all 284 strains remained ≤ 2 mg L^−1^, suggesting an overall good susceptibility.

Detailed analysis of streptomycin inhibition zone diameters in *A. indicus* and *A. pseudolwoffii* (the species with the highest number of strains) revealed a significant reduction in streptomycin susceptibility among strains from antibiotic-using farms compared with those from antibiotic-free farms (Fig. 3BC). Moreover, susceptibility differed across the categories of on-farm antibiotic use, with the lowest median inhibition zones observed in isolates from high-antibiotic-use farms (Fig. S15).

### Acquired antibiotic resistance genes and LowGC-type plasmids are present in *Acinetobacter* strains

PCR screening for acquired ARGs initially focused on those frequently found in European farm environments, as well as genes previously identified in a subset of strains with available whole genome sequences obtained in this project [32, 80]. These included *strA*, *strB*, and *aadA27* (streptomycin resistance), *tet*(Y) (tetracycline resistance), and *sul1* and *sul2* (sulfamethoxazole resistance) (Table S5). Of the 36 strains with reduced susceptibility to streptomycin, 28 carried both *strA* and *strB*, while five carried *aadA27*. Both strains with reduced susceptibility to tetracycline harbored *tet*(Y). Eight strains with reduced susceptibility to sulfamethoxazole (out of 13) carried *sul2*, while none was *sul1*-positive.

Screening was further extended to include strains with small inhibition zones (≤12 mm), where reduced susceptibility could not be clearly attributed to intrinsic or acquired mechanisms due to low numbers of strains per species/taxon. This notably raised the number of *strA-strB*-positive strains to 53 (out of 65 examined), spanning 10 species or novel taxa, suggesting their wide distribution (Table S5). In addition, these genes occurred in combination with *sul2* and/or *tet*(Y) in 11 strains. Notably, two strains, ANC 7562 and ANC 7968, belonging to the novel Taxon 7209 and Taxon 7947, respectively, carried *strA-strB*, *tet*(Y), and *sul2.*

All strains were additionally screened for the presence of the carbapenemase gene *bla*_OXA-58_, which was detected in the *Acinetobacter* enrichment cultures (see below). This screening revealed the presence of *bla*_OXA-58_ in three strains of *A. pseudolwoffii*, i.e. ANC 7479, ANC 7490, and ANC 7493. These strains were susceptible to meropenem (Table S5) and imipenem (MIC values 0.125–0.5 mg/L) but had decreased susceptibility to streptomycin and harbored *strA* and *strB*.

Finally, PCR screening targeted *Acinetobacter*-specific LowGC-type plasmids, known to mediate ARG transfer in livestock settings [18]. Three strains belonging to *A. pecorum* (ANC 7879), *A. variabilis* (ANC 7728), and the *A. lwoffii* phylogroup (ANC 7878) were positive and all showed large inhibition zones for all tested antibiotics except trimethoprim.

### *Acinetobacter* abundance in cattle feces is low, but increases in feces deposited on farm floor

**Fig. 4:**
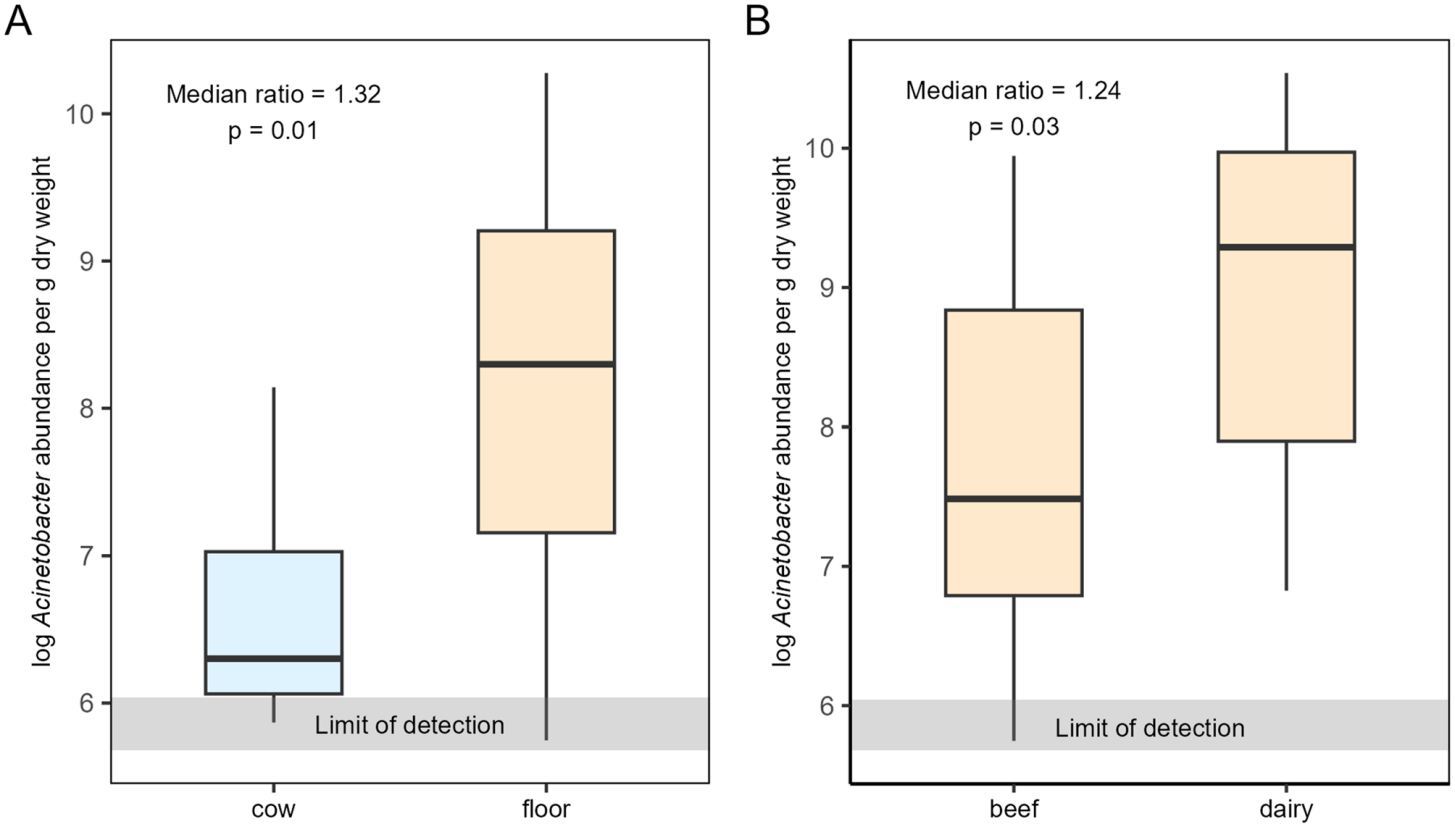
*Acinetobacter* abundance in cattle feces. Distribution of *Acinetobacter* abundance in cattle feces as determined by quantitative PCR. (**A**) Comparison of *Acinetobacter* abundance between ‘floor’ fecal samples and the corresponding per-farm mean values from ‘cow’ samples (Wilcoxon signed-rank test). (**B**) Comparison of *Acinetobacter* abundance between beef and dairy farms based on ‘floor’ fecal samples (Wilcoxon rank-sum test). Boxes represent the interquartile range (Q1– Q3), with median drawn as a horizontal line. Whiskers indicate the smallest and largest values within 1.5 times the interquartile range. The gray shading denotes the method limit of detection, adjusted for sample dry-matter content

Based on qPCR analysis, *Acinetobacter* spp. in ‘floor’ samples accounted for an average of ≈1% of the total bacteria (average absolute abundance 4.33 × 10^9^ 16S rRNA gene copies per g dry weight), whereas nearly 50% of the ‘cow’ samples fell below the detection limit of ≈10^6^ 16S rRNA gene copies per g dry weight. Reflecting this, average *Acinetobacter* abundance in ‘floor’ samples was at least an order of magnitude higher than the average per-farm value from ‘cow’ samples (*p* = 0.01) (Fig. 4A). Due to the high proportion of ‘cow’ samples below the limit of detection, subsequent analyses focused solely on the ‘floor’ samples. Here, *Acinetobacter* abundance in feces from dairy farms was at least 10 times greater than that in beef farms (*p* = 0.03; Fig. 4B), while the differences among the different stabling types remained insignificant. Among the factors tested for correlation with *Acinetobacter* abundance in ‘floor’ samples, on-farm antibiotic use showed no correlation, whereas total SCFA showed a positive correlation (Rho = 0.51, *p* = 0.03) (Fig. S16A) and the C/N ratio showed a negative correlation (Rho = −0.53, *p* = 0.03) (Fig. S16B).

### *rpoB* metabarcoding confirms the high diversity of *Acinetobacter* species and indicates the main environmental drivers of species occurrence

Metabarcoding of the taxonomic marker gene *rpoB* was used to study *Acinetobacter* taxonomic diversity and species composition. Due to the low abundance of acinetobacters in most samples, metabarcoding was performed on enrichment cultures, and presence–absence data were used to minimize potential bias arising from differential strain growth during enrichment. In total, 12,959 *rpoB* clusters were identified, with an average observed richness of 412 clusters per sample. Given that the 98% sequence identity threshold used for *rpoB* sequence clustering corresponds to subspecies-level differentiation for most *Acinetobacter* species [81], these results indicate a remarkably high *Acinetobacter* strain-level diversity in cattle feces. No significant differences in observed richness were detected across sample types, antibiotic usage, production systems, or stabling conditions.

Applying a 97% identity threshold between representative *rpoB* cluster sequences and their closest reference *rpoB* sequences (Additional file 1), we classified 6,637 *rpoB* clusters into 55 *Acinetobacter* taxa, comprising 33 validly named species and 22 putative novel taxa or unclassified singletons. Almost the same number of clusters (6,322) remained unclassified at the species level. Clusters assigned to *A. pseudolwoffii*, *A. pecorum*, *A. lwoffii* (all members of the *A. lwoffii* phylogroup), and *A. indicus* were detected in more than 80% of ‘cow’ samples, indicating that these species represent the core *Acinetobacter* species in the cattle intestine. In contrast, the next most prevalent species, *A. variabilis*, was found in only 52% of ‘cow’ samples. Clusters assigned to the four core species also occurred in more than 90% of ‘floor’ samples.

NMDS ordination was used to assess differences in *Acinetobacter* community composition (based on all *rpoB* clusters) between ‘cow’ and ‘floor’ samples from the same farm (14 farms included, Fig. S17). *Acinetobacter* communities were not clearly clustered according to sample type or farm, showing considerable between-cow variability within certain farms. However, in all but three farms, the ‘floor’ samples fell within two standard deviations of the individual ‘cow’ samples’ distance to the farm-specific centroid. This suggests that, in most cases, the ‘floor’ samples provided a representative approximation of the average *Acinetobacter* community composition among the cows on the same farm.

**Fig. 5:**
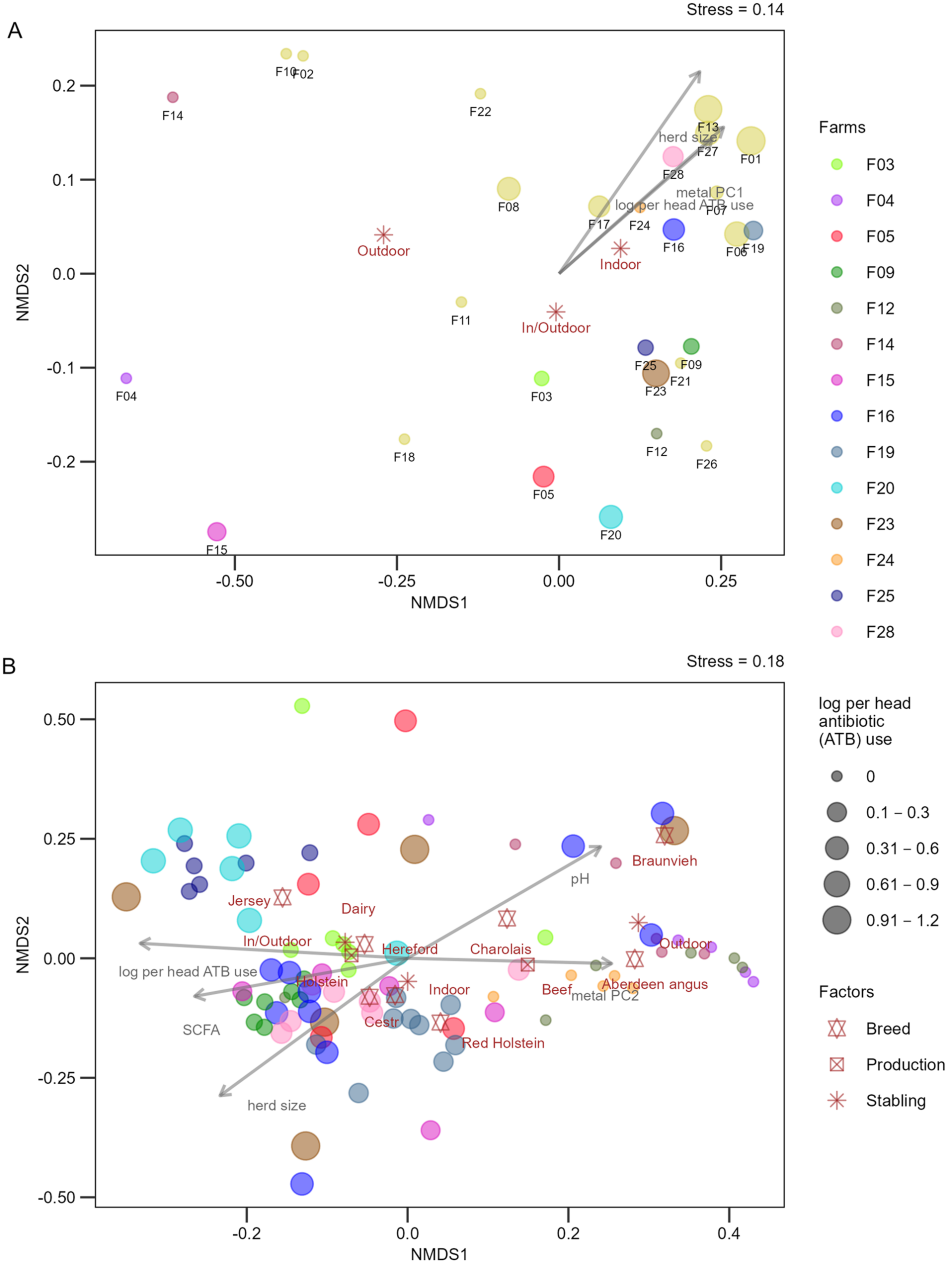
Non-metric multidimensional scaling (NMDS) analysis of *Acinetobacter* communities. NMDS plots based on the presence or absence of *Acinetobacter rpoB* clusters from ‘floor’ (**A**) and ‘cow’ (**B**) samples. The ‘floor’ samples originated from all 28 farms and are labeled with farm numbers. The ‘cow’ samples originated from a subset of 14 farms and the farm of origin is represented by color. Data point size represents log-transformed per-head antibiotic use (originally measured in g per 6 months). Farm- and cow-related explanatory variables significantly correlated with the ordinations (envfit, *p* < 0.05) are shown as vectors (for numerical variables) or centroids (for categorical variables). Abbreviations: SCFA, short-chain fatty acids; log per head ATB use, log-transformed per-head antibiotic use; metal PC1 and PC2, the first two principal components summarizing heavy metal variation (see fig. S1)

Separate NMDS ordinations were subsequently performed for ‘floor’ and ‘cow’ samples to explore *Acinetobacter* community composition in relation to selected farm and cow-specific factors (Fig. 5). The ‘floor’ samples tended to separate along NMDS1 based on stabling type (Fig. S18), with indoor floor samples being exclusively in the right part of the plot. Farms with high per-head antibiotic use (e.g. F01, F13, F27, F28) also tended to cluster together based on their ‘floor’ sample profiles, but certain farms with low or no antibiotic use (but with indoor stabling, e.g. F07 and F24) clustered with them. This indicates that *Acinetobacter* community composition in ‘floor’ samples is likely shaped by a combination of multiple factors, whose individual effects are difficult to disentangle by NMDS. Similarly, NMDS analysis based on ‘cow’ samples showed trends for clustering according to farm (e.g. F03, F09 and F25), production, stabling, and breed (e.g. Hereford and Holstein), with none of these factors showing a clear independent effect on *Acinetobacter* community composition (Fig. 5 and Fig. S18–S20). The ‘cow’ NMDS plots were further correlated with herd size, per-head antibiotic use, pH, SCFA and heavy metal content, indicating possible effects of these factors on *Acinetobacter* community composition. The high between-cow variability could not be clearly explained by recent (within six months prior to sampling) antibiotic administration. Though some of the treated cows were distant from their untreated counterparts on the NMDS plots, untreated cows from certain farms displayed high dispersion as well (Fig. S21).

**Fig. 6:**
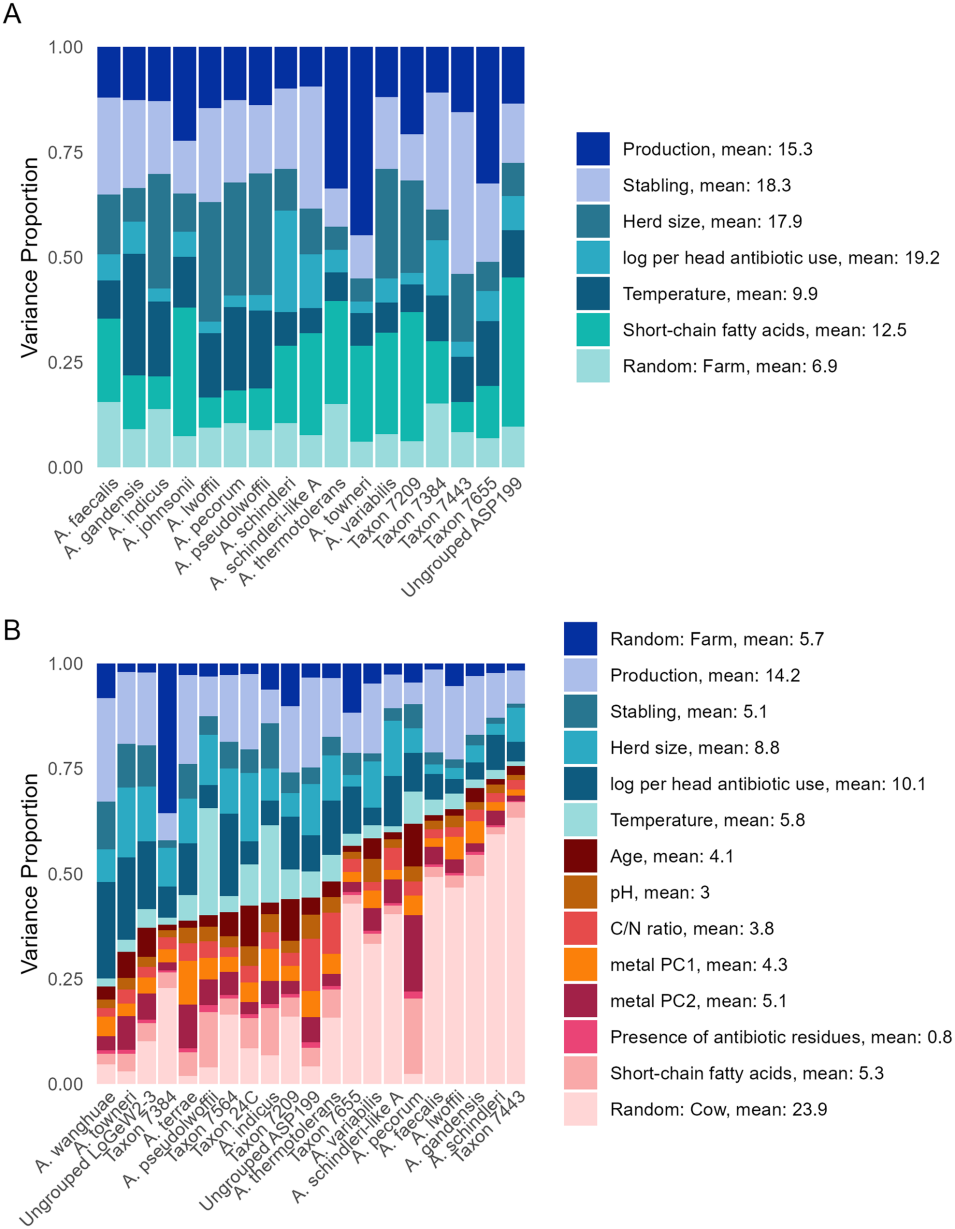
Variance partitioning of *Acinetobacter* species composition. Variance partitioning of *Acinetobacter* species composition (species occurrence based on *rpoB* metabarcoding) in ‘floor’ (**A**) and ‘cow’ (**B**) samples as determined by hierarchical modeling of species communities (HMSC). The explanatory variables in (**A**) included farm-level factors (production type, stabling, herd size, log-transformed per-head antibiotic use (= log per head antibiotic use), air temperature, and total short-chain fatty acids), and random farm effects. The explanatory variables in (**B**) included farm-level factors (as above, except total short-chain fatty acids), cow-level factors (age, pH, C/N ratio, heavy metal principal components 1 and 2 (= metal PC1 and PC2), presence of antibiotic residues, and total short-chain fatty acids), and random farm- and cow-effects. Log-transformed sequencing depth was included in both HMSC models but is not displayed. The values next to the legend represent the average variance (%) explained by individual variables across all analyzed species

To disentangle the effects of various farm- and cow-specific factors on *Acinetobacter* community composition, HMSC analysis was conducted separately on ‘floor’ and ‘cow’ samples. This analysis was done at the *Acinetobacter* species level (i.e., *rpoB* clusters classified to the same species were grouped) to facilitate interpretation. Variance partitioning (Fig. 6A) revealed that *Acinetobacter* species composition in the ‘floor’ samples was mainly determined by per-head antibiotic use (explaining on average 19.2% variance), followed by stabling (18.3%), herd size (17.9%) and production type (15.3%). These relatively similar contributions are in line with NMDS results, showing no overriding effect of any single factor. Similarly, production type, per-head antibiotic use, and herd size were the primary factors shaping *Acinetobacter* species composition in ‘cow’ samples, explaining 14.2%, 10.1%, and 8.8% of variance, respectively (Fig. 6B). In contrast, cow-specific factors such as cow age, SCFA or C/N content contributed relatively little to the explained variance. Notably, the random cow effect accounted for 23.9% variance, indicating that *Acinetobacter* community composition might be highly structured at the individual-cow level, potentially due to unmeasured host-specific factors or microenvironmental variation.

Heatmaps of species niches based on HMSC β-parameters revealed both common and contrasting responses of the individual *Acinetobacter* species to various farm- and cow-level factors (Fig. S22). Most of the *Acinetobacter* spp. (as identified with *rpoB* metabarcoding) exhibited a higher likelihood of occurrence in dairy farms as compared to beef farms, both in ‘floor’ and individual ‘cow’ fecal samples. Most species were also positively associated with high levels of SCFA in feces; notably *A. gandensis*, *A. indicus*, *A. pecorum*, and *A. pseudolwoffii* showed a significant positive relationship (with >90% posterior support) in both ‘floor’ and ‘cow’ samples. In addition, the majority of species were positively associated with higher sampling temperatures (related to the summer sampling season).

In contrast, antibiotic use at the farm level was negatively associated with the occurrence of most *Acinetobacter* species in ‘cow’ and ‘floor’ fecal samples, with the only exception being Taxon 7384 in ‘floor’ samples, which showed a significant positive association. Herd size had an overall negative effect on the occurrence of *Acinetobacter* spp. in ‘cow’ samples, whereas in ‘floor’ samples, this effect was species specific, with *A. thermotolerans* and *A. towneri* showing significant preference for larger herds. Considering stabling, contrasting results were obtained for ‘floor’ and ‘cow’ samples. All species detected in the ‘floor’ samples showed a positive trend towards indoor conditions, while it was generally the opposite for ‘cow’ samples, with the main exception being *A. indicus*. Interestingly, *A. pecorum*, *A. terrae*, and *A. variabilis* present in ‘cow’ fecal samples were significantly positively associated with metal PC2, representing higher Pb and Cd content.

### Shotgun sequencing of enrichment cultures provides *Acinetobacter* MAG and plasmid sequences

Shotgun sequencing of *Acinetobacter* enrichment cultures from 28 ‘floor’ samples yielded 11,250 contigs (287 Mb in total) affiliated with the genus *Acinetobacter* (Table S10). Contigs longer than 250,000 bp, representing *Acinetobacter* chromosomes or large chromosome fragments, were further classified at the species level using the ANIb approach (Table S12). Of the 207 contigs examined, 116 were successfully classified to known species based on the 96% ANIb threshold [82]. They represented *A. faecalis*, *A. gandensis*, *A. indicus*, *A. pecorum*, *A. pseudolwoffii*, and *A. schindleri*. Six contigs exhibited > 99% completeness and <5% contamination and represented single-contig, circular, high-quality metagenome-assembled genomes (MAGs) [83]. Two of these MAGs were classified as *A. indicus* and *A. pecorum* based on the 96% ANIb threshold, while one showed an ANIb value of 95.7% to *A. amyesii*. The three remaining MAGs (F01_chromosome_61, F18_chromosome_3, and F22_chromosome_7) likely represent novel *Acinetobacter* taxa, sharing 99.4–100% identity across a 355-bp fragment of the *rpoB* with reference strains of Taxon 7683, Taxon 7579, and Taxon 7655, respectively.

In total, 599 putative plasmid contigs were identified across the *Acinetobacter* assemblies, of which 514 showed significant sequence similarity to PLSDB entries (Table S12) and 213 contained homologues of plasmid replication initiation (*rep*) genes listed in the Acinetobacter Plasmid Typing database (128 of these contigs met both criteria; Table S13). Among the 213 *rep* homologues, 120 could be confidently assigned to known Rep types (Table S13), belonging to the three major families, i.e. R1, R3, and RP, with R3 predominating. The remaining 93 *rep* genes shared < 95% nucleotide sequence identity with described Rep types and likely represent novel variants. The *rep* genes corresponding to LowGC-type plasmids (i.e., R3–T20 type) were not detected in the *Acinetobacter* assemblies.

### Shotgun sequencing of enrichment cultures revealed a rich *Acinetobacter* resistome with potential to spread clinically important antibiotic resistance genes

**Fig. 7:**
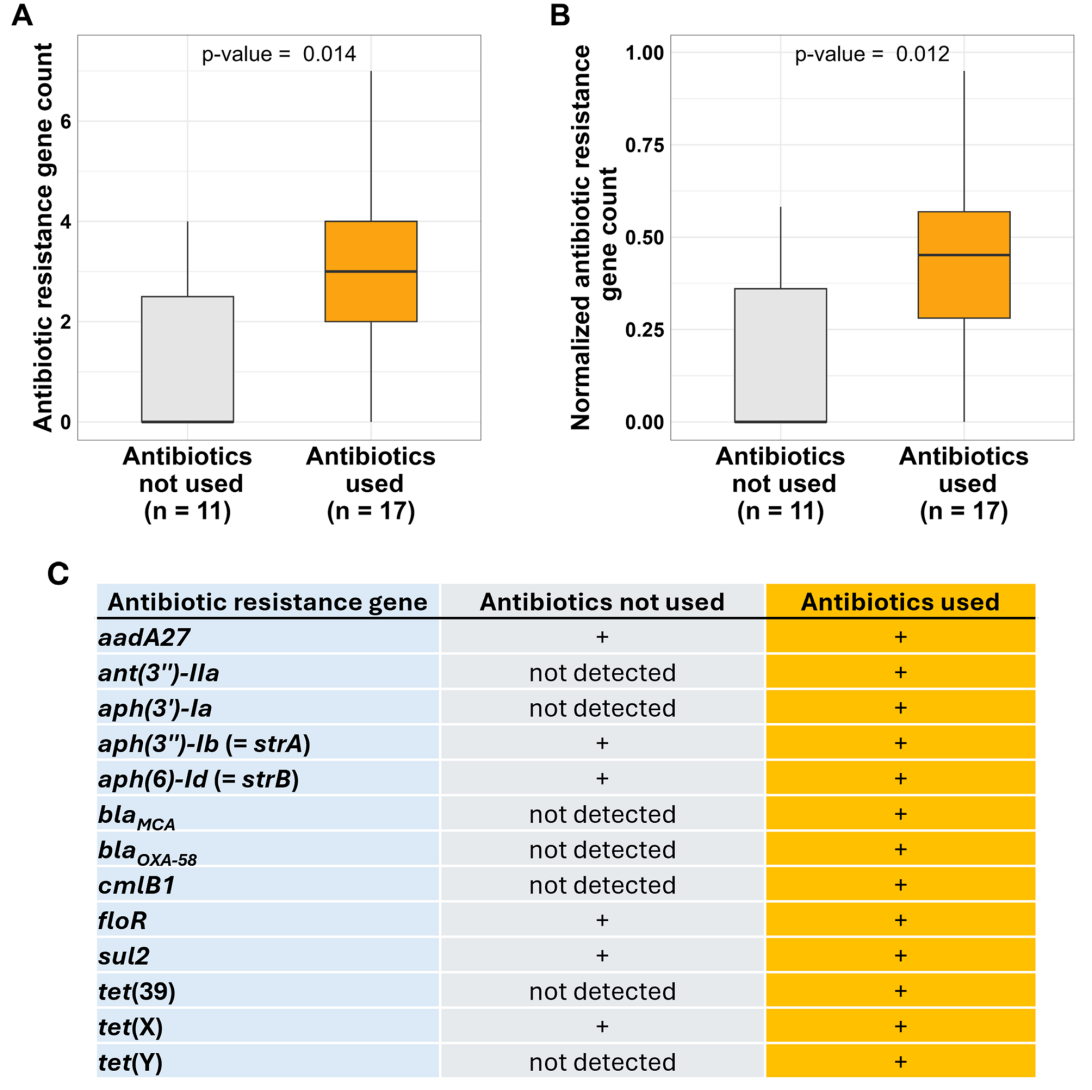
Acquired antibiotic resistance genes in *Acinetobacter* shotgun metagenome. (**A**) Number of unique antibiotic resistance genes (richness) detected per farm, compared between antibiotic-using (“antibiotics used”) and antibiotic-free (“antibiotics not used”) farms using Wilcoxon-rank sum test. (**B**) Same data as in plot A, but normalized by log(assembly length). Boxes represent the interquartile range (Q1–Q3), with the median drawn as a horizontal line. Whiskers represent the smallest and largest values within 1.5 times the interquartile range from the quartiles. (**C**) Overview of acquired antibiotic resistance genes detected in antibiotic-free and antibiotic-using farms

Abricate analysis revealed the presence of 19 distinct ARGs across the *Acinetobacter* assemblies, with a total of 116 Abricate hits (Fig. 7, Table S14). These ARGs were predicted to confer resistance to aminoglycosides, amphenicols, β-lactams (including carbapenems), tetracyclines, and sulfonamides. Of these, 78 ARGs were located on plasmid-derived contigs.

Our further analyses focused on horizontally acquired ARGs, excluding chromosomally encoded intrinsic carbapenemase genes, which do not confer significant carbapenem resistance unless placed under a strong promoter provided by an upstream insertion sequence [84]. Therefore, we examined the genetic context of all identified carbapenemase genes and excluded those lacking adjacent insertion sequences. These carbapenemase genes (identified by Abricate as *bla*_OXA-235_, *bla*_OXA-282_, *bla*_OXA-258_, *bla*_OXA-537_, *bla*_OXA-646_, and *bla*_OXA-648_) were all located between the metalloprotease and molecular chaperone DnaK genes, suggesting a chromosomal origin. Thus, the only carbapenemase genes retained for further analyses were the ones bracketed by IS*Aba3* and present on contigs classified as *Acinetobacter* plasmids; their sequences were 100% identical to the *bla*_OXA-58_ sequence of *A. baumannii* MAD (GenBank accession number AY665723). All other identified ARGs are either known to be horizontally transferable or were associated with mobile genetic elements. The complete set of horizontally transferable ARGs was then compared between antibiotic-using and antibiotic-free farms (Fig. 7, Fig. S23). We detected significantly more horizontally transferable ARG types in antibiotic-using farms, as compared to antibiotic-free farms (*p* = 0.014), though the effect of antibiotic usage was small (median = 3 and 0 ARGs, respectively).

**Fig. 8:**
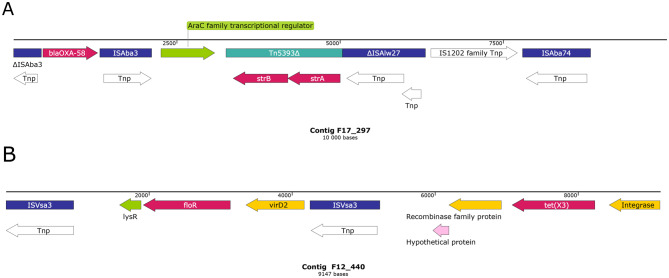
Metagenomic contigs carrying *bla*_OXA-58_ and *tet*(X3). (**A**) Contig F17_297 carrying the carbapenemase resistance gene *bla*_OXA-58_, flanked by insertion sequence IS*Aba3*, as well as the streptomycin resistance genes *strA* and *strB.* The total length of the contig is 35,741 bp, with only the first 10,000 bp shown. (**B**) Contig F12_440 carrying the tigecycline resistance gene *tet*(X3), flanked by recombinase and integrase genes, as well as the florfenicol resistance gene *floR.* Arrows labeled ‘tnp’ indicate transposase-coding regions

Notably, the *bla*_OXA-58_ gene was found only in farms where antibiotics were used. Carbapenems are not administered to cattle, suggesting that these genes may be co-selected with other antibiotic or heavy metal resistance genes in the farms. Supporting this, genetic analyses showed co-localization of the *bla*_OXA-58_ gene with the *strA-strB* genes on two contigs (Fig. 8A), indicating that carbapenem resistance may be co-selected with aminoglycosides, which are frequently applied in cattle.

Another important case of co-localization is the presence of *tet*(X3) and *floR* on a single contig (Fig. 8B). While *tet*(X3) confers resistance to the last-resort antibiotic tigecycline, *floR* confers resistance to florfenicol, which is occasionally used on cattle farms. The co-localization of *bla*_OXA-58_ and *tet*(X3) with mobile genetic elements (Fig. 8AB) on contigs sharing highly similar regions with *Acinetobacter* plasmids suggests their potential for dissemination through horizontal gene transfer. Of note, another contig carrying *tet*(X3) (F20_1806) showed 100% identity to the LowGC-type plasmid pHH1107 [18] over 5,117 bp, but it lacked the *rep* gene required for reliable classification.

A total of 466 HMRG hits were obtained across the *Acinetobacter* assemblies, representing 19 distinct HMRG types (Table S15). The most frequently (>10 occurrences) detected HMRGs were *golT* (gold/copper resistance), *arsB, arsC*, and *arsH* (arsenic resistance), *dpsA* (iron resistance), *czcA* and *czcD* (cadmium/zinc/cobalt resistance), and *nreB* (cobalt/nickel resistance). Only 37 HMRGs were localized on plasmid contigs. Co-localization of HMRGs with ARGs was rare, observed on only eight contigs, and primarily involved intrinsic carbapenemase genes on contigs likely representing *Acinetobacter* chromosomes. In a single instance, the *dpsA* gene co-occurred with *strA* and *strB*, although not in close proximity. Overall, these findings indicate that co-selection of heavy metal and antibiotic resistance genes is uncommon in the studied farms. This is further supported by the finding that HMRG counts did not differ significantly between antibiotic-using and antibiotic-free farms (Fig. S24).

### Genome sequencing confirms the presence of *bla*_OXA-58_ on *A. pseudolwoffii* ANC 7493 plasmid

**Fig. 9:**
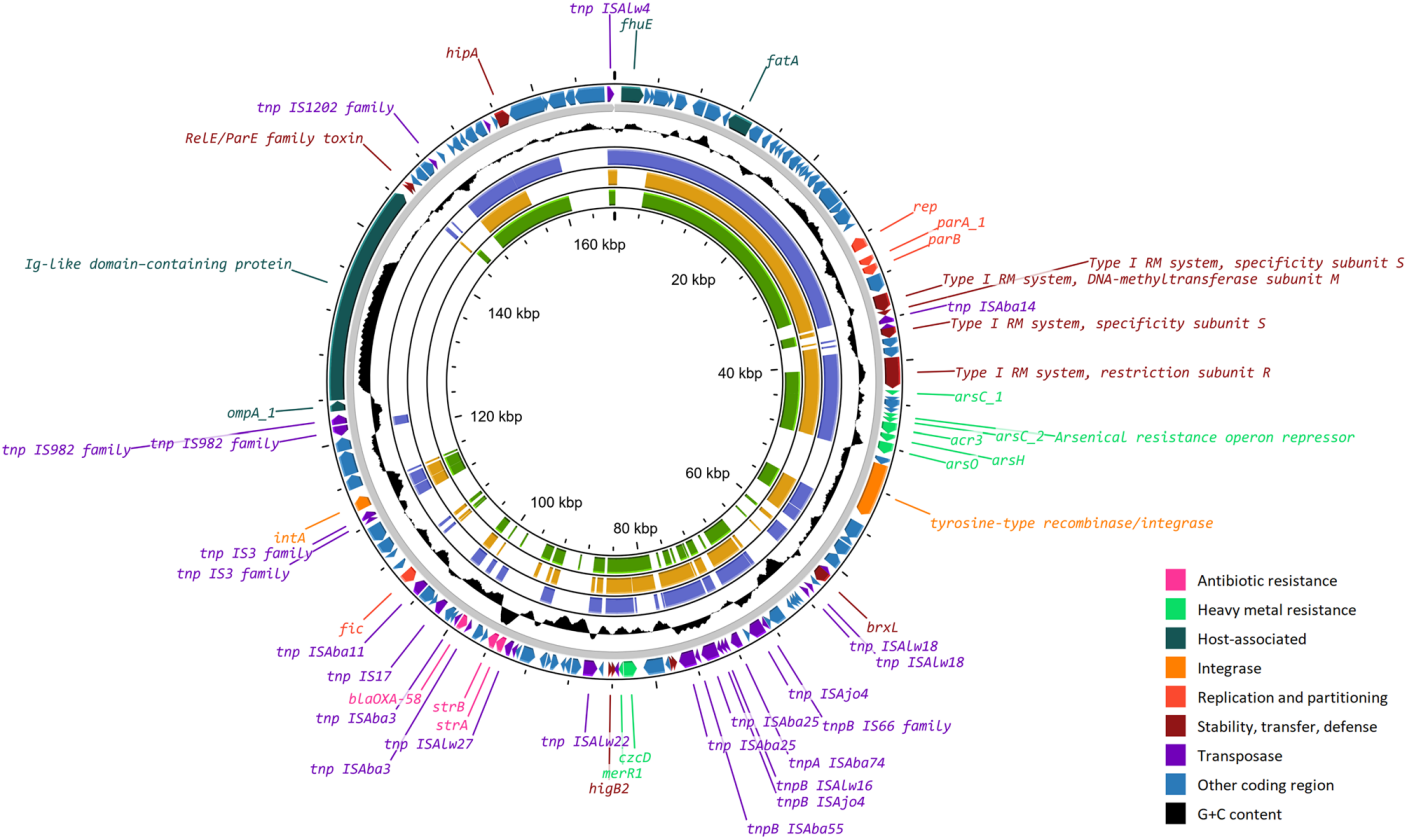
Plasmid map of pANC7493.1 and its comparison to similar plasmids. Genetic content of the plasmid pANC7493.1 obtained from the whole-genome sequencing of *A. pseudolwoffii* ANC 7493. The total length of the plasmid is 167,549 bp. The outermost ring depicts the predicted coding regions, color-coded according to their putative functions (see color legend). The second ring represents the plasmid G+C content, while the third, fourth, and fifth rings (from outside) show BLASTn alignments (Prokka; E-value < 1e-10) with the most similar plasmids from the NCBI nr/nt database (accessed 2025-Oct-22): CP084301.1 (blue), CP183904.1 (gold), and CP183900.1 (green)

The genome assembly of *A. pseudolwoffii* ANC 7493 consisted of a single circular chromosome (2,764,499 bp) and one circular plasmid (167,549 bp; designated pANC7493.1). Abricate analysis confirmed the presence of the *bla*_OXA-58_, *strA*, and *strB* genes in the genome, all located in close proximity on the plasmid pANC7493.1 (Fig. 9). These genes were part of a region spanning positions 93,403–100,027 bp, which shared > 99.9% sequence identity with the metagenomic contig F17_297 (positions 1–6,322), differing only by the absence of a LysE family translocator gene between *strB* and an AraC family transcriptional regulator gene from the contig (Fig. S25). The *bla*_OXA-58_ was flanked by IS*Aba3* insertion sequences, with the upstream copy being truncated (i.e., IS*Aba3-*like element).

Plasmid pANC7493.1 contained a single *rep* gene, enabling its classification as the R3–T103 type according to the Acinetobacter Plasmid Typing scheme [59]. No relaxase or mating-pair formation genes were detected, suggesting that the plasmid is neither mobilizable nor self-transmissible. In addition, the plasmid encoded several stability and maintenance systems, including restriction–modification and toxin–antitoxin modules, as well as metabolic and putative host-adaptive genes. These included the outer membrane protein A gene *ompA*, a known *A. baumannii* virulence factor [85], a large gene encoding Ig-like domain-containing protein typical of biofilm-associated proteins [86], and two siderophore receptor genes, *fhuE* and *fatA*. The plasmid also carried several HMRGs, including an arsenic resistance operon, the cadmium/cobalt/zinc resistance gene *czcD*, and a mercuric resistance operon regulator gene *merR1*.

According to NCBI BLASTn results, the highest sequence coverage between pANC7493.1 and entries in the NCBI nucleotide collection was 52%, indicating that the plasmid represents a novel genetic element. The best BLASTn matches were three *A. pseudolwoffii* plasmids: CP084301.1 (52% coverage, 98.35% identity) and CP183900.1 and CP183904.1 (both 46% coverage, 98.79% identity), originating from chicken and bovine samples in China. Mapping of these plasmid sequences onto pANC7493.1 (Fig. 9) revealed that while all four plasmids shared HMRG regions, the ARG region was unique to pANC7493.1.

## Discussion

The cattle fecal microbiome is important for both animal and human health, particularly regarding zoonotic transmission and the agricultural application of cattle manure. Multiple studies have characterized its composition and consistently reported a predominance of members of the bacterial phyla *Bacteroidetes* and *Firmicutes*, whereas *Proteobacteria* generally account for less than 5% of the total bacterial community [87, 88]. While most studies on *Proteobacteria* have focused on *Escherichia coli* [89, 90], *Acinetobacter* species remain underexplored despite their importance in pathogenesis and dissemination of ARGs [13, 15, 16]. To address the low abundance of *Acinetobacter* in cattle feces, we combined culturing strains with metabarcoding and metagenomic analysis of enrichment cultures. This approach uncovered a rich diversity of *Acinetobacter* species, including putative novel species, and provided insights into their response to antibiotic selection pressure.

Both pure strain isolation and *rpoB* metabarcoding revealed a high diversity of *Acinetobacter* spp. in cattle feces. Of the 284 strains recovered, 63% were assigned to 16 validly named species, whereas the remaining 37% comprised either putative novel species awaiting formal description or unclassified singletons (Table 1). Strains obtained in this study have already contributed to the delineation of *A. amyesii* [91] and *A. thermotolerans* [32] as well as to the emended description of *A. faecalis* [80]. Thus, the share of strains belonging to described species prior to the start of this project was only ≈48%, highlighting that cattle represent a reservoir of largely unexploited *Acinetobacter* diversity. It should be noted that some of the novel *Acinetobacter* taxa, including *A. thermotolerans*, were recovered at the cultivation temperature of 44 °C. As growth at elevated temperatures is considered a prerequisite for mammalian pathogenicity (this temperature was used to improve *A. baumannii* recovery), these taxa warrant further investigation regarding their potential virulence.

The *rpoB* sequences obtained from metabarcoding of enrichment cultures were classified into 33 validly named species and 22 putative novel species or taxonomically unique singletons, comprising the aforementioned taxa and singletons included in our custom *rpoB* database. The higher species count relative to isolate-based data was anticipated, as strain isolation requires an additional culture step on agar plates, where certain taxa may fail to form colonies on ACE agar, thus limiting the number of isolates that can be examined. In addition, this analysis was conducted across ‘cow’ and ‘floor’ samples, whereas only ‘floor’ samples were used for strain isolation because of the low throughput of the isolate-based approach. Nonetheless, the taxonomic assignments based on *rpoB* metabarcoding need to be interpreted with caution, as they were not complemented by additional methods such as MALDI-TOF MS profiling or phenotypic characterization, which could be applied only to the strains. Although the number of species detected with either method may appear high given that only 87 *Acinetobacter* species are currently validly described (https://szu.gov.cz/wp-content/anemec/Classification.pdf), previous studies have indicated that a substantial portion of the genus’s phylogenetic diversity remains undescribed [17, 92]. The diversity revealed in this study may thus represent only a small fraction of the genus’s taxonomic breadth, highlighting the need for continued exploration of *Acinetobacter* in animal-associated environments.

The core *Acinetobacter* species detected in most cattle fecal samples based on *rpoB* metabarcoding were *A. indicus* and members of the *A. lwoffii* phylogroup (*A. lwoffii*, *A. pecorum*, and *A. pseudolwoffii*). Consistently, *A. pseudolwoffii* and *A. indicus* were also represented by the largest number of isolated strains, whereas strains belonging to other members of the *A. lwoffii* phylogroup were recovered at lower frequencies (Table 1). The occurrence of *A. indicus*, *A. lwoffii*, and *A. pseudolwoffii* in cattle manure has been documented previously [13, 24, 93]. In contrast, *A. pecorum* was only recently described based on isolates from sheep and chickens [94]. Our study thus demonstrates the presence of *A. pecorum* in cattle feces and provides the first complete MAG of this species from cattle. Compared with the core taxa, *A. baumannii* was detected less frequently, occurring in fewer than 5% of ‘cow’ and ‘floor’ samples by metabarcoding of enrichment cultures and in 21% of ‘floor’ samples by strain isolation (using two different growth temperatures). These findings are consistent with previous studies, which suggested that *A. baumannii* likely originates from environmental sources rather than being a primary colonizer of cattle [7, 10].

This raises the question of whether the *Acinetobacter* species and taxa detected in this study are capable of stable colonization of the cattle intestine, or whether they represent transient passengers of the intestinal tract. *Acinetobacter* species are strict aerobes [17] and thus appear unlikely to thrive under the largely anaerobic conditions of the cattle gut. However, *A. baumannii* has been shown to proliferate in the colonic crypts of mice [95], suggesting the existence of intestinal micro-niches with oxygen levels sufficient to support *Acinetobacter* growth. Based on our data, we propose that the *Acinetobacter* communities observed in cattle feces consist of both stable colonizers—represented in particular by the core species identified in this study—and transient species acquired from environmental sources, such as *A. baumannii* and other less frequently detected taxa. This interpretation is further supported by our metabarcoding analysis of species composition in cattle feces, which revealed that farm- and cow-associated variables explained only a limited portion of the variation, while a substantial proportion (on average 24%) remained attributable to a random cow effect (Fig. 6). When examined by species, the random cow effect was low for *A. indicus*, *A. pecorum*, and *A. pseudolwoffii* (≈2–7%), supporting their role as stable colonizers, whereas it was substantially higher for *A. lwoffii* (47%).

At the genus level, *Acinetobacter* abundance was substantially lower in ‘cow’ samples (feces collected per rectum or immediately upon defecation) than in ‘floor’ samples (feces deposited on the farm floor). This observation further supports the notion that growth of *Acinetobacter* in the cattle intestine is limited, whereas these bacteria can rapidly proliferate once exposed to external conditions. The increased abundance in deposited feces may result from rapid growth under oxygen-rich conditions, supported by the availability of short-chain fatty acids in cattle feces as a suitable carbon source, and by the competitive advantage over major intestinal taxa that favor anaerobic conditions (e.g., *Bacteroidetes* and *Firmicutes* [87]). Additional contributions could arise from colonization by airborne *Acinetobacter* – an aspect that merits further investigation. Observed abundances in deposited feces (typically 10^7^−10^9^ 16S rRNA gene copies per gram of dry weight, Fig. 4) were comparable to values reported in manure inputs for biogas plants in Germany [24], where 10^6^−10^8^copies per gram of fresh material were detected, equivalent to roughly tenfold higher levels on a dry weight basis. Notably, *Acinetobacter* abundance was higher in dairy than beef cattle samples (Fig. 4), with most species showing a general preference for dairy farms (Fig. S22). Previous studies have documented differences in the intestinal microbiome between beef and dairy cattle [87] and variation in *A. baumannii* isolation rates [7]. This result is difficult to explain based on the current data, but it may be related to particular cow breeds, types of nutrition or higher levels of human contact in dairy farms.

Analysis of the antibiotic susceptibility phenotypes of strains supported the hypothesis that ongoing antibiotic use in Czech cattle farming provides selective pressure for resistance acquisition. Based on data from two core species, *A. pseudolwoffii* and *A. indicus*, strains from antibiotic-using farms displayed lower streptomycin susceptibility than those from antibiotic-free farms (Fig. 3). Moreover, three MDR strains identified in this study originated exclusively from antibiotic-using farms. These strains belonged to the recently described species *A. faecalis* [80], *A. thermotolerans* [32], and a novel taxon (Taxon 7209), underscoring the potential role of newly recognized taxa in the dissemination of antimicrobial resistance. Nevertheless, reduced antibiotic susceptibility in these strains was limited to antibiotics not classified as critically important for human medicine (i.e., tetracycline, streptomycin, sulfamethoxazole, and trimethoprim). This contrasts with findings from China, where MDR *Acinetobacter* isolates displayed resistance to clinically critical agents such as carbapenems and tigecycline [13, 15]. Consistent with this, all *A. baumannii* isolates recovered in this study were susceptible to all clinically relevant antibiotics.

Even though strain-level analyses suggested that the overall health risk associated with Czech cattle farms is low, resistome profiling by shotgun metagenomic sequencing of enrichment cultures revealed the presence of clinically relevant ARGs, which were further corroborated by strain-level data. Notably, the carbapenemase gene *bla*_OXA-58_ was detected on several sequence contigs from antibiotic-using farms and was confirmed in three strains of *A. pseudolwoffii*. All three strains were susceptible to carbapenems, but the inconsistency between genotype and phenotype is not uncommon [96], likely owing to insufficient gene expression. The co-localization of *bla*_OXA-58_ with *strA-strB* (either on the same contig in metagenomic data or within the same strains) suggests that carbapenem resistance may be co-selected by aminoglycoside use in farm settings. In addition, metagenomic data indicated a possible co-selection of the tigecycline resistance gene *tet*(X) with florfenicol. Both gene clusters are associated with transposable elements and plasmids, indicating their potential for high mobility within and between bacterial hosts (Fig. 8). Together, these findings highlight the capacity of cattle-associated *Acinetobacter* spp. to serve as reservoirs of clinically relevant resistance determinants that could, under suitable conditions, be mobilized into pathogenic bacteria.

The co-localization of *bla*_OXA-58_ with *strA* and *strB* on a plasmid was confirmed through whole-genome sequencing of one of the *bla*_OXA-58_-positive strains, *A. pseudolwoffii* ANC 7493 (Fig. 9 and Fig. S25). Since this strain was susceptible to carbapenems, we assume that the IS*Aba3*-like element located upstream of *bla*_OXA-58_ does not provide a strong promoter sufficient for *bla*_OXA-58_ expression within this host. The expression of *bla*_OXA-58_ in *A. baumannii* and other *Acinetobacter* spp. may be enhanced by the insertion of IS*Aba2,* IS*18*, and other insertion sequence types within the IS*Aba3*-like element through the provision of hybrid promoter sequences [97, 98], but these structures were not observed here. The plasmid pANC7493.1 appears to be non-mobilizable and non–self-transmissible, but since natural competence is widespread among *Acinetobacter* spp. [92], horizontal gene transfer via transformation cannot be ruled out. Although the plasmid appears to be novel, similar scaffolds have been recovered from *A. pseudolwoffii* isolates obtained from chicken and bovine samples in China (Fig. 9), suggesting that related plasmids may circulate globally.

PCR screening of *Acinetobacter* strains further identified the hosts of the *sul2*, *strA-strB*, and *tet*(Y) genes, which have been reported from farm environments in Europe and elsewhere [2, 18, 99]. The *strA-strB* genes were particularly widespread, occurring in 10 species or novel taxa, likely reflecting the frequent administration of aminoglycosides (e.g., dihydrostreptomycin) in cattle. Regarding aminoglycoside resistance, we also demonstrated that *aadA27*, encoding the ANT(3’’)-II aminoglycoside nucleotidyltransferase and originally described in *A. lwoffii* from permafrost [100], is present in at least four species or novel taxa (including *A. faecalis*, in which we recently reported the gene [80]). These findings highlight the widespread dissemination of aminoglycoside resistance determinants across diverse *Acinetobacter* lineages, suggesting that they may constitute a long-standing component of the environmental resistome.

LowGC-type plasmids, previously thought to mediate the transfer of ARGs from livestock manure to soil and to contribute to the environmental spread of antibiotic resistance [2, 18, 20], were scarce in our dataset. They were detected only in three *Acinetobacter* strains (i.e., 1%), which were mostly susceptible to antibiotics. Within the scope of this study, therefore, LowGC-type plasmids do not appear to represent a dominant vehicle for ARG dissemination. Nonetheless, our results newly identify their hosts as *A. variabilis* and members of the *A. lwoffii* phylogroup, including *A. pecorum*—information that was overlooked in earlier studies, which had recovered such plasmids mainly from manured soil via exogenous plasmid isolation [2, 18]. By contrast, we detected several other Rep-type plasmids (mostly of the Rep3 family) in *Acinetobacter* enrichment cultures from cattle feces, some of which carried ARGs such as *strA-strB.* The above-mentioned plasmid pANC7493.1 carrying *strA-strB* and *bla*_OXA-58_ also belonged to the Rep3 family. Reference plasmids of these Rep-types have been reported from diverse geographical regions and sources, including animal feces, clinical isolates, and hospital sewage (Table S12; [59]), indicating their broad host range and global circulation. Together, these findings suggest that while LowGC-type plasmids may not be central to ARG spread in cattle, cattle-associated acinetobacters nonetheless harbor plasmid backbones capable of facilitating the dissemination of resistance genes across environments and host species.

Livestock manure represents an environment conducive to horizontal gene transfer of ARGs due to its high bacterial density and diversity, abundant nutrients, and the presence of antibiotic residues exerting selective pressure [101]. We assume similar conditions apply to *Acinetobacter* spp. in cattle feces, given their high diversity, access to suitable carbon sources such as short-chain fatty acids, and increased abundance in feces deposited on the farm floor, which indicates active growth. Antibiotic residues were also present in both ‘cow’ and ‘floor’ samples in our study; however, some commonly used antibiotics, such as certain β-lactams, were not detected, likely due to β-lactam ring lability and, potentially, microbial degradation in cattle feces [21, 102]. In addition, heavy metals were consistently present in all fecal samples, representing another selective factor that could potentially drive co-selection of antibiotic resistance [21]. The concentrations of Cu, Zn, Cd, Pb, Cr, and As were within the ranges reported in cattle farms [23, 103], indicating that such metal contamination is likely widespread. Consistently, our shotgun metagenome analysis identified 19 distinct types of heavy metal resistance genes. However, they were infrequently localized on plasmid contigs and, in a single instance only, co-occurred on the same contig with horizontally acquired antibiotic resistance genes. Although the co-selection of heavy metal and antibiotic resistance cannot be entirely excluded – particularly given the presence of multiple heavy metal resistance genes on plasmid pANC7493.1 (Fig. 9) – the overall data indicate that such events are infrequent in the studied farm environments.

This study demonstrates the value of an integrative approach for characterizing low-abundance members of the animal microbiome. While each of the methods used carries inherent strengths and limitations, together they provided a complementary and comprehensive picture of *Acinetobacter* populations in cattle feces. The analysis of total community DNA (i.e., without culturing) enabled accurate assessment of *Acinetobacter* abundance. Although this strategy is now widely employed for diversity profiling through metabarcoding [104], it was not feasible in our study because most samples did not yield *Acinetobacter*-specific amplicons. Consequently, our diversity assessment relied on enrichment cultures. This approach is, however, inherently biased, as species or strains differ in their growth performance in the liquid ACE medium used for enrichment. To mitigate this bias, we based our analysis strictly on presence–absence data rather than relative abundances. Still, it must be acknowledged that some slow-growing strains may have remained below the detection threshold, whereas fast-growing strains were more readily detected. Such biases are not unique to our enrichment strategy but also occur in widely used PCR-based community profiling approaches employing universal bacterial primers [104].

Coupling enrichment cultures with shotgun metagenomics provided valuable insights into the *Acinetobacter* resistome without the need to sequence individual strains. Unlike *rpoB* metabarcoding, this approach did not rely on *Acinetobacter*-specific primers, making it necessary to carefully filter the data for non-*Acinetobacter* sequences. This precaution was important because non-*Acinetobacter* bacteria may also proliferate in liquid ACE medium, particularly when traces of alternative carbon sources from cattle feces are present. As a result, ARGs carried on broad-host-range plasmids or represented by contigs too short for confident taxonomic assignment may have remained undetected in this study. Nevertheless, this approach is very useful for revealing the resistome of low-abundance taxa from complex samples, as previously shown by Marano et al. [105].

The culture of pure strains offered the advantage of direct phenotypic testing, which could then be linked to genotypic traits through PCR or whole-genome sequencing. Our use of ACE medium for isolation has previously been demonstrated to be effective for recovering a broad range of *Acinetobacter* spp. from diverse environments [17]. Combined with MALDI-TOF MS (amended with a custom *Acinetobacter* database) and sequencing of a variable region of the *rpoB* gene [33], this approach allowed for reliable species identification and delineation of novel taxonomic clusters.

## Conclusions

The present study provides considerable insights into the taxonomic diversity of *Acinetobacter* and its antimicrobial resistance in cattle feces. Our findings revealed a surprisingly high diversity of *Acinetobacter* species, including several putative novel species. We identified *A. indicus*, *A. pseudolwoffii*, and other members of the *A. lwoffii* phylogroup as core *Acinetobacter* species associated with cattle. Consistent with previous reports, *A. baumannii* was rare in cattle feces, and the strains recovered here did not appear to pose an immediate health risk. Nevertheless, we detected clinically relevant resistance genes, including *bla*_OXA-58_ and *tet*(X), in cattle-associated *Acinetobacter*, despite relatively strict antibiotic use regulations on Czech farms. The association of these genes with mobile genetic elements highlights their potential for dissemination under favorable conditions and emphasizes the need for continued improvements in cattle health management to further reduce reliance on antibiotics.

## Electronic supplementary material

Below is the link to the electronic supplementary material.

Supplementary material 1

Supplementary material 2

Supplementary material 3

Supplementary material 4

Supplementary material 5

Supplementary material 6

## Data Availability

The nucleotide sequence datasets generated during the current study are available as follows. Partial *rpoB* sequences from 284 *Acinetobacter* strains are available at the NCBI GenBank repository (https://www.ncbi.nlm.nih.gov/nuccore/) under accession numbers PX405702–PX405985. Partial sequences of *aadA27*, *bla*_OXA-58_, *tet*(Y), *sul2*, and *rep* amplified from *Acinetobacter* strains are available at NCBI GenBank under accession numbers PX380145–PX380148, PX359217–PX359219, PX359220–PX359221, PX380135–PX380144, and PX396045–PX396047, respectively. Raw reads corresponding to *rpoB* metabarcoding data (Illumina MiSeq) from 118 cattle feces samples are available at NCBI Sequence Read Archive (SRA; https://www.ncbi.nlm.nih.gov/sra/), under accession numbers SRR34872364–SRR34872481. Raw Illumina NovaSeq and Oxford Nanopore reads from 28 shotgun metagenomes are available at NCBI SRA under accession numbers SRR34902429–SRR34902456 and SRR34878076–SRR34878103, respectively, and the corresponding assemblies are available at the Zenodo repository (https://zenodo.org/) under the identifier 17176853. The six high-quality *Acinetobacter* metagenome-assembled genomes are available at the NCBI GenBank repository under the accession numbers JBVOSW000000000, JBVOSX000000000, JBVOSY000000000, JBVOSZ000000000, JBVOTA000000000, and JBVOTB000000000. The complete genome of *A. pseudolwoffii* ANC 7493 is available at the NCBI GenBank repository under the accession number JBSSNL000000000. The R scripts used for HMSC analysis are available at the Zenodo repository under the identifier 17426203. Other data generated or analyzed during this study are included in this published article as supplementary tables or additional files.
