# Supplementary material for: Cattle feces are a reservoir of diverse Acinetobacter species with potential to spread antibiotic resistance genes
Source: Anim Microbiome. 2026 Apr 22;8:69. doi: 10.1186/s42523-026-00568-3 (PMC13185236; doi:10.1186/s42523-026-00568-3)
Supplement: Supplementary file 5 — Supplementary material 5 [file 42523_2026_568_MOESM5_ESM.pdf]

Supplementary figures

Cattle feces are a reservoir of diverse *Acinetobacter* species with potential to spread antibiotic resistance genes

Anitha Ravi[1,2], Violetta Shestivska[3], Priscila Thiago Dobbler[1], Hana Sechovcová[4,5], Martina Maixnerová[3], Jaroslav Semerád[6,7], Alena Nehasilová[6,7], Mariana Vadroňová[4], Iñaki Odriozola[1], Hana Šubrtová Salmonová[4], Tomáš Větrovský[1], Šárka Musilová[4], Tomáš Cajthaml[6,7], Eva Pěchoučková[4], Alexandr Nemec[3,8*], and Martina Kyselková[1*]

[1] Laboratory of Environmental Microbiology, Institute of Microbiology of the Czech Academy of Sciences, Vídeňská 1083, 142 20 Prague 4, Czech Republic

[2] Faculty of Science, Charles University in Prague, Albertov 6, 128 00, Prague 2, Czech Republic

[3] Laboratory of Bacterial Genetics, Centre for Epidemiology and Microbiology, National Institute of Public Health, Šrobárova 48, 100 00 Prague 10, Czech Republic

[4] Department of Microbiology, Nutrition and Dietetics, Faculty of Agrobiology, Food and Natural Resources, Czech University of Life Sciences, Kamýcká 129, 165 00 Prague 6, Czech Republic

[5] Laboratory of Anaerobic Microbiology, Institute of Animal Physiology and Genetics, Academy of Sciences of the Czech Republic, Vídeňská 1083, 142 20, Prague 4, Czech Republic

[6] Laboratory of Environmental Biotechnology, Institute of Microbiology of the Czech Academy of Sciences, Vídeňská 1083, 142 20 Prague 4, Czech Republic

[7] Institute for Environmental Studies, Faculty of Science, Charles University in Prague, Benátská 2, Prague 2, 128 01, Czech Republic

[8] Department of Medical Microbiology, Second Faculty of Medicine, Charles University and Motol University Hospital, V Úvalu 84, 150 06 Prague 5, Czech Republic

*Corresponding authors: alexandr.nemec@szu.gov.cz (A. Nemec), martina.kyselkova@biomed.cas.cz (M. Kyselková).

Fig. S1

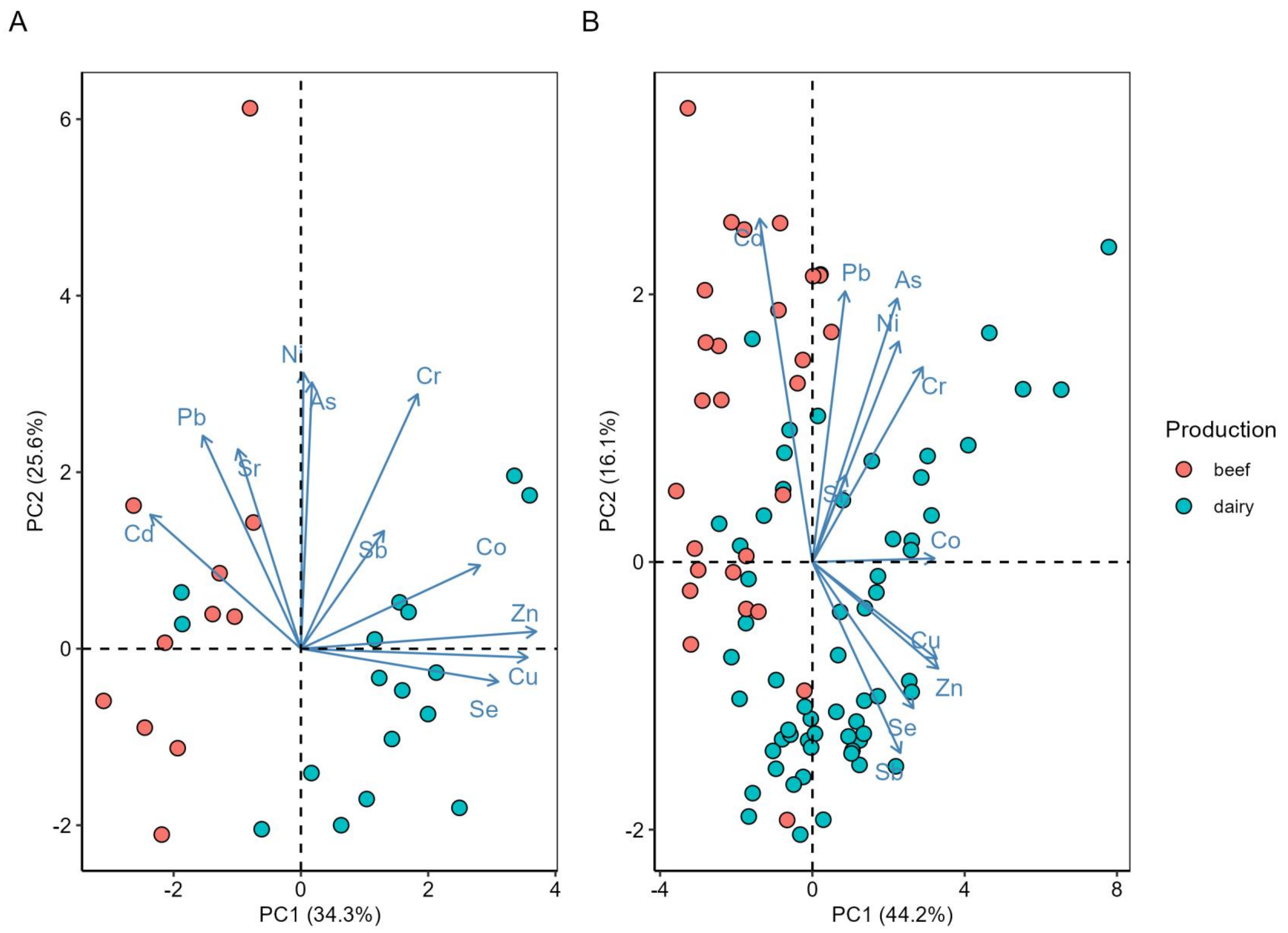

Fig. S1 Principal Component analysis (PCA) of heavy metals present in cattle feces

PCA biplot of heavy metal profiles in ‘floor’ (A) and ‘cow’ samples (B) from 28 cattle farms. Axes PC1 and PC2 represent the first and second principal components, explaining the largest proportions of variance in the dataset. Colours indicate the farm production type.

Fig. S2

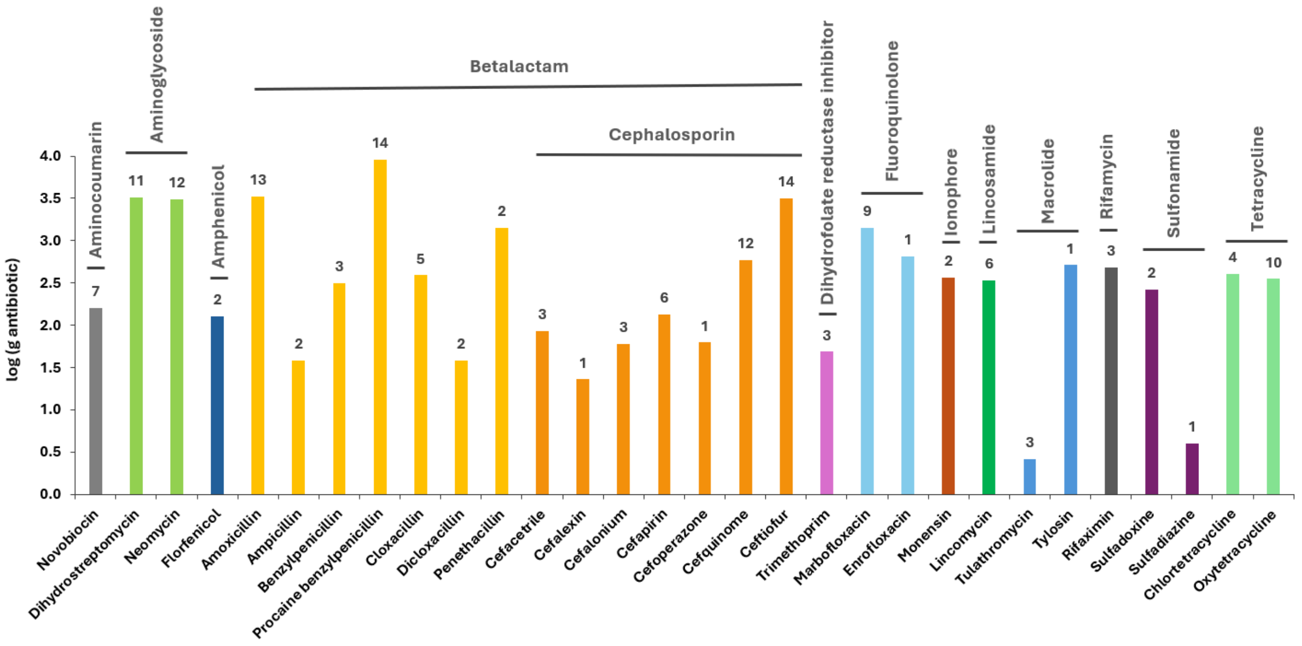

Fig. S2 Antibiotic use in the studied cattle farms

Total antibiotic use across 28 cattle farms, based on farmer-reported data from a period of six months prior to sampling (Please note that the y-axis is on the log scale). Numbers of farms using the antibiotic are indicated above the bars. The bars are colored according to the main antibiotic classes.

Fig. S3

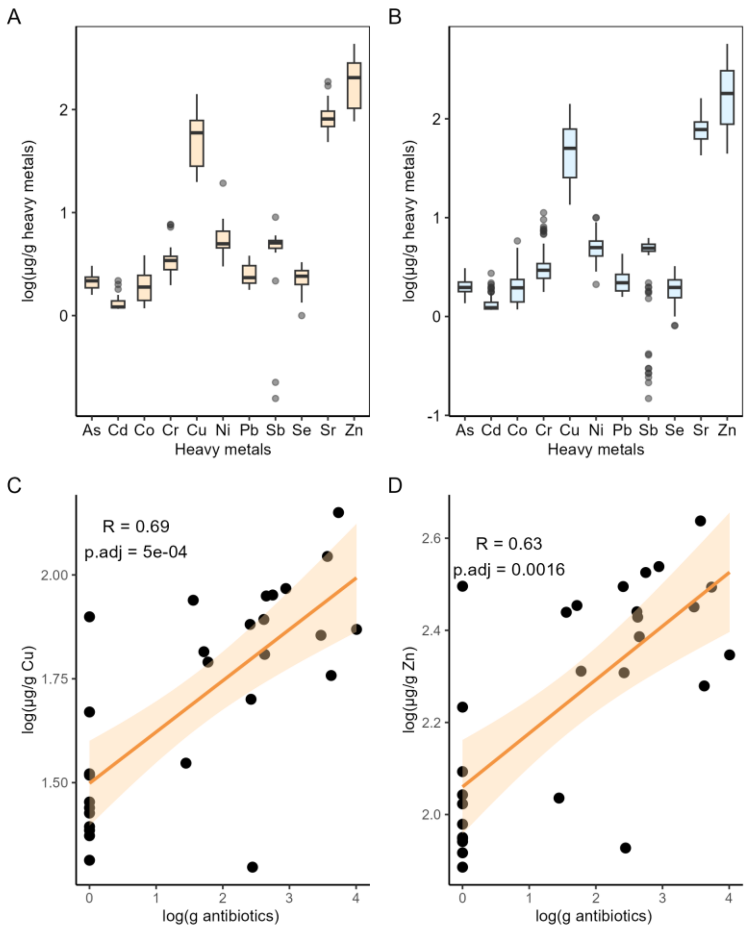

Fig. S3 Heavy metal content in cattle feces

Panels A) and B) show the distribution of heavy metal content in 'floor' (A, n=28) and 'cow' samples (B, n=93). Values on the y-axis are in log (µg g^{-1} of dry weight). Panels C) and D) show the correlation (Spearman's rank correlation test) between copper (C) or zinc (D) content in 'floor' samples and antibiotic use on farms. Values on the x-axis represent log (g total antibiotics used in 6 months preceding the farm sampling).

Fig. S4

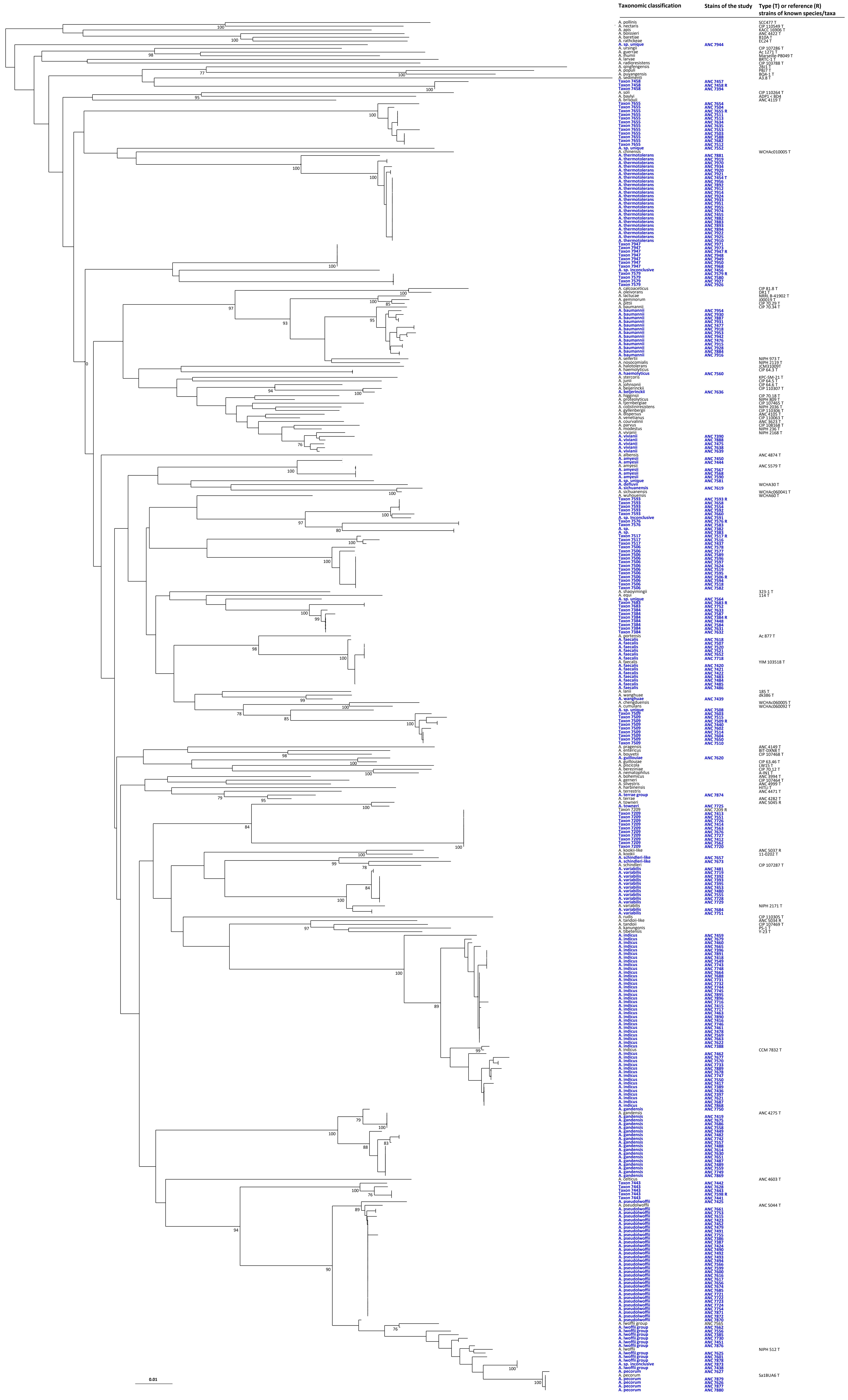

Fig. S4 Unrooted neighbor-joining tree based on 355-nt partial *rpoB* sequences of 284 strains isolated in this study and representatives of known *Acinetobacter* species. Analyses were performed with BioNumerics 7.6 (Applied Maths). Bootstrap values (> 75%) from 1000 replicates are shown at branch nodes. Scale bar represents 0.01 substitutions per nucleotide site.

Fig. S5

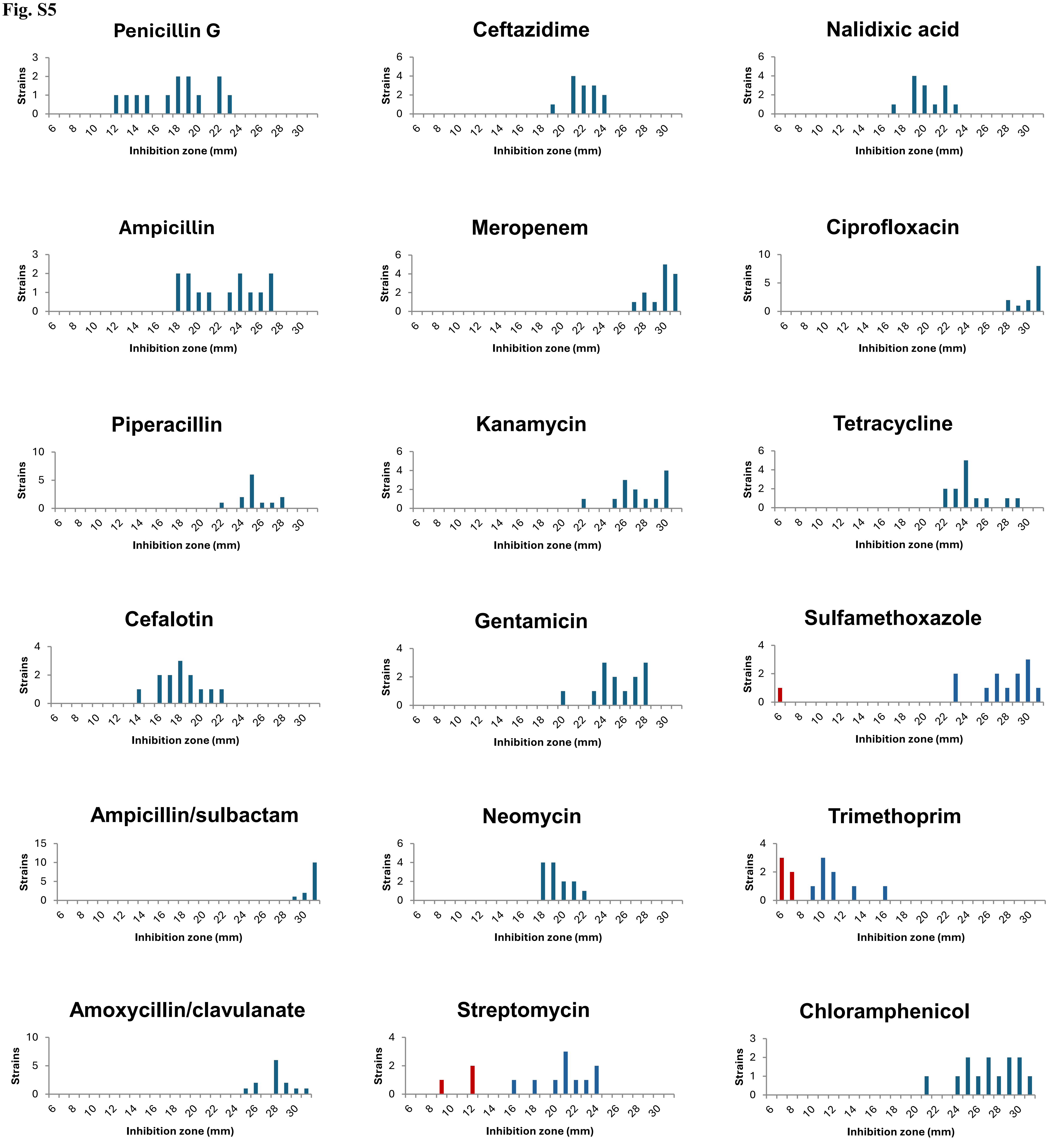

Fig. S5 Antibiotic susceptibility in *A. faecalis* strains
Distribution of inhibition zone sizes (diameters in mm) among strains of *A. faecalis* (n = 13) accross a panel of 18 antibiotics used in disk diffusion test. See Material and Methods for antibiotic amount per disk. Red bars highlight deviations from the normal (wild-type) distribution, suggesting the presence of acquired resistance mechanisms.

Fig. S6

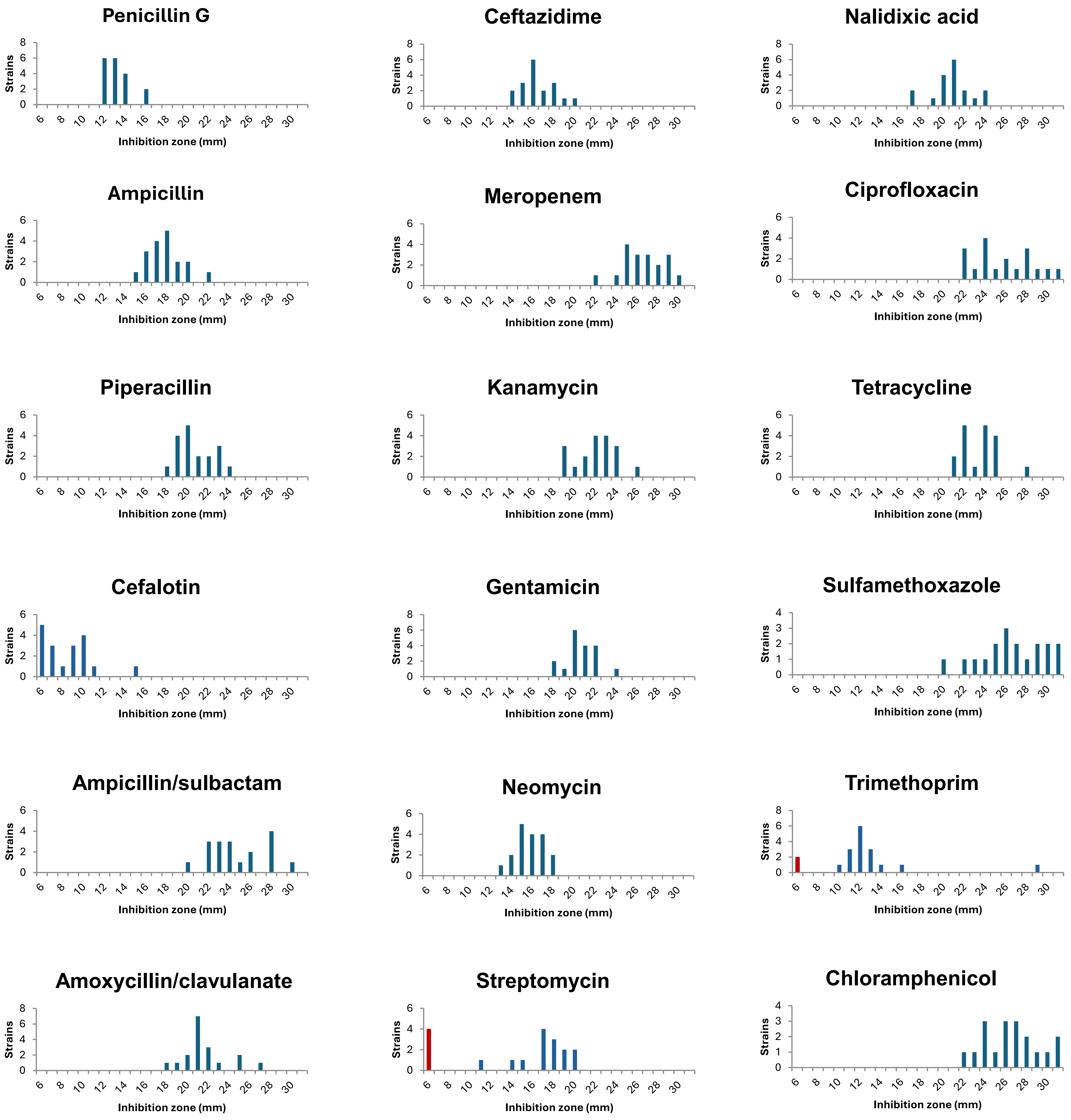

Fig. S6 Antibiotic susceptibility in *A. gandensis* strains

Distribution of inhibition zone sizes (diameters in mm) among strains of *A. gandensis* (n = 18) accross a panel of 18 antibiotics used in disk diffusion test. See Material and Methods for antibiotic amount per disk. Red bars highlight deviations from the normal (wild-type) distribution, suggesting the presence of acquired resistance mechanisms.

Fig. S7

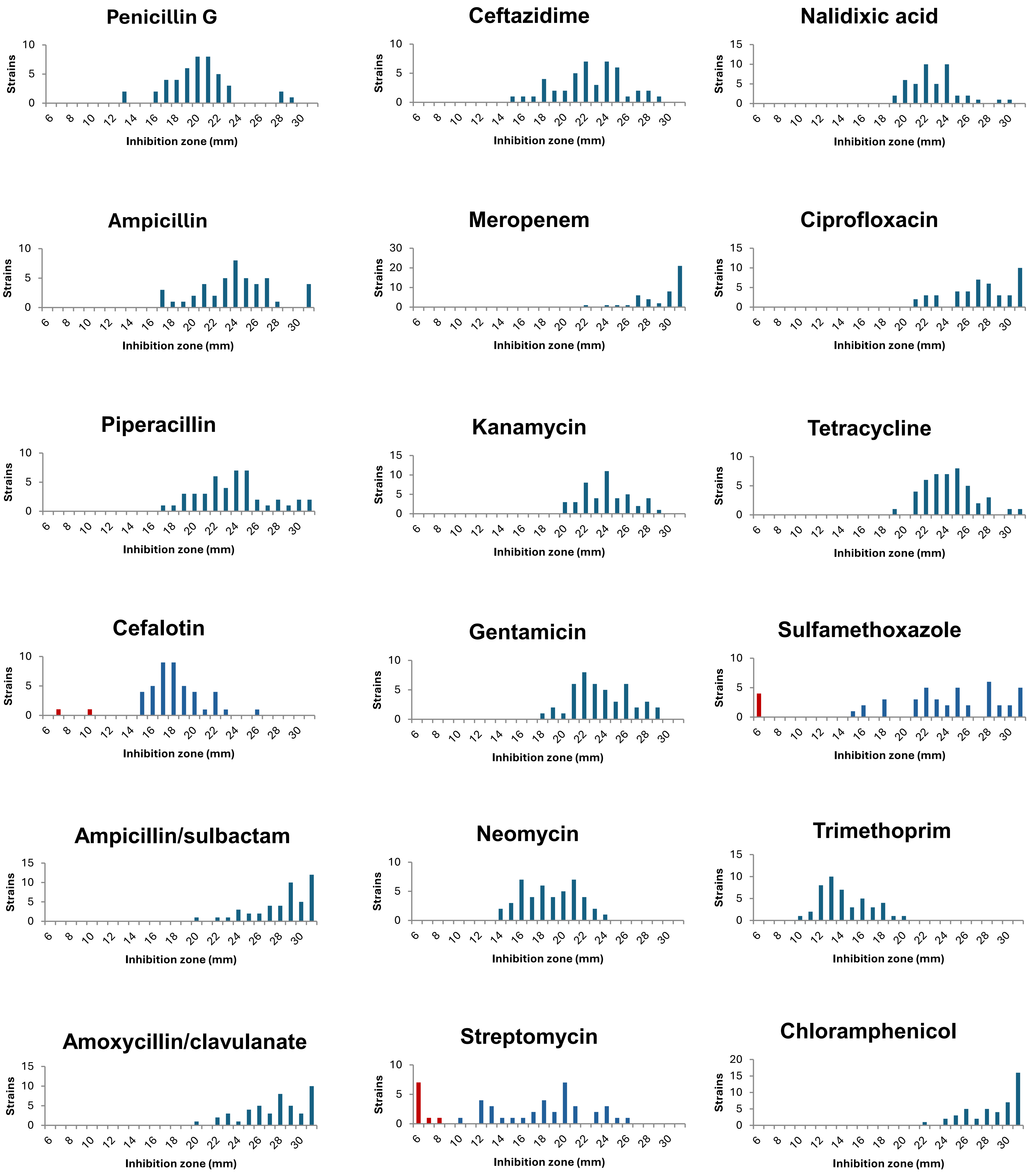

Fig. S7 Antibiotic susceptibility in *A. indicus* strains

Distribution of inhibition zone sizes (diameters in mm) among strains of *A. indicus* (n = 45) accross a panel of 18 antibiotics used in disk diffusion test. See Material and Methods for antibiotic amount per disk. Red bars highlight deviations from the normal (wild-type) distribution, suggesting the presence of acquired resistance mechanisms.

Fig. S8

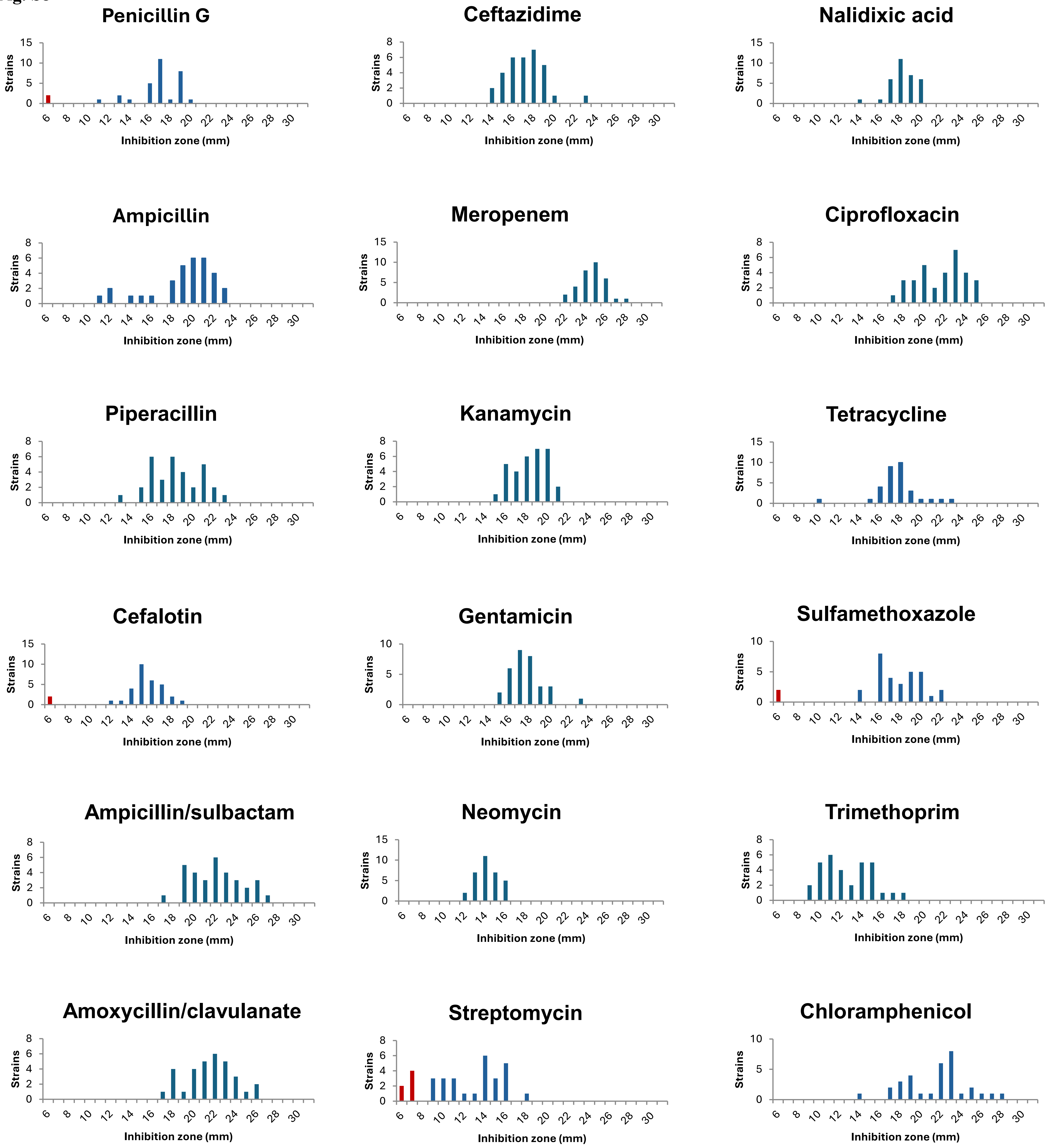

Fig. S8 Antibiotic susceptibility in *A. pseudolwoffii* strains
Distribution of inhibition zone sizes (diameters in mm) among strains of *A. pseudolwoffii* (n = 32) accross a panel of 18 antibiotics used in disk diffusion test. See Material and Methods for antibiotic amount per disk. Red bars highlight deviations from the normal (wild-type) distribution, suggesting the presence of acquired resistance mechanisms.

Fig. S9

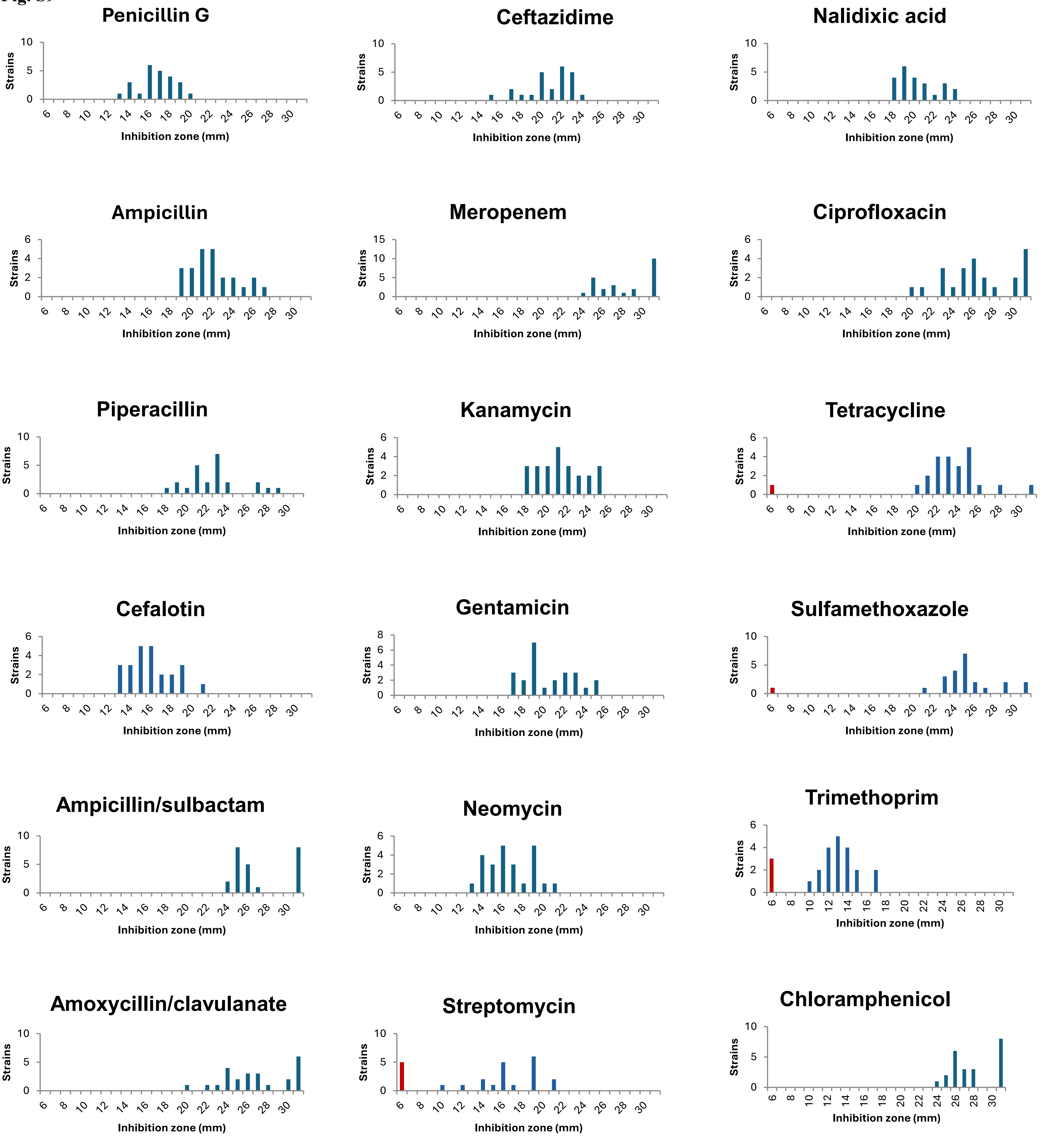

Fig. S9 Antibiotic susceptibility in *A. thermotolerans* strains

Distribution of inhibition zone sizes (diameters in mm) among strains of *A. thermotolerans* (n = 24) accross a panel of 18 antibiotics used in disk diffusion test. See Material and Methods for antibiotic amount per disk. Red bars highlight deviations from the normal (wild-type) distribution, suggesting the presence of acquired resistance mechanisms.

Fig. S10

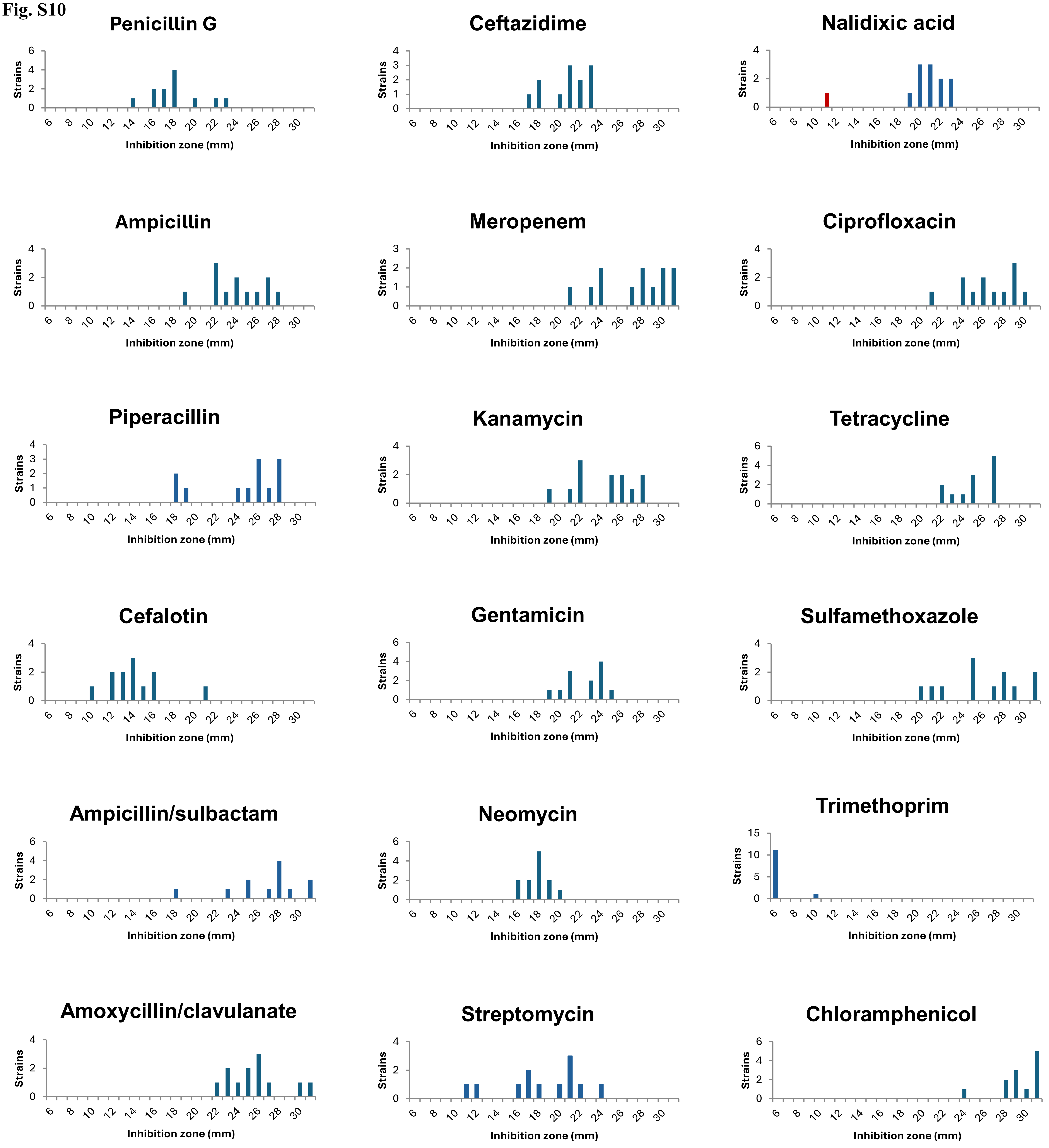

Fig. S10 Antibiotic susceptibility in *A. variabilis* strains

Distribution of inhibition zone sizes (diameters in mm) among strains of *A. variabilis* (n = 12) accross a panel of 18 antibiotics used in disk diffusion test. See Material and Methods for antibiotic amount per disk. Red bars highlight deviations from the normal (wild-type) distribution, suggesting the presence of acquired resistance mechanisms.

Fig. S11

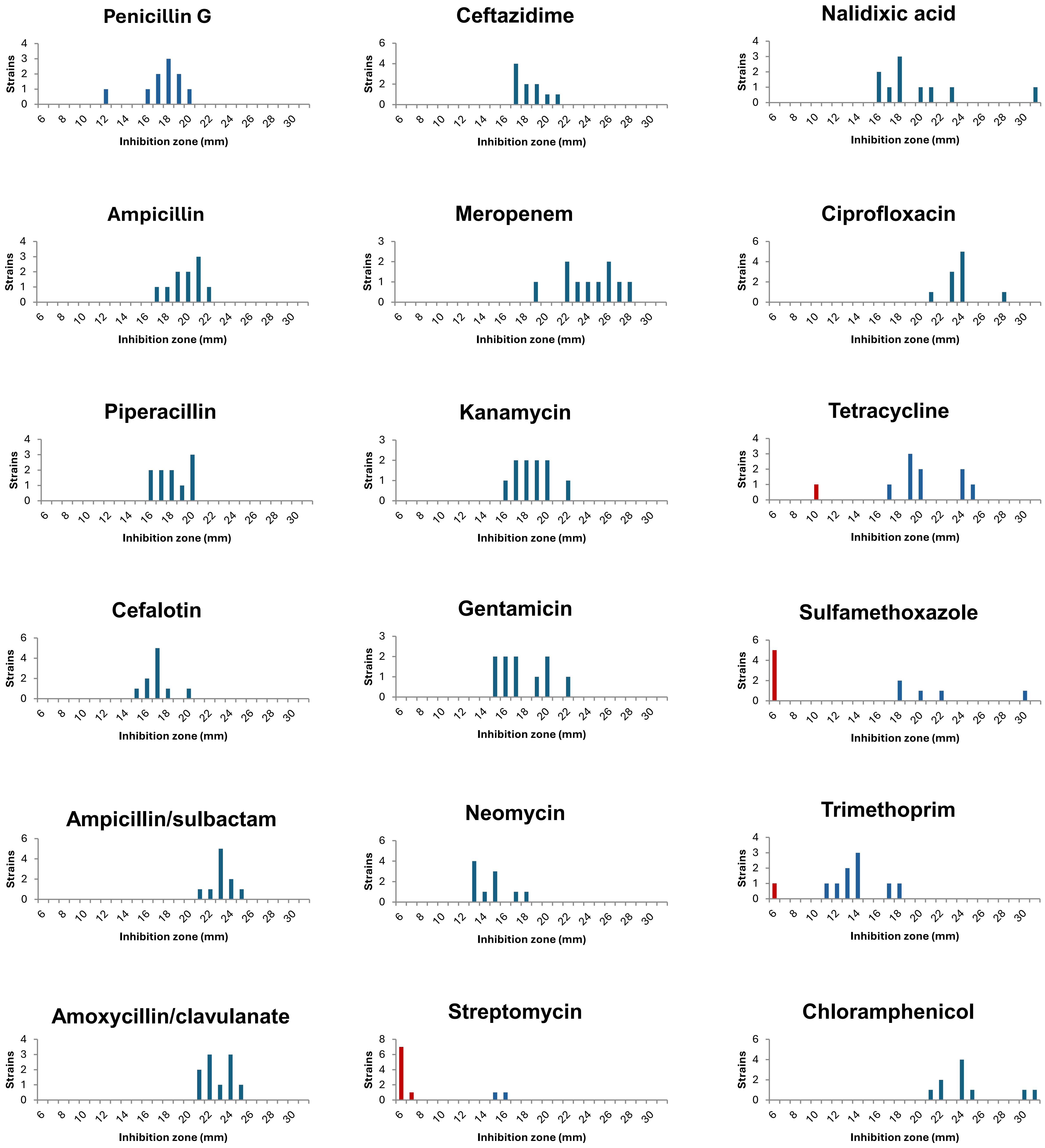

Fig. S11 Antibiotic susceptibility in Taxon 7209 strains

Distribution of inhibition zone sizes (diameters in mm) among strains of Taxon 7209 (n = 10) accross a panel of 18 antibiotics used in disk diffusion test. See Material and Methods for antibiotic amount per disk. Red bars highlight deviations from the normal (wild-type) distribution, suggesting the presence of acquired resistance mechanisms.

Fig. S12

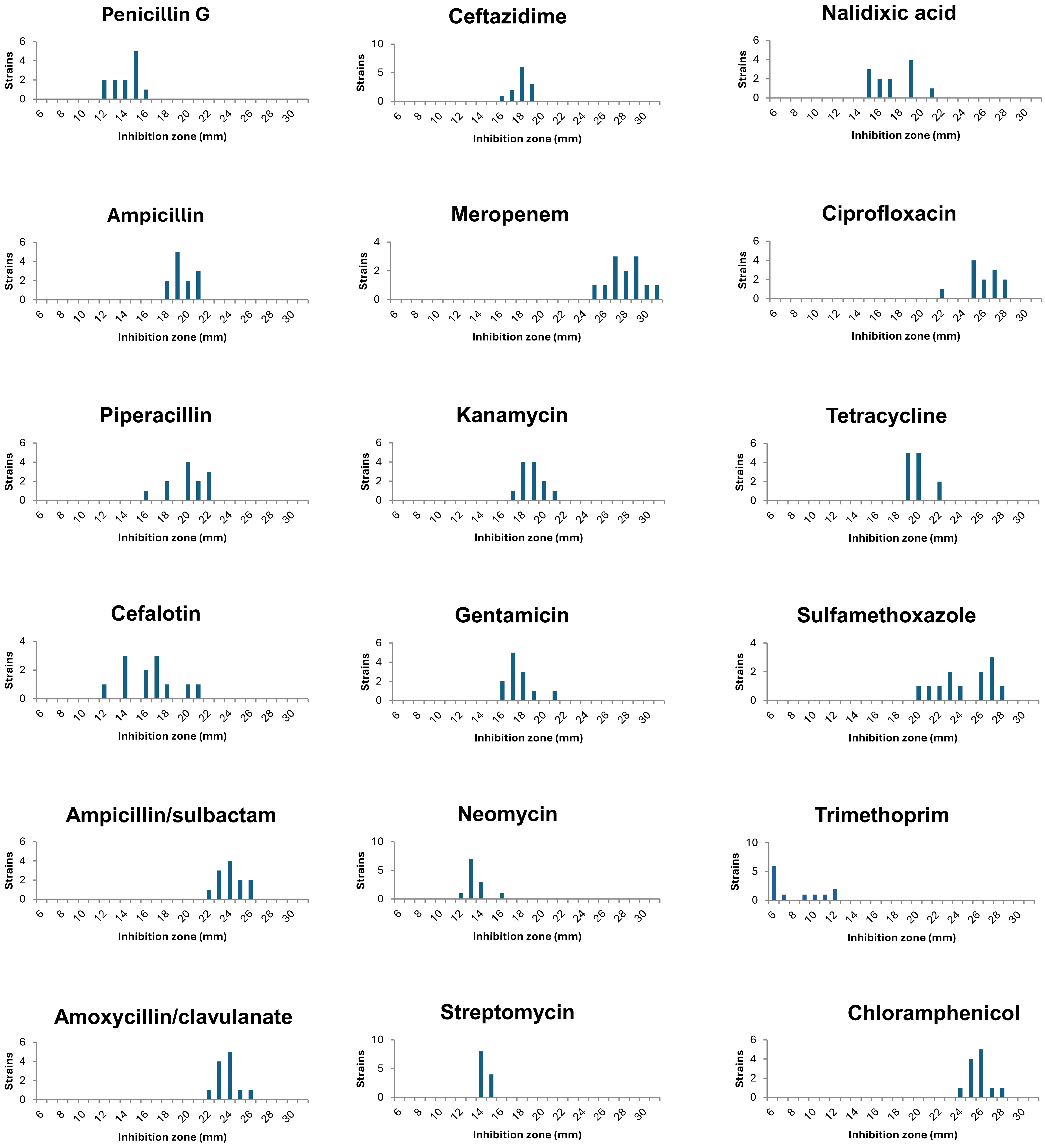

Fig. S12 Antibiotic susceptibility in Taxon 7506 strains
Distribution of inhibition zone sizes (diameters in mm) among strains of Taxon 7506 (n = 12) accross a panel of 18 antibiotics used in disk diffusion test. See Material and Methods for antibiotic amount per disk. Red bars highlight deviations from the normal (wild-type) distribution, suggesting the presence of acquired resistance mechanisms.

Fig. S13

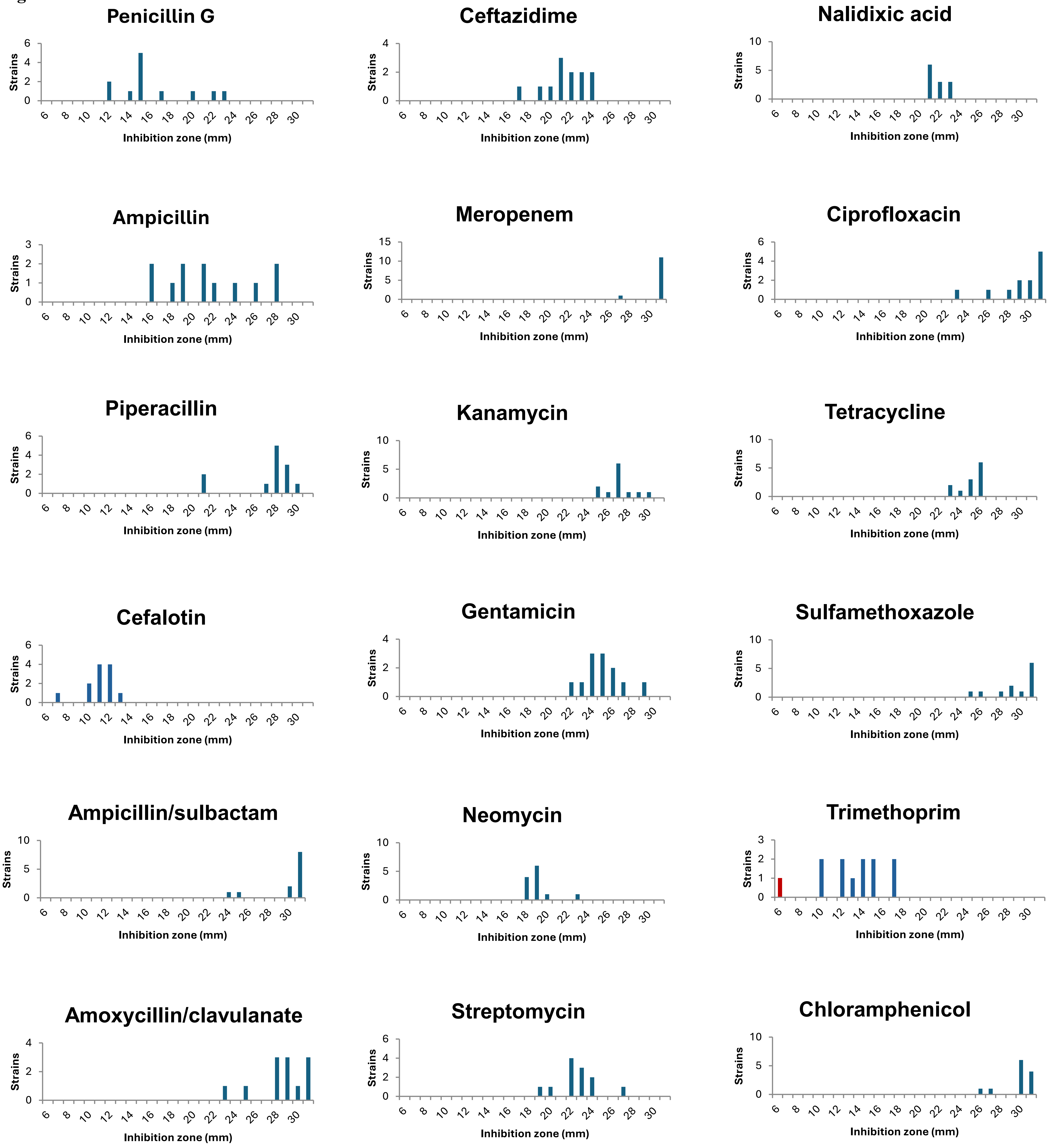

Fig. S13 Antibiotic susceptibility in Taxon 7655 strains
Distribution of inhibition zone sizes (diameters in mm) among strains of Taxon 7655 (n = 12) accross a panel of 18 antibiotics used in disk diffusion test. See Material and Methods for antibiotic amount per disk. Red bars highlight deviations from the normal (wild-type) distribution, suggesting the presence of acquired resistance mechanisms.

Fig. S14

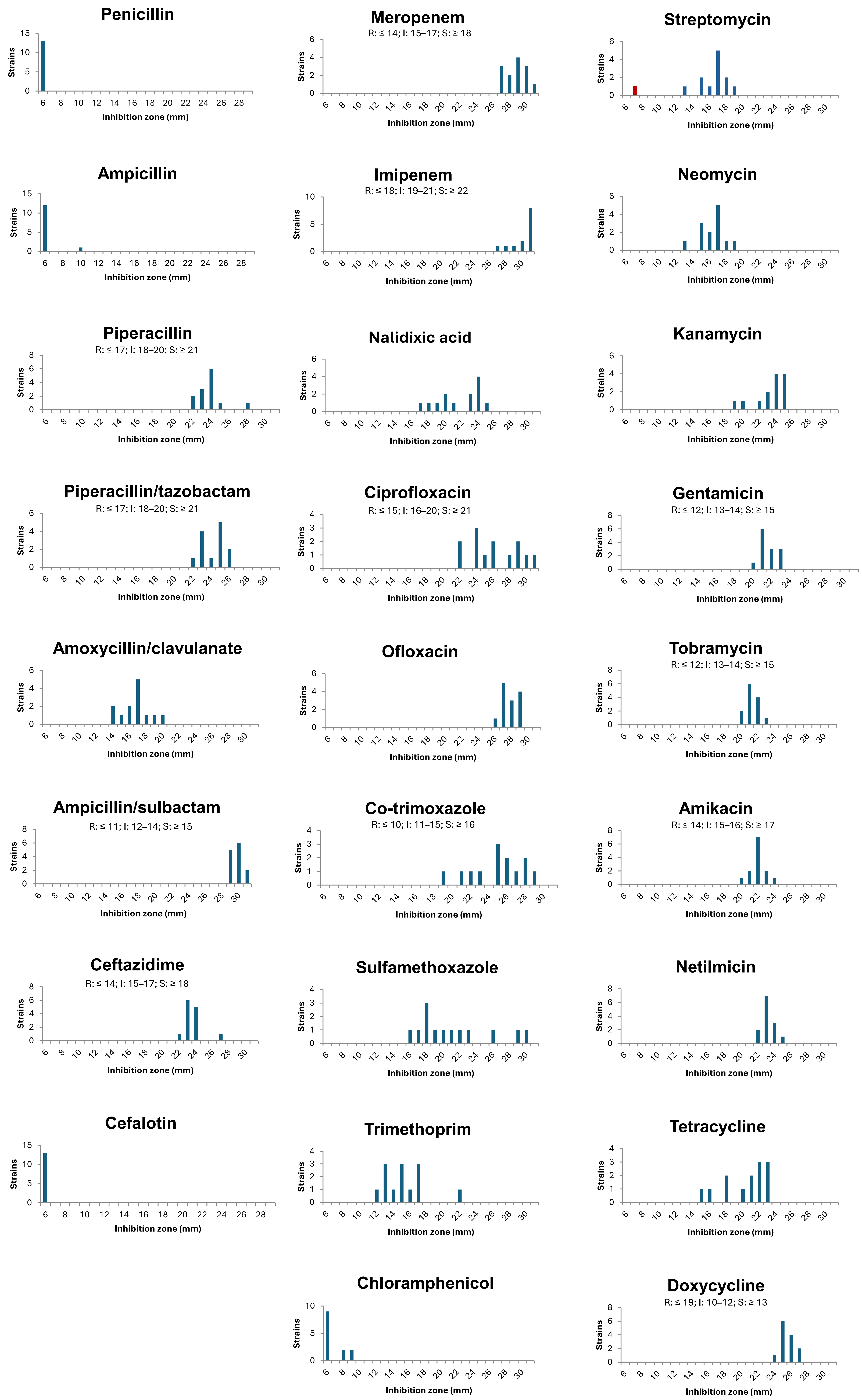

Fig. S14 Antibiotic susceptibility in *A. baumannii* strains
Distribution of inhibition zone sizes (diameters in mm) among strains of *A. baumannii* (n = 13) accross a panel of 26 antibiotics used in disk diffusion test. See Material and Methods for antibiotic amount per disk. CLSI (Clinical and Laboratory Standards Institute) breakpoints, if available, are indicated above the histograms (R – resistant, I – intermediate, S – susceptible). In other cases, red bars highlight deviations from the normal (wild-type) distribution, suggesting the presence of acquired resistance mechanisms. Please note that *A. baumannii* is wild-type resistant to penicillin, ampicillin, cefalotin and chloramphenicol.

Fig. S15

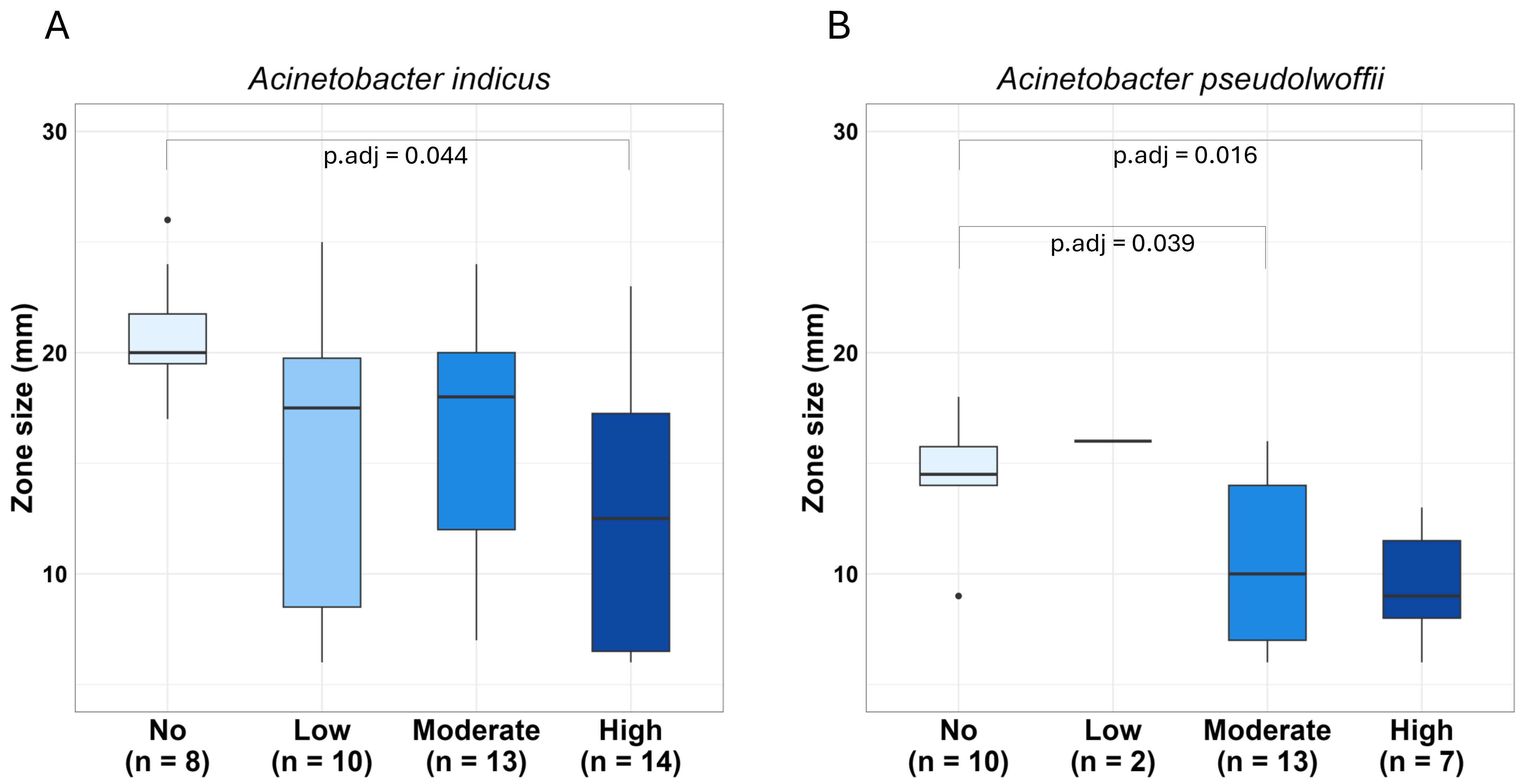

Fig. S15 Susceptibility to streptomycin in *Acinetobacter* isolates across on-farm antibiotic use categories.

Analysis of streptomycin susceptibility in strains of *A. indicus* (A) and *A. pseudolwoffii* (B). Streptomycin inhibition zone size was compared between isolates from farms with no (0 g), low (< 100 g), moderate (100-1,000 g) and high (> 1,000 g) antibiotic use (per-farm antibiotic use within six months prior sampling), using Kruskal-Wallis test at $p < 0.05$ and Wilcoxon rank-sum test for post-hoc pairwise comparisons (p.adj is Benjamini–Hochberg-corrected p-value). Boxes represent the interquartile range from Q1 to Q3, with median drawn as a horizontal line. Whiskers represent the smallest and largest values within 1.5 times the interquartile range from the quartiles. Outliers are drawn as points.

Fig. S16

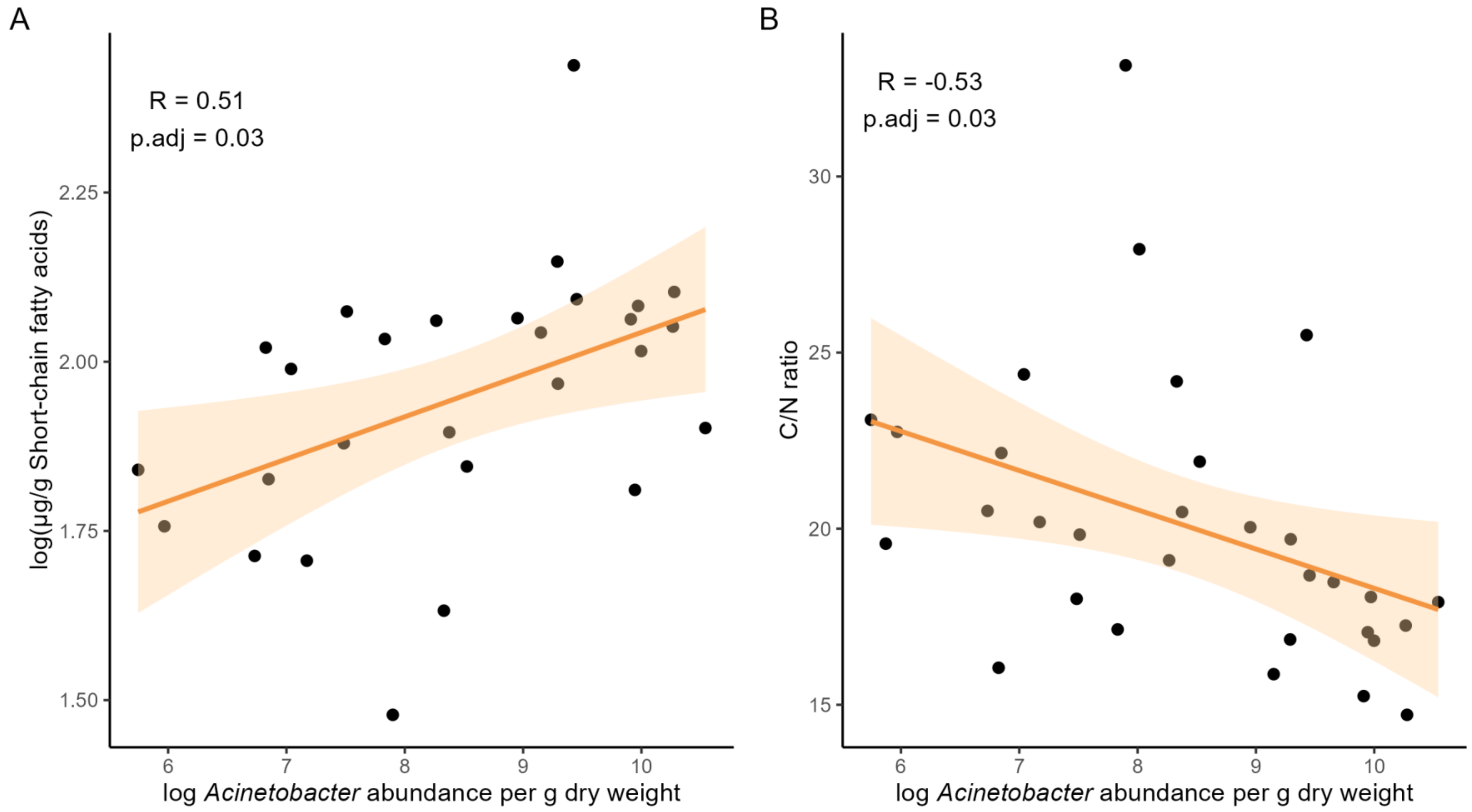

Fig. S16 Correlation between *Acinetobacter* abundance and total short-chain fatty acids and carbon-to-nitrogen ratio

Spearman's rank correlation between *Acinetobacter* abundance and total short-chain fatty acids (A) or carbon-to-nitrogen ratio (B) in 'floor' samples. Please note that the axes representing 'total short-chain fatty acids' and '*Acinetobacter* abundance per g dry weight' are on the log scale.

Fig. S17

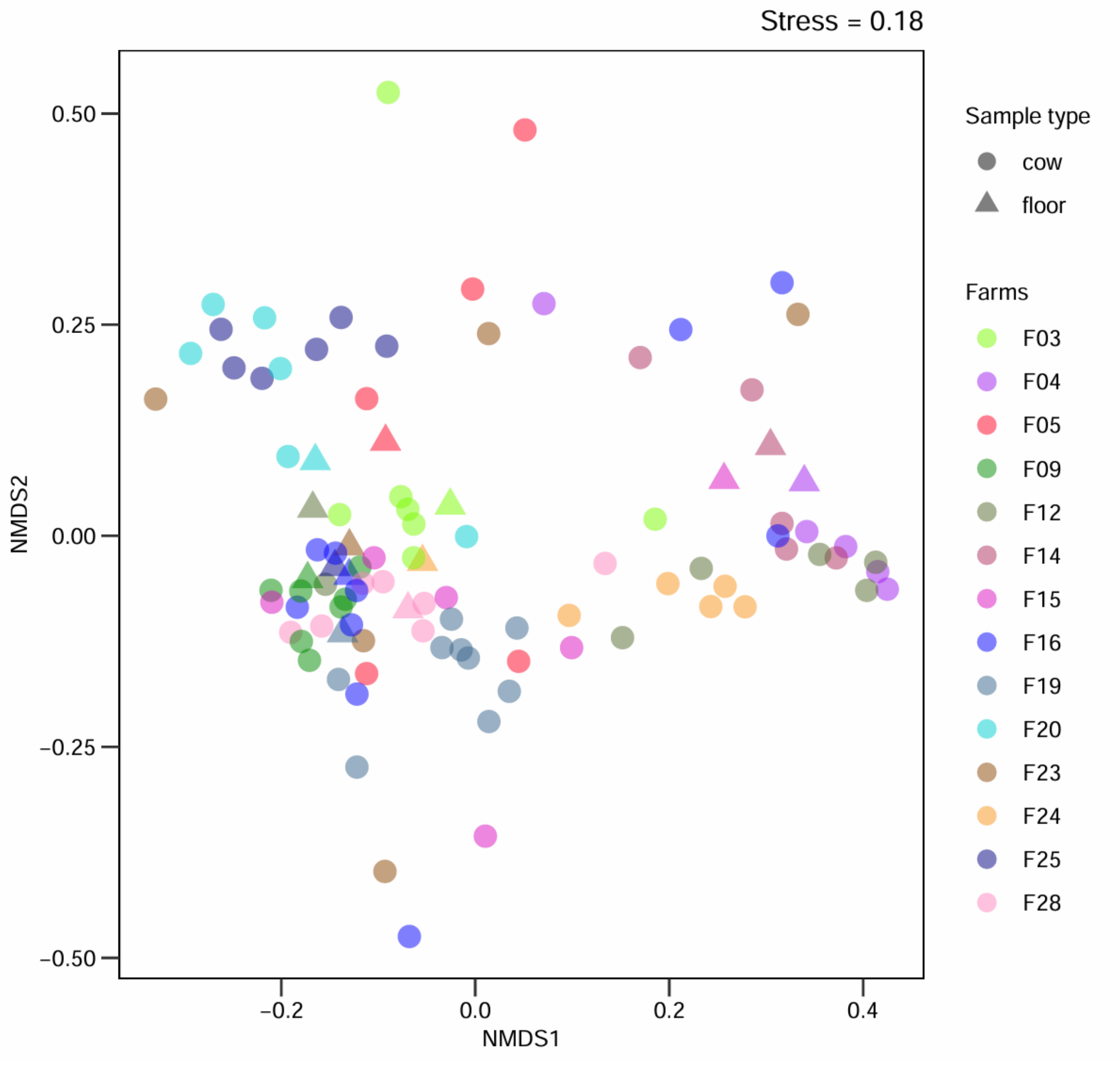

Farms	z -scores of floor samples
F03	−0.73
F04	−0.86
F05	−1.60
F09	−0.42
F12	1.98
F14	−1.63
F15	3.51
F16	−0.95
F19	1.20
F20	−0.29
F23	−1.78
F24	5.65
F25	8.08
F28	−1.03

Fig. S17 Non-metric multidimensional scaling (NMDS) analysis of *Acinetobacter* communities from 14 cattle farms with both ‘floor’ and ‘cow’ sample types available
NMDS plot based on the presence-absence of *Acinetobacter rpoB* clusters from ‘cow’ samples and corresponding ‘floor’ samples from the same farm. Z-scores of ‘floor’ samples are calculated relative to the distances of ‘cow’ samples to the farm-specific centroids.

Fig. S18

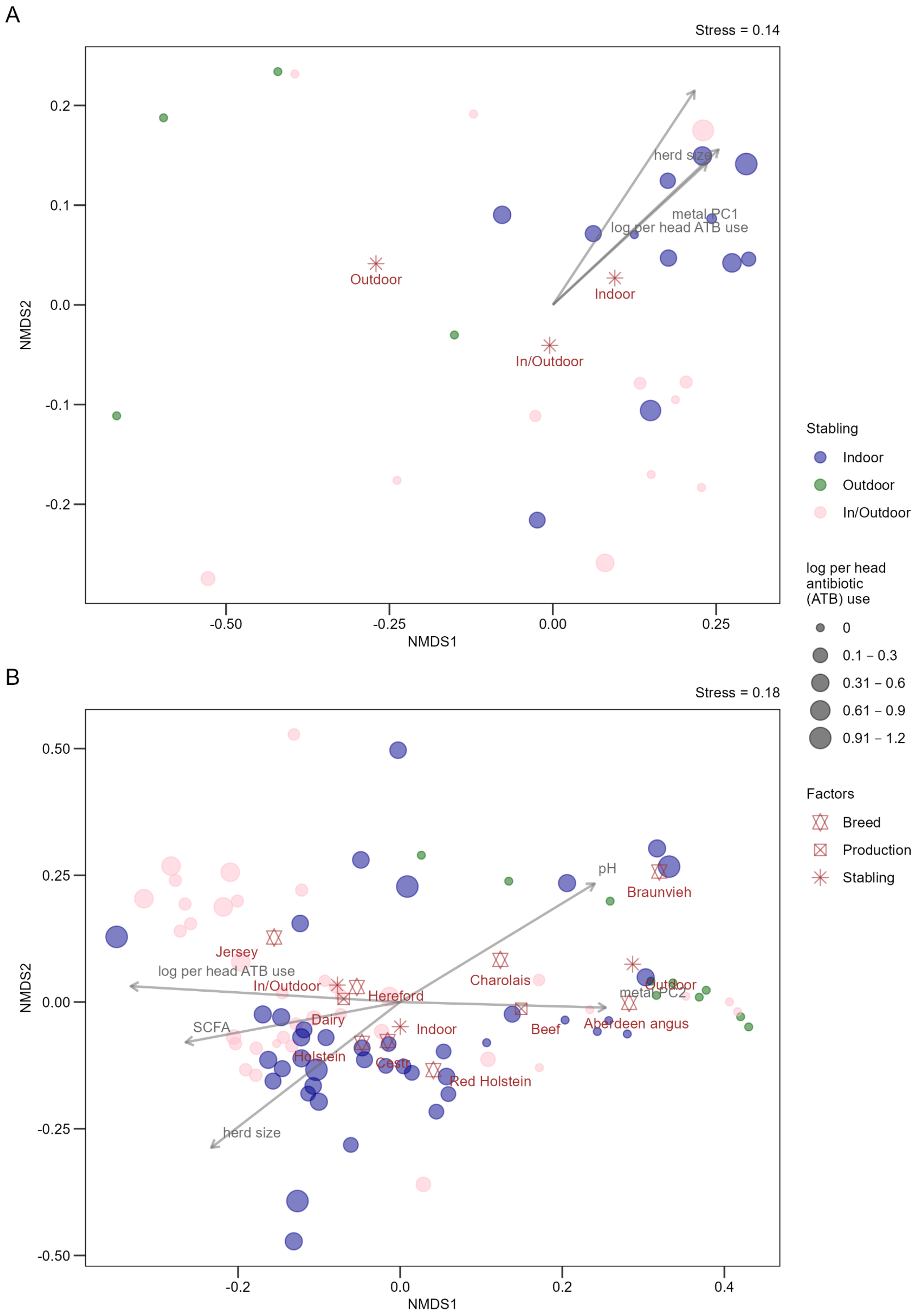

Fig. S18 Non-metric multidimensional scaling (NMDS) analysis of *Acinetobacter* communities from 28 cattle farms, labeled by stabling types

NMDS plots based on the presence-absence of *Acinetobacter rpoB* clusters from 'floor' (A) and 'cow'(B) samples. The 'floor' samples originated from all 28 farms and the 'cow' samples originated from a subset of 14 farms. The stabling is represented by colour. Data point size represents log-transformed per-head antibiotic use (originally measured in g per 6 months). Farm- and cow-related explanatory variables significantly correlated with the ordinations (envfit, $p < 0.05$) are shown as vectors (numerical) or centroids (categorical). Abbreviations: SCFA, short-chain fatty acids; log per head ATB use, log-transformed per-head antibiotic use; metal PC1 and PC2, the first two principal components summarizing heavy metal variation (see Fig. S1).

Fig. S19

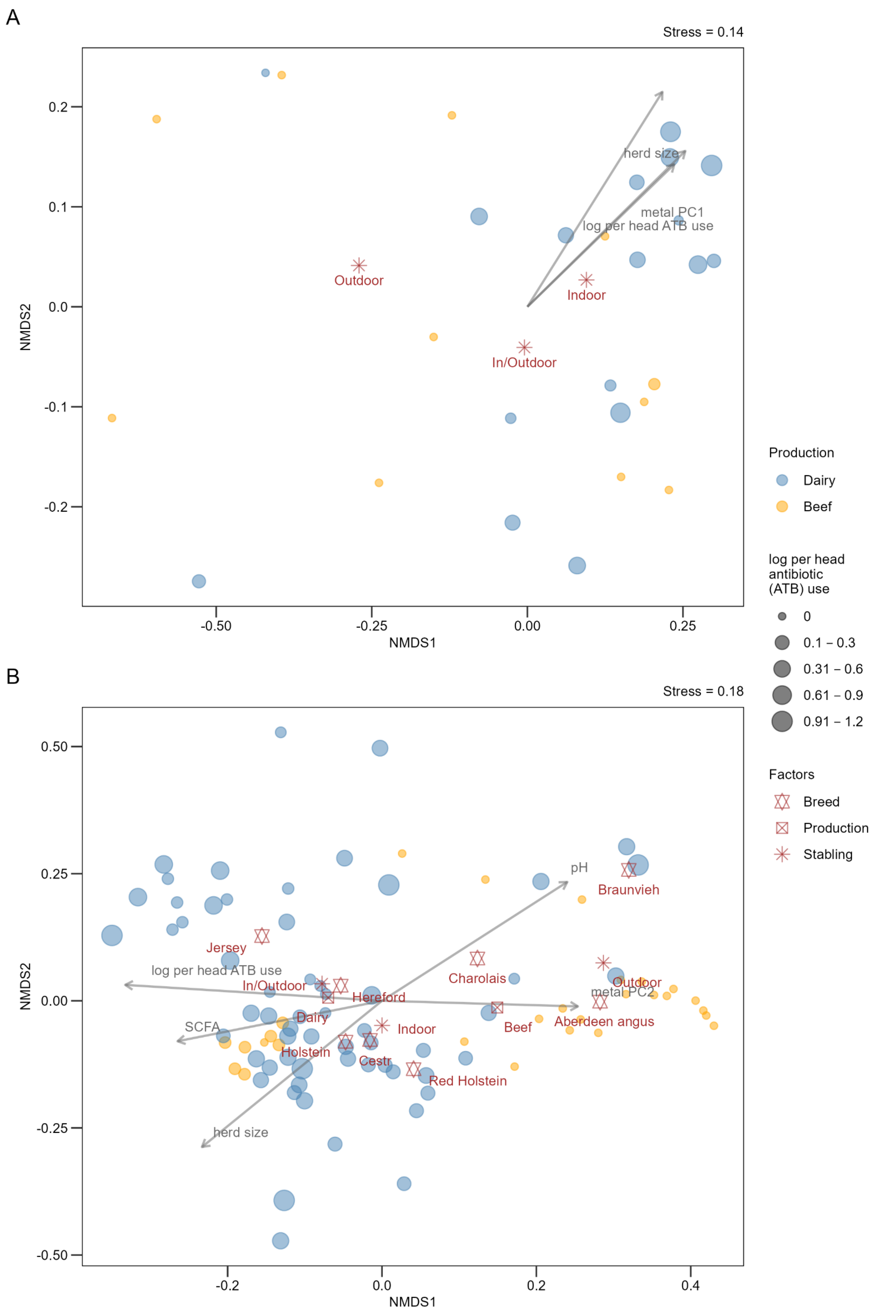

Fig. S19 Non-metric multidimensional scaling (NMDS) analysis of *Acinetobacter* communities from 28 cattle farms, labeled by production type

NMDS plots based on the presence-absence of *Acinetobacter rpoB* clusters from 'floor' (A) and 'cow'(B) samples. The 'floor' samples originated from all 28 farms and the 'cow' samples originated from a subset of 14 farms. The production is represented by colour. Data point size represents log-transformed per-head antibiotic use (originally measured in g per 6 months). Farm- and cow-related explanatory variables significantly correlated with the ordinations (envfit, $p < 0.05$) are shown as vectors (numerical) or centroids (categorical). Abbreviations: SCFA, short-chain fatty acids; log per head ATB use, log-transformed per-head antibiotic use; metal PC1 and PC2, the first two principal components summarizing heavy metal variation (see Fig. S1).

Fig. S20

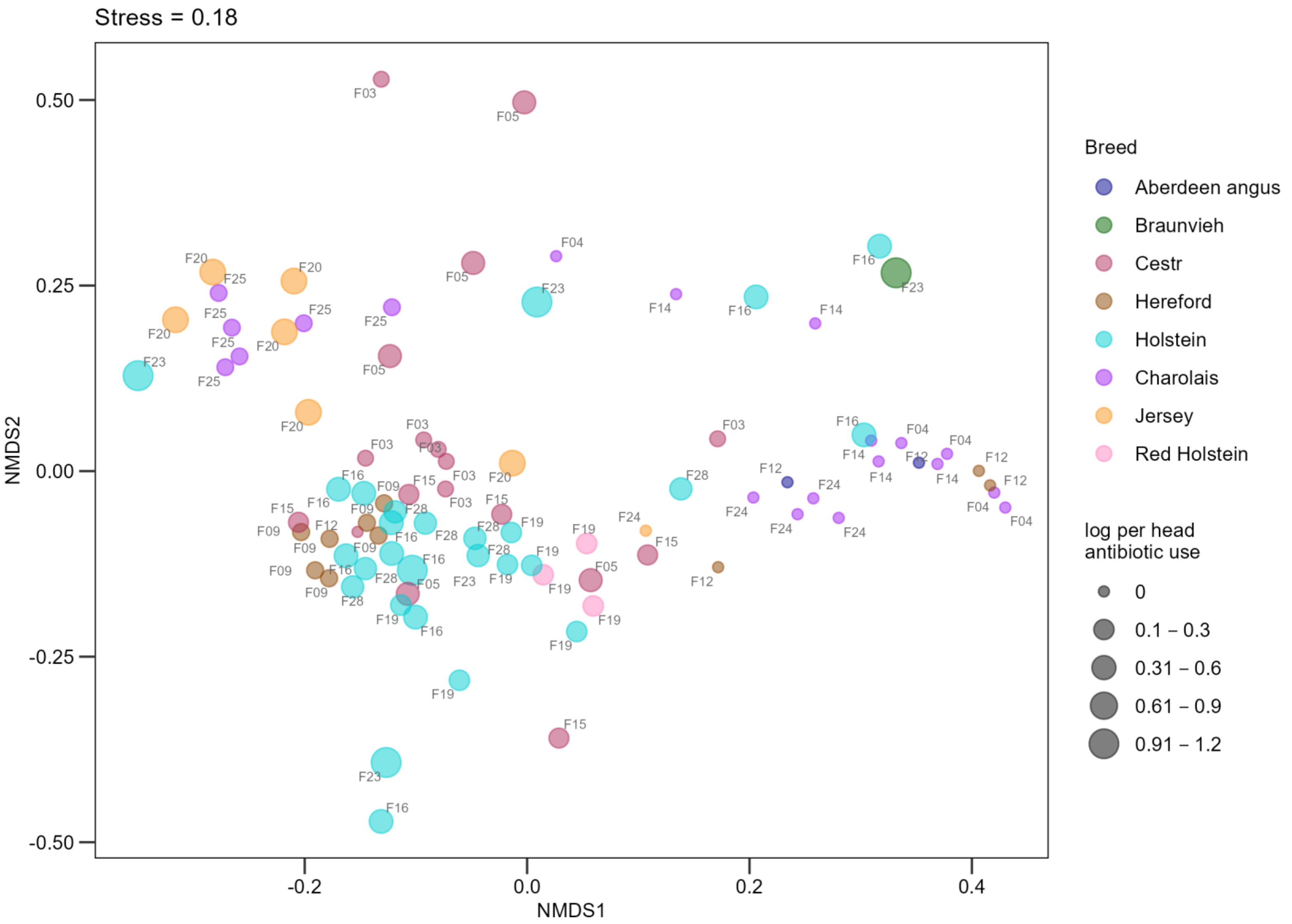

Fig. S20 Non-metric multidimensional scaling (NMDS) analysis of *Acinetobacter* communities in 'cow' samples, labeled by cow breeds

NMDS plots based on the presence-absence of *Acinetobacter rpoB* clusters from 'cow' samples. The 'cow' samples originated from a subset of 14 farms and are labeled with farm number. The breed is represented by colour. Data point size represents log-transformed per-head antibiotic use (originally measured in g per 6 months).

Fig. S21

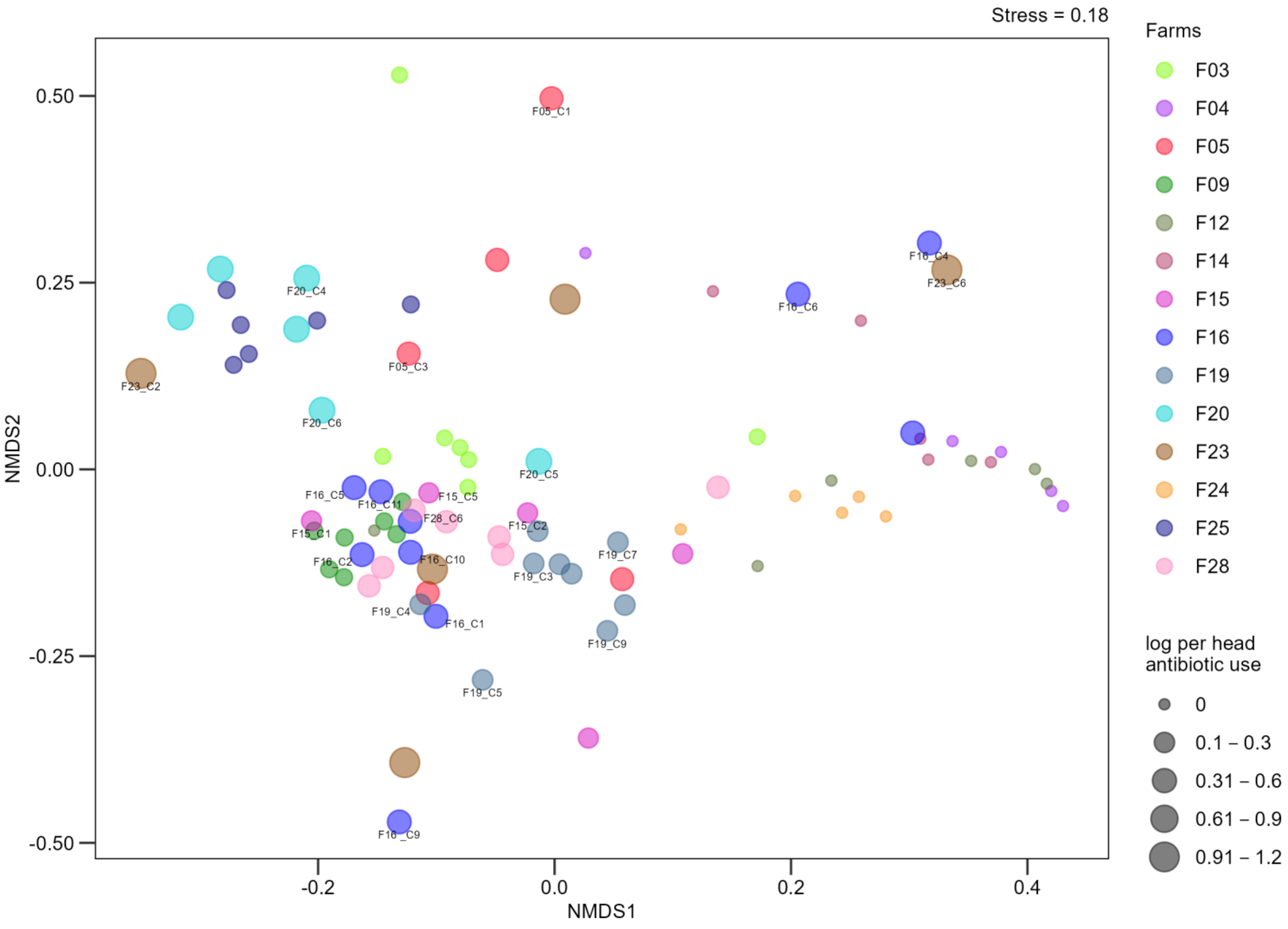

Fig. S21 Non-metric multidimensional scaling (NMDS) analysis of *Acinetobacter* communities in 'cow' samples showing antibiotic administration

NMDS plots based on the presence-absence of *Acinetobacter rpoB* clusters from 'cow' samples. The 'cow' samples originated from a subset of 14 farms and the farm of origin is represented by colour. The samples labeled with cow number indicate antibiotic administration to the individual cow within 6 months prior to sampling. Data point size represents log-transformed per-head antibiotic use (originally measured in g per 6 months).

Fig.S22

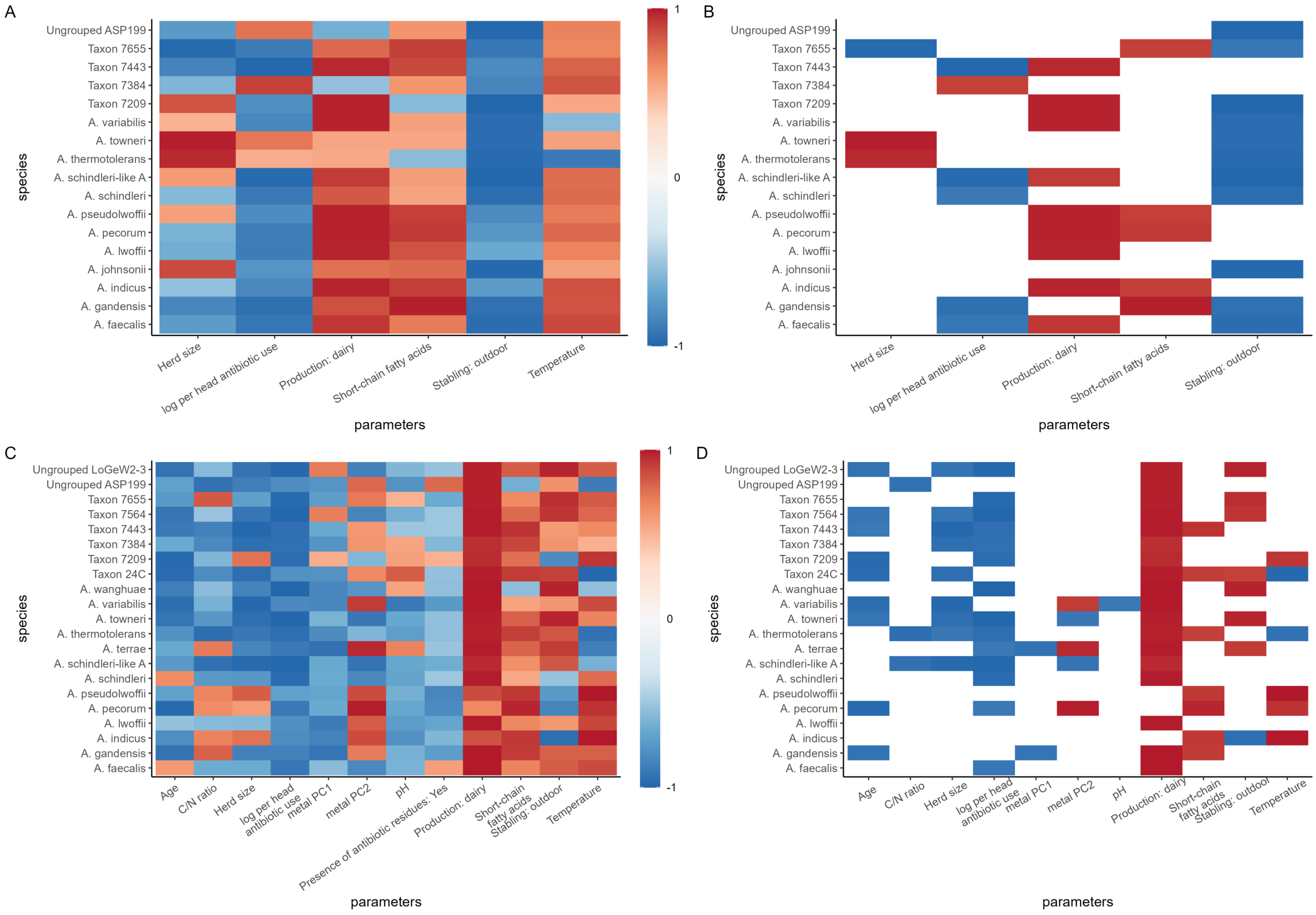

Fig. S22 Association of *Acinetobacter* species with environmental parameters, based on Hierarchical Modelling of Species Communities (HMSC)
Heat maps of β-parameter estimates showing the responses of *Acinetobacter* species from 'floor' (A and B) and 'cow (C and D) samples to environmental factors. The explanatory variables in (A) and (B) included farm-level factors (production type, stabling, herd size, log-transformed per-head antibiotic use, air temperature, and total short-chain fatty acids (= SCFA)). The explanatory variables in (C) and (D) included farm-level factors (as above, except total SCFA), cow-level factors (age, pH, C/N ratio, heavy metal Principal Component 1 and 2 (= metal PC1 and PC2), presence of antibiotic residues, and total SCFA). In (A) and (C), all the β parameters are displayed while in (B) and (D), only the β parameters with at least 90% posterior probability are displayed, with positive support values in red and negative support values in blue. Log of sequencing depth is not displayed, but was included as an explanatory variable in the HMSC models to account for the varied sequencing depth.

Fig. S23

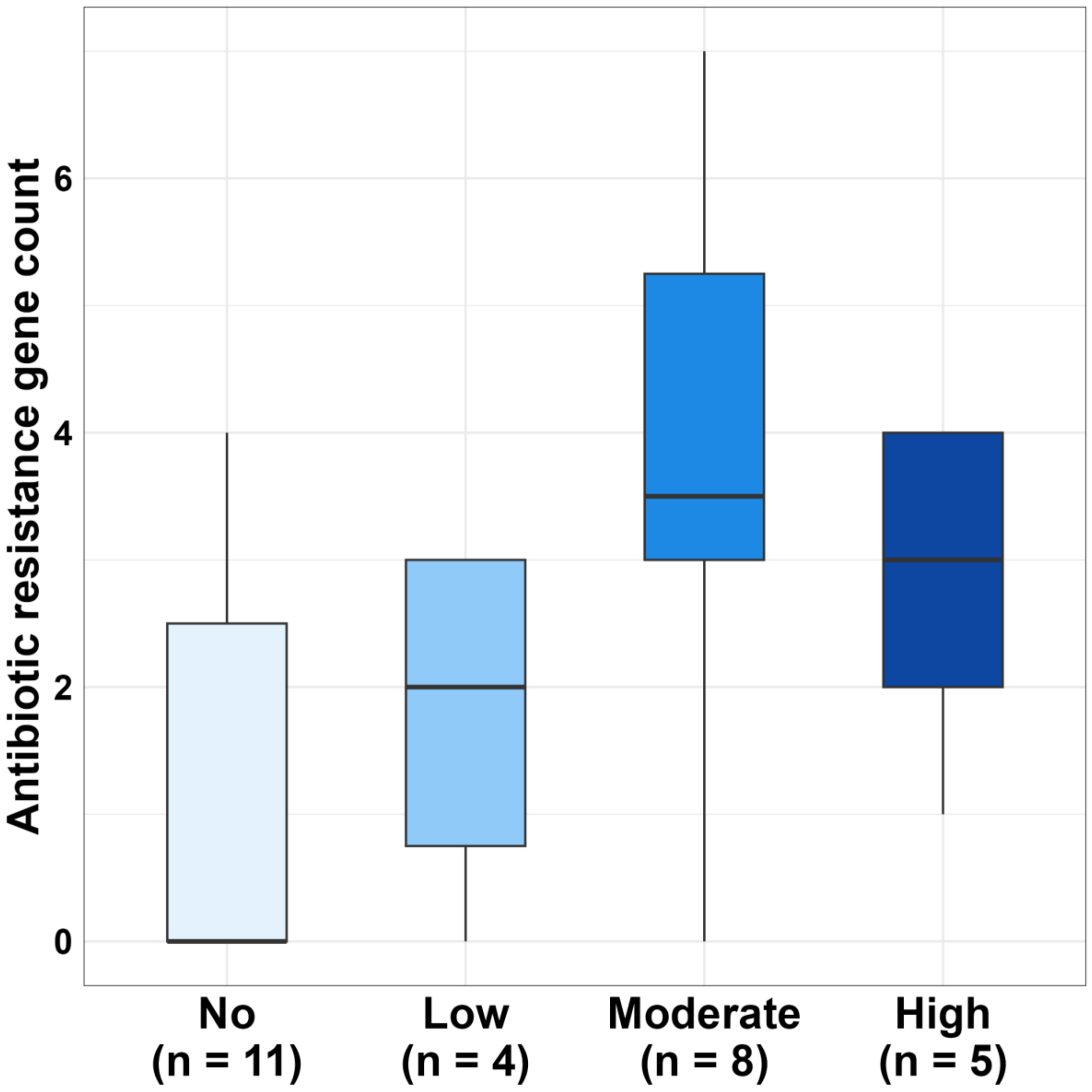

Fig. S23 Antibiotic resistance gene richness across on-farm antibiotic use categories

Numbers of unique antibiotic resistance genes detected per farm, as compared across farms with no (0 g), low (< 100 g), moderate (100-1,000 g) and high (> 1,000 g) antibiotic use (per-farm antibiotic use within six months prior sampling). Boxes represent the interquartile range from Q1 to Q3, with median drawn as a horizontal line. Whiskers represent the smallest and largest values within 1.5 times the interquartile range from the quartiles. Outliers were not detected. The Kruskal–Wallis test indicated a significant difference among groups (p = 0.03337), but subsequent pairwise comparisons using the Wilcoxon rank-sum test with Benjamini–Hochberg-correction did not reveal any statistically significant differences between individual groups (all adjusted $p > 0.05$).

Fig. S24

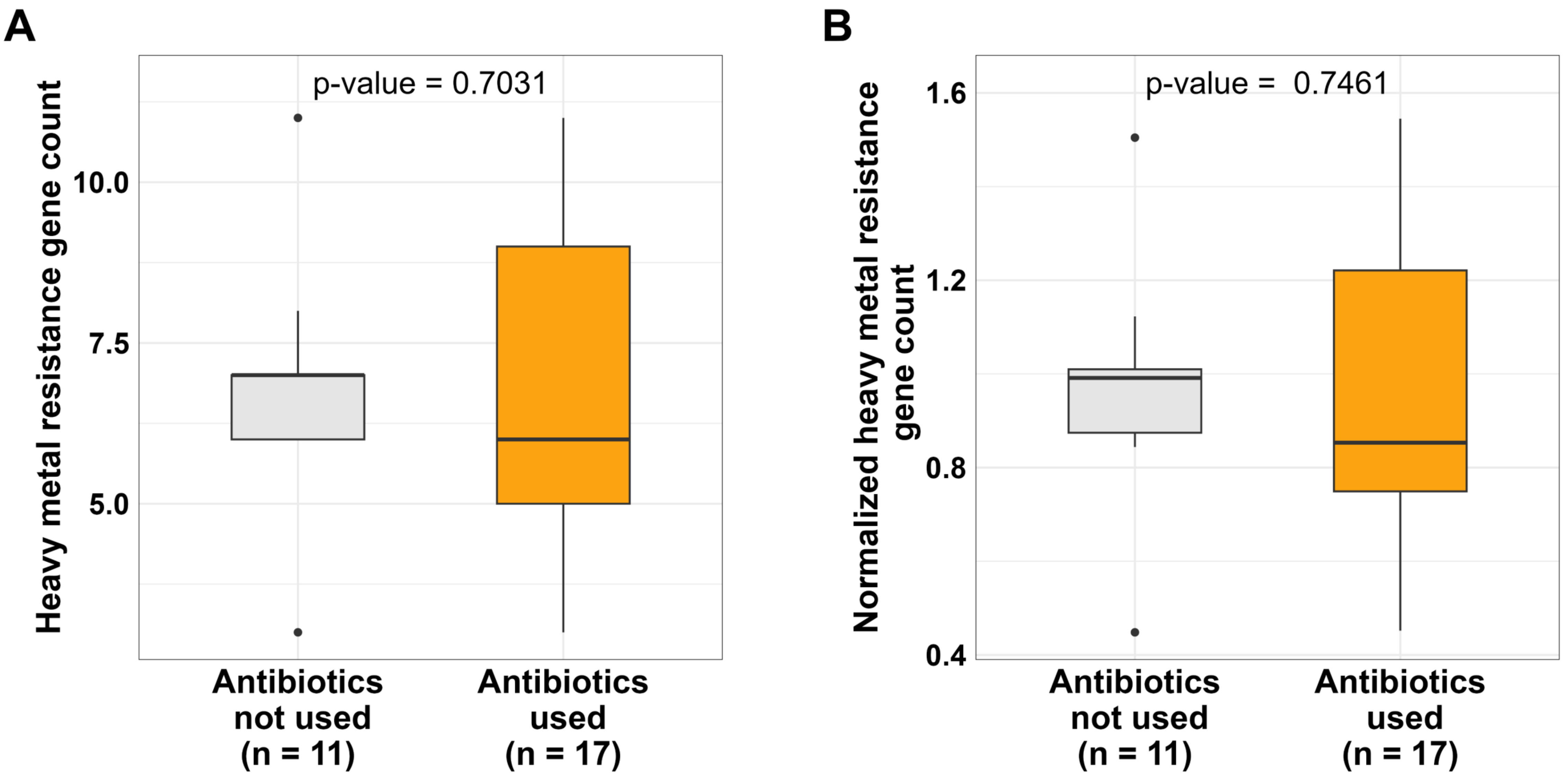

Fig. S24 Heavy metal resistance genes in *Acinetobacter* shotgun metagenome

A) Numbers of individual heavy metal resistance genes detected per farm, compared between antibiotic-using ("Antibiotics used") and antibiotic-free ("Antibiotics not used") farms, with Wilcoxon rank-sum test. B) shows the same data as plot A, but normalized by log(assembly length). Boxes represent the interquartile range from Q1 to Q3, with median drawn as a horizontal line. Whiskers represent the smallest and largest values within 1.5 times the interquartile range from the quartiles. Outliers are drawn as points.

Fig. S25

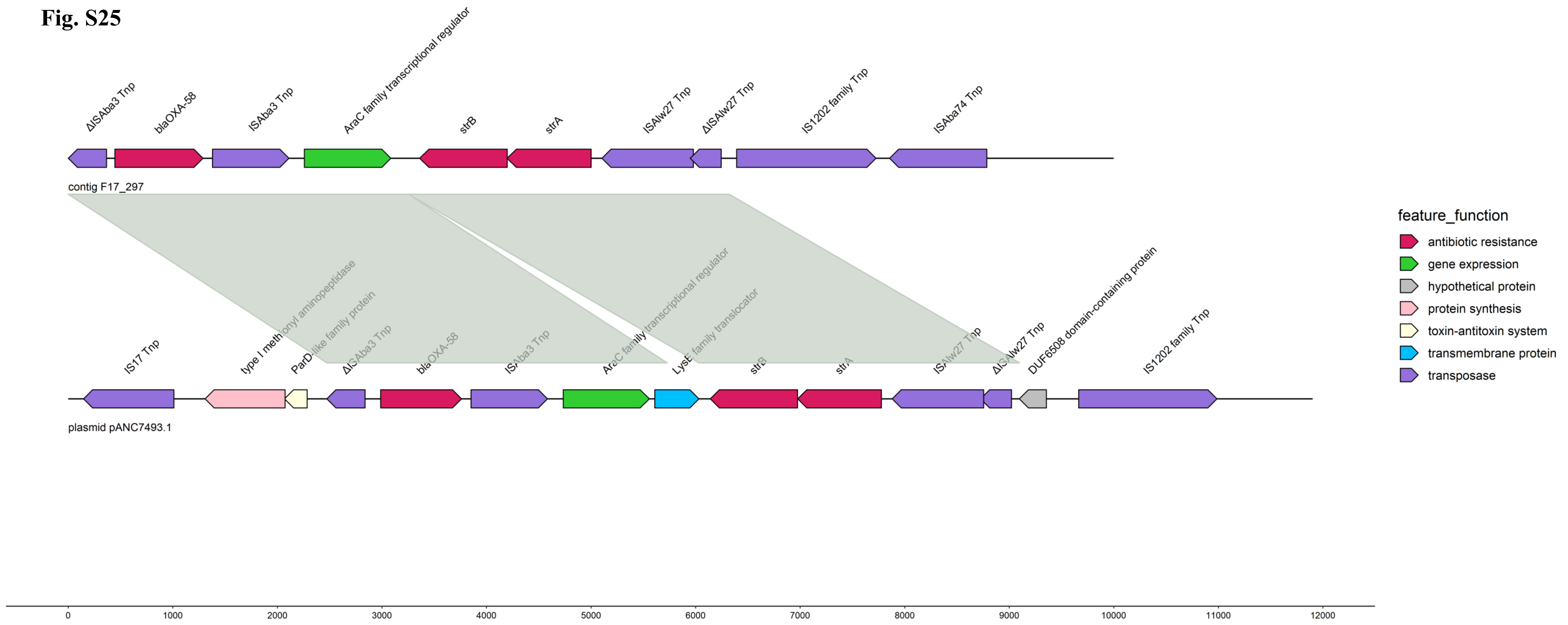

Fig. S25 Sequence comparison of contig F17_297 and plasmid pANC7493.1

Comparison of regions containing the antibiotic resistance genes *bla*$_{OXA\text{-}58}$, *strA* and *strB* in contig F17_297 and plasmid pANC7493.1. The total length of the contig is 35,741 bp, of which only the first 10,000 bp are shown. The total length of the plasmid is 167,549 bp, of which only 11,903 bp (corresponding to the region 101,491–91,514 bp) are shown. The gray shading indicates the regions sharing >99.9% nucleotide identity.
